# Clinical outcomes in pediatric intestinal failure: a meta-analysis and meta-regression

**DOI:** 10.1093/ajcn/nqz110

**Published:** 2019-06-07

**Authors:** Aureliane Chantal Stania Pierret, James Thomas Wilkinson, Matthias Zilbauer, Jake Peter Mann

**Affiliations:** 1 Department of Pharmacology, University of Cambridge, Cambridge, UK; 2 Clinical School of Medicine, University of Cambridge, Cambridge, UK; 3 Department of Paediatrics, University of Cambridge, Cambridge, UK; 4 Metabolic Research Laboratories—Institute of Metabolic Science, University of Cambridge, Cambridge, UK; 5 MRC Epidemiology Unit, Institute of Metabolic Science, University of Cambridge, Cambridge, UK

**Keywords:** intestinal failure, pediatric, parenteral nutrition, gastroenterology, liver failure, sepsis, intestinal transplant, meta-analysis

## Abstract

**Background:**

Intestinal failure (IF) is associated with significant morbidity and mortality, yet specific parameters that determine medium- and long-term outcomes remain ill defined.

**Objective:**

The aim of this study was to determine the long-term outcomes in childhood IF and identify patient characteristics associated with clinical endpoints.

**Design:**

MEDLINE and EMBASE were searched for cohorts of >10 pediatric-onset IF patients with >12 mo follow-up. Random-effects meta-analysis and meta-regression weighted by follow-up duration were used to calculate clinical outcome rates and patient factors associated with outcomes. Primary outcome was mortality rate; secondary outcomes included neurodevelopmental status, transplantation, IF-associated liver disease (IFALD), enteral autonomy, and sepsis.

**Results:**

In total, 175 cohorts (9318 patients and 34,549 y follow-up) were included in the meta-analysis. Overall mortality was 5.2% per y (95% CI: 4.3, 6.0) and was associated with sepsis and IFALD on meta-regression. Mortality rate improved with time from 5.9% per y pre-2000 to 4.5% per y post-2005. Sepsis rate was also predictive of IFALD and liver failure. Enteral autonomy was associated with small bowel length but not presence of ileo-cecal valve. There was a relative lack of data on neurodevelopmental outcomes.

**Conclusions:**

Sepsis is the primary modifiable factor associated with mortality and liver failure, whereas enteral autonomy correlates with small-bowel length. No clear parameters have been identified that accurately predict neurodevelopmental outcomes, and hence further research is needed. Together, our findings are helpful for parental counseling and resource planning, and support targeting reduction in sepsis.

## Introduction

Intestinal failure (IF) can be defined as a reduction in gut function below the minimum necessary for the absorption of macronutrients and/or water and electrolytes, such that intravenous supplementation is required to maintain health and/or growth, usually as parenteral nutrition (PN) for more than 2 mo (1–3). The etiology of IF includes a range of conditions, such as short bowel syndrome (SBS) and motility disorders (4). SBS is frequently the result of extensive intestinal resection for congenital (e.g., gastroschisis) or acquired [e.g., necrotizing enterocolitis (NEC) (5)] disorders.

Despite the wide range of underlying pathology leading to IF and the requirement of complex medical management approaches, there are a number of common challenges which have been identified as being linked to medium- and long-term outcomes, for example catheter-related sepsis (4). Furthermore, a number of novel treatment strategies have been developed since the introduction of PN half a century ago (6, 7). Among these are the establishment of specialist intestinal rehabilitation centers (8), use of omega-3-polyunsaturated lipid in PN (9), and refinement of intestinal lengthening surgical techniques (10). Individual centers have reported improvements in mortality (11) but this has yet to be systematically established.

The primary aim of managing IF in childhood is to achieve enteral autonomy by optimizing intestinal function, leading to complete independence from PN. However, in a large proportion of patients this cannot be achieved and hence avoiding complications [such as IF-associated liver disease (IFALD)] while providing a high quality of life for patients becomes the main priority.

Indeed, the majority of long-term adverse clinical outcomes in IF patients have been linked to prolonged requirement for PN, notably the development of IFALD and catheter-related bloodstream infections (4). A number of other clinical [e.g., NEC etiology (12), small bowel length, presence of ileocecal valve (ICV)] and biochemical [e.g., plasma citrulline (13)] factors have also been associated with outcomes, in particular enteral autonomy. Although some of these associations have been robustly replicated (e.g., small bowel length and enteral autonomy), others have been reported only in isolated cohorts (e.g., PN duration and development of liver failure) (14, 15).

To date, the majority of studies reporting outcomes have been single center and retrospective, though some larger multicenter studies have been conducted (3, 16–19). However, in order to identify common parameters that are closely linked with long-term outcomes in children with IF, the analysis of larger datasets is required. Results of such analyses have the potential to provide the basis for the development of treatment guidelines, optimize clinical practice, and ultimately lead to improved outcomes.

In this study, we conducted a systematic review, meta-analysis, and meta-regression of prospective and retrospective cohort studies reporting long-term clinical outcomes of childhood-onset IF. We specifically aimed to establish the incidence of key clinical outcome measures and identify predictors that can be used to optimize treatment approaches in the future.

## Subjects and Methods

### Review protocol and eligibility criteria

The study protocol was prospectively registered on the PROSPERO systematic review database (CRD42017072185) and was conducted in accordance with guidelines (20, 21). Inclusion criteria were as follows: >10 patients with childhood-onset (<18 y) intestinal failure, >12 mo follow-up, and data on at least 2 clinical endpoints (mortality, intestinal transplant, IFALD, enteral autonomy, sepsis, and neurodevelopment). Included study types were as follows: randomized controlled trials (RCTs), cohort studies, case control studies, case series, systematic reviews, and meta-analyses. Participants and cohorts were defined as having intestinal failure when there was a requirement of parental nutrition (PN) for >60 consecutive days (22), or, if these data were not available, when defined by the authors as “intestinal failure.”

Studies were excluded if it was not possible to separate data from adults from those from children, there was no detail on minimum duration of PN, or it was not possible to separate outcomes for IF from those for non-IF patients. Narrative reviews, editorials, opinions, and animal research studies were also excluded.

### Information sources and search strategy

MEDLINE and EMBASE (including conference abstracts from ESPGHAN, NASPGHAN, ESPEN, and ASPEN annual meetings) were searched, with the search completed on 17 March, 2019. Terms relating to IF were combined with key terms for age and clinical outcomes (e.g., IFALD, transplant). The full search strategy can be found in the **Supplemental Methods**. In addition, the reference lists from all relevant systematic reviews and meta-analyses were reviewed. No time period for publication date was specified.

### Study selection and data collection

Identified studies underwent screening followed by full text review. At screening, abstracts and titles were reviewed to exclude irrelevant studies. The full texts were then assessed for inclusion/exclusion criteria. Two reviewers (ACSP and JTW) independently performed screening and full text review, with discrepancies settled by discussion with JPM.

Two authors independently extracted data from studies. Where individual studies described multiple cohorts, data were extracted for each cohort separately. To reduce the risk of duplication of patients in the meta-analysis, studies were selected for inclusion in meta-analysis and meta-regression if their reported patient outcomes did not overlap with those of other reported cohorts. The center(s) from which patients were recruited and the years of inclusion were also compared such that only nonoverlapping cohorts were included in the meta-analysis.

Data extracted included the following: study details (e.g., number of patients in cohort, location of study); patient baseline characteristics (e.g., principle etiology of IF, gestational age, sex); interventions (medical, surgical, nutritional); details of follow-up (duration, loss to follow-up); and clinical outcomes [e.g., mortality, IFALD, liver failure, catheter-related bloodstream infections (CRBSIs) per 1000 days of catheter use, enteral autonomy, number of central venous catheters (CVCs) used, venous thrombosis, height and weight at the start and end of follow-up, and neurodevelopmental outcomes]. All available data were included in analyses, even if not all items were reported in each study. Missing data points resulted in studies being censored from only those analyses. The number of patients included in each analysis is reported in the results. Unless otherwise stated, liver failure was defined as the number of patients listed for liver transplant, who underwent liver transplant, or who died of liver-related causes. Sixty-eight authors were contacted for missing data, with 25 responding.

Data on reported associations (or predictors) of clinical outcomes were extracted from each included study. It was recorded whether associations had been established using a univariate or multivariate analysis.

### Study heterogeneity

Studies were found to fall into 5 groups as follows: *1*) those reporting only patients on home parenteral nutrition (HPN); *2*) children who had been referred for intestinal transplant (ITx); *3*) children with established IFALD; *4*) patients undergoing intestinal lengthening surgical procedures; and *5*) other (predominantly neonatal) cohorts with IF. These 5 groups were used for descriptive purposes only, whereas meta-analysis was performed by combining all studies in a global analysis.

### Quality assessment and risk of bias (in individual studies)

Two reviewers (JTW and JPM) independently assessed risk of bias in each study by applying the Cochrane Risk of Bias in Cohort Studies tool (23).

### Statistical analysis

Primary outcome was mortality rate (percentage mortality per year of follow-up); secondary outcomes included neurodevelopmental status, transplantation, IFALD, enteral autonomy, and sepsis.

Raw data from each study were used to generate summary measures. Means with 95% CIs were calculated for baseline characteristics (age, sex, IF etiology, gestational age, birth weight, small bowel length, number of CVCs), weighted by the number of years of patient follow-up.

For clinical outcomes (total mortality, total transplants, enteral autonomy, IFALD, and liver failure), crude proportions were calculated and then meta-analysis was performed to calculate the proportion affected per year of follow-up. IFALD cohorts (with 100% IFALD prevalence) were excluded from the assessment of IFALD prevalence. Studies were weighted by person-years follow-up with double arcsine transformation (24). Random effects were used throughout with 95% CIs. Heterogeneity was calculated according to Cochrane's statistic and its related metric *I*^2^. Publication bias was assessed using funnel plots, and the Egger test was used when >10 studies were included.

Random-effects meta-regression was performed to assess the effects of baseline cohort characteristics on each clinical outcome.

Statistical analysis was performed using STATA v14.0 for Windows (StataCorp, 2015. Stata Statistical Software: Release 14).

## Results

### Study selection and characteristics

In total, 2203 articles were identified, of which 201 studies were included in the systematic review that contained 220 cohorts (**Table 1**, and **Supplemental Tables 1** and **2**). After exclusion of overlapping cohorts, 175/220 cohorts (9318 patients and 34,549 patient-years follow-up) were included in the meta-analysis (**Supplemental Figure 1**). The majority of cohorts (189/220, 86%) were retrospective and most studies (161/201, 80%) were low or very-low risk of bias (**Supplemental Figure 2**).

**TABLE 1:** Clinical outcomes (from meta-analysis) for patients with IF^1^

	Overall (*n* = 9318)
Mean follow-up, mo	44.5
Total mortality, % (*n*/total)	16.5 (1377/8340)
Weighted, %/y (95% CI)	5.2 (4.3, 6.0)
IFALD, % (*n*/total)	43.5 (2065/4746)
Weighted, %/y (95% CI)	17 (14, 20)
Liver failure, % (*n*/total)	13.9 (884/6338)
Weighted, %/y (95% CI)	4.3 (3.4, 5.3)
Transplant, % (*n*/total)	8.9 (731/8172)
Weighted, %/y (95% CI)	1.2 (0.7, 1.7)
Enteral autonomy, % (*n*/total)	54.1 (3653/6747)
Weighted, %/y (95% CI)	20 (18, 23)
Thrombosis, % (*n*/total)	25.7 (328/1277)
Weighted, %/y (95% CI)	7.5 (4.3, 11.3)
CRBSI, per 1000 catheter d (95% CI)	3.3 (3.2, 3.4)

1 CRBSI, catheter-related bloodstream infection; IF, intestinal failure. IFALD, IF-associated liver disease. Meta-analysis of clinical outcomes in IF for all included studies. Data were analyzed by crude proportions (*n*/total) and random effects meta-analysis for event rates (%/y).

### Mortality

The overall mortality of childhood IF was 5.2% per y (95% CI: 4.3, 6.0), or 16.5% (1377/8340) over a mean 44.5-mo follow-up (**Supplemental Figure 3**). The main causes of death were liver disease (23%), sepsis (18%), and posttransplant status (12%), with cause of death unknown in 35% of cases.

**FIGURE 1:**
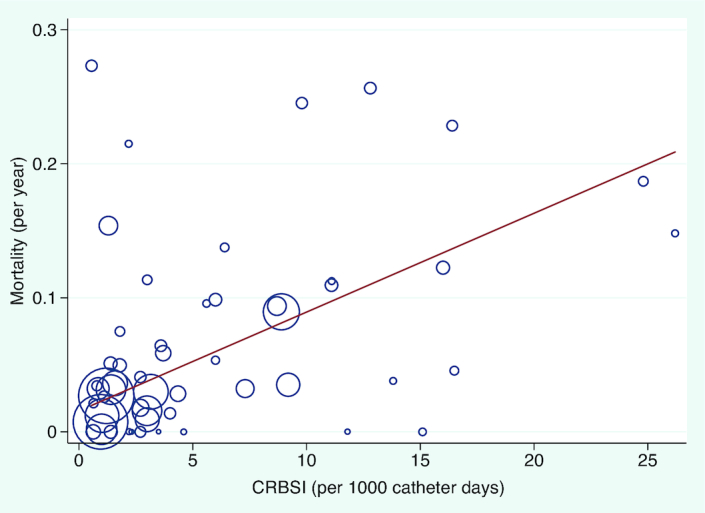
Meta-regression between rate of CRBSI per 1000 days and mortality per year. Each study (*n* = 53) is represented by a circle and size demonstrates weighting of each study, where larger circles indicate more patient-years of follow-up. The line of best fit shows the change in mortality rate for each incidence of CRBSI per 1000 days (β: 0.007; 95% CI: 0.003, 0.01), *P* value = 0.003. CRBSI, catheter-related bloodstream infection.

Rates of CRBSI (**Figure 1**) and IFALD (**Supplemental Figure 4**) were the 2 determining factors associated with mortality (**Supplemental Table 3**). Duration of PN, small bowel length, and etiology were not predictive of mortality, despite previously reported associations (**Supplemental Figure 5**).

**FIGURE 2:**
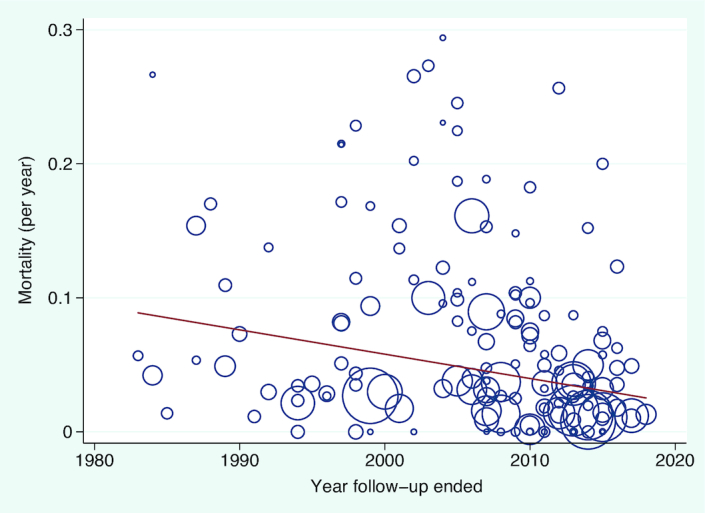
Meta-regression of the year follow-up ended and all-cause mortality. Each study (*n* = 149) is represented by a circle and size demonstrates weighting of each study, where larger circles indicate more patient-years of follow-up. The line of best fit shows the change in mortality rate for each year (β: −0.002; 95% CI: −0.003, −0.0003), *P* value = 0.018.

There was a trend of improving mortality with time: the year follow-up ended (i.e., more recent cohorts) was associated with improved mortality on meta-regression (*P* = 0.02, **Figure 2** and **Supplemental Tables** 3 and **4**).

### Enteral autonomy

**FIGURE 3:**
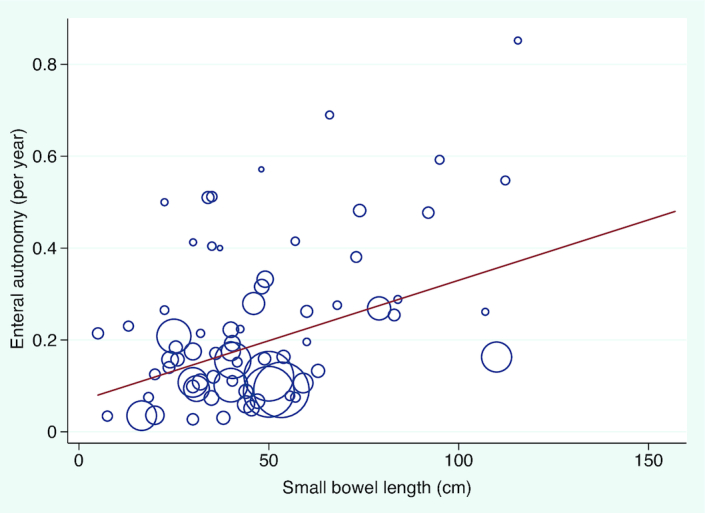
Meta-regression between small bowel length and the proportion of patients reaching enteral autonomy. Each study (*n* = 71) is represented by a circle and size demonstrates weighting of each study, where larger circles indicate more patient-years of follow-up. The line of best fit shows the change in enteral autonomy rate for centimeter increase in small bowel length (β: 0.002; 95% CI: 0.001, 0.004), *P* value = 2.5 × 10^−4^.

Enteral autonomy was achieved in 54% (3653/6747) of children at a rate of 20.1% per y (95% CI: 17.6, 22.6, **Supplemental Figure 6**). Enteral autonomy was strongly correlated with small bowel length (**Figure 3** and **Supplemental Table 5**) and NEC as an etiology was associated with higher rates of enteral autonomy on meta-regression (**Supplemental Figure 7**), which has been reported previously (**Supplemental Figure 8**). However, presence of the ileocecal valve was not associated with enteral autonomy. The 7 cohorts with “ultra-short bowel syndrome” had lower rates of enteral autonomy but no other differences in clinical outcomes (**Supplemental Table 6**).

Similar to all-cause mortality, there was evidence of improved rates of enteral autonomy in more recent cohorts (Supplemental Table 4) and when assessed by meta-regression (*P* = 0.03, Supplemental Table 5). Funnel plots for enteral autonomy and mortality were asymmetrical (**Supplemental Figures 9** and **10**).

### Liver disease

**FIGURE 4:**
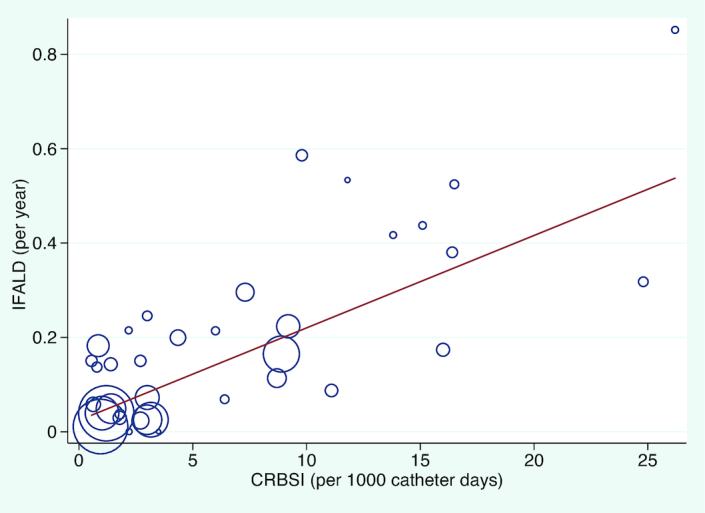
Meta-regression between the rate of CRBSI per 1000 catheter days and development of IFALD. Each study (*n* = 37) is represented by a circle and size demonstrates weighting of each study, where larger circles indicate more patient-years follow-up. The line of best fit shows the change in IFALD rate for each incidence of CRBSI per 1000 days (β: 0.02; 95% CI: 0.01, 0.03), *P* value = 1.7 × 10^−7^. CRBSI, catheter-related bloodstream infection; IFALD, intestinal failure–associated liver disease.

IFALD affected 43.5% (2065/4746) of patients and was associated with sepsis and gestational age, but not with total duration of PN or bowel length. Meta-regression demonstrated strong positive correlations between IFALD and CRBSI (*P* = 1.7 × 10^−7^, **Figure 4** and **Supplemental Table 7**), as well as IFALD and NEC (*P* = 1.2 × 10^−5^, Supplemental Table 7). IFALD was negatively correlated with gestational age (*P* = 5.5 × 10^−4^, Supplemental Table 7 and **Supplemental Figure 11**). Several studies had previously reported that total (or mean) durations of PN and bowel length were independent predictors of IFALD (**Supplemental Figure 12**), although we found no evidence for this.

### Neurodevelopment and other outcomes

Neurodevelopmental outcomes were described in 13/220 (6%) cohorts but were not suitable for meta-analysis. Normal development was reported in 29–100% of children; 80–90% of children were in mainstream schools. Some studies reported that children with moderate–severe developmental delay also had other comorbidities (e.g., extreme prematurity, cerebral abnormalities). One study that performed structured developmental assessments found both cognitive and motor impairment in >50% at 2 y (25).

Significant increases in both height and weight were achieved during the follow-up period (**Supplemental Figure 13**), although children remained below average and catch-up was less effective for height [*z* score = −1.0 (95% CI: −1.1, −1.03)] than weight [*z* score = −0.88 (95% CI: −0.89, −0.86)].

Despite recent reports of lower sepsis rates (26), we were unable to find a difference between early and late cohorts.

### Reporting and definitions

There was high variation in the reporting of baseline patient characteristics (**Supplemental Table 8**). Twenty-eight definitions of “intestinal failure” and 9 definitions of IFALD were used by included studies (**Supplemental Table 9**).

## Discussion

In this meta-analysis we have generated accurate rates for clinical outcomes and demonstrated that sepsis is the most important modifiable risk factor for morbidity and mortality. These results deepen our understanding of the condition in addition to providing useful baseline data for guideline development and counseling of parents.

We found a 5% annual mortality rate for children with IF, which was heavily driven by sepsis and liver disease, as had been found in individual cohorts (3, 27). Further analysis demonstrated that CRBSI was also strongly associated with IFALD, along with prematurity and NEC. This is consistent with the hypothesis that recurrent septic inflammation causes hepatic immune activation, which triggers progressive fibrosis, and that this is particularly harmful in the context of an immature liver (28–31).

Prevention of sepsis has long been recognized as a key outcome in the management of IF. Ethanol locks have also been shown to reduce the CRBSI rate by 81% (32), and implementation of a multidisciplinary intestinal rehabilitation program can reduce sepsis and mortality rates (33). However, we were unable to demonstrate improvement in sepsis rates in this analysis, which may reflect the broad nature of the review, including cohorts from small and large centers. Our data suggest that widespread adoption of established beneficial interventions could limit the morbidity and mortality associated with IFALD.

These results challenge the notion that duration of PN, small bowel length, or presence of ICV are causative for IFALD. Although long-term PN is necessary for the development of IFALD, it does not seem to be sufficient. Lauriti et al. had found PN duration to correlate with incidence of cholestasis; however, this study compared shorter PN durations, including <30 days (14). Cumulatively, these data suggest that the factors predictive of IFALD are distinct from those associated with achieving enteral autonomy.

Consistent with evidence from individual centers of improving mortality (11), we found a trend of reducing mortality on meta-regression. We report lower mortality and higher enteral autonomy than found by Squires et al. (3), which may reflect the shorter follow-up (26 mo) in their study. Improved neonatal care, development of surgical techniques, and use of fish oil–based lipid, among other interventions, are likely to have contributed to improved mortality. Similarly, the proportion of patients reaching enteral autonomy has improved with time. Enteral autonomy was strongly associated with small bowel length but not with presence of ICV. Though previous studies (15) have found the ICV to be a key variable, in this meta-regression it was not associated with any clinical outcomes. Presence of ICV is also strongly correlated with presence of the colon, which may independently be beneficial for weaning of PN (34, 35). Our study also corroborates previous findings that NEC is positively associated with weaning from PN (15, 36). This result may reflect that other etiologies of short bowel syndrome (for example, gastroschisis or atresias) are associated with poorer enteral autonomy (36). The mechanism for this is unclear but supports the concept that disease etiology impacts on the rehabilitation capacity in remaining bowel.

Our data also illustrate the overall burden of morbidity for these patients, including need for CVC replacement and frequency of venous thrombosis. Extensive venous thrombosis (with loss of central access) is an indication for intestinal transplant. However, many of these thromboses are subclinical (37), and therefore it is not yet established whether active surveillance or prophylaxis would be of benefit (38).

### Limitations of the study

Neurodevelopment is a key outcome for (often expremature) children with any chronic disease. So et al. have recently reported 2 structured assessments of neurodevelopment that reveal significant deficits even into childhood (25, 39). Overall, there was limited reporting of developmental outcomes and insufficient evidence to draw firm conclusions, highlighting the need for further work in this area.

As a cohort meta-analysis, it was not possible to follow individuals through their disease course. If individual patient-level data had been available, it would have facilitated more detailed analysis of patients’ disease course by disease etiology. A further risk of cohort meta-analyses is the potential for double-counting of individual patients by their inclusion in 2 studies. We attempted to limit this by excluding data from overlapping cohorts at each center but were unable to verify whether any patients were duplicated in our analyses.

This meta-analysis and meta-regression was also limited by underreporting of baseline characteristics, such as ethnicity and sex, and a lack of individual patient-level data, which reduced the power for exploring associations. There were also limited data from outside North America and Europe.

Finally, there was evidence of bias on funnel plots. This may be due to: *1*) analysis of mortality ratios, which limits the lower bound as zero; *2*) study heterogeneity; or *3*) publication bias, as similar bias was observed for enteral autonomy.

### Conclusion

Sepsis and IFALD are the 2 key variables most strongly associated with mortality in pediatric IF, although overall mortality has improved. The associations of IFALD are distinct from the factors predicting enteral autonomy. The neurodevelopmental outcome of pediatric IF is unclear and requires further investigation.

## Supplementary Material

nqz110_Supplemental_File

## Notes

JPM is supported by a Wellcome Trust Fellowship (216329/Z/19/Z).

Supplemental Methods, Supplemental Tables 1–9 and Supplemental Figures 1–13 are available from the “Supplementary data” link in the online posting of the article and from the same link in the online table of contents at https://academic.oup.com/ajcn/.

Abbreviations used: CRBSI, catheter-related bloodstream infection; CVC, central venous catheter; HPN, home parenteral nutrition; ICV, ileocecal valve; IF, intestinal failure; IFALD, intestinal failure–associated liver disease; ITx, intestinal transplantation; NEC, necrotizing enterocolitis; PN, parenteral nutrition; RCT, randomized controlled trial; SBS, short bowel syndrome.

## References

[1] Pironi L, Arends J, Baxter J, Bozzetti F, Peláez RB, Cuerda C, Forbes A, Gabe S, Gillanders L, Holst M et al. ESPEN endorsed recommendations: definition and classification of intestinal failure in adults. Clin Nutr. 2015;34:171–80. 25311444 10.1016/j.clnu.2014.08.017

[2] Wales PW, de Silva N, Kim J, Lecce L, To T, Moore A Neonatal short bowel syndrome: population-based estimates of incidence and mortality rates. J Pediatr Surg. 2004;39:690–5. 15137001 10.1016/j.jpedsurg.2004.01.036

[3] Squires RH, Duggan C, Teitelbaum DH, Wales PW, Balint J, Venick R, Rhee S, Sudan D, Mercer D, Martinez JA et al. Natural history of pediatric intestinal failure: initial report from the Pediatric Intestinal Failure Consortium. J Pediatr. 2012;161:723–8. e2. 22578586 10.1016/j.jpeds.2012.03.062 PMC3419777

[4] Duggan CP, Jaksic T Pediatric intestinal failure. N Engl J Med. 2017;377:666–75. 28813225 10.1056/NEJMra1602650

[5] Neu J, Walker W Necrotizing enterocolitis. N Engl J Med. 2011;364:255–64. 21247316 10.1056/NEJMra1005408 PMC3628622

[6] Pilling G, Cresson S Massive resection of the small intestine in the neonatal period: report of two successful cases and review of the literature. Pediatrics. 1957;19:940–8. 13431320

[7] Rickham PP Massive small intestinal resection in newborn infants. Ann R Coll Surg Engl. 1967;41:480–92. 6078483 PMC2312208

[8] Groen H, Neelis EG, Poley MJ, Olieman JF, Scheenstra R, Krabbe PFM, Dijkstra G, Rings EHHM Intestinal rehabilitation for children with intestinal failure is cost-effective: a simulation study. Am J Clin Nutr. 2017;105:417–25. 28052886 10.3945/ajcn.116.135160

[9] Hojsak I, Colomb V, Braegger C, Bronsky J, Campoy C, Domellöf M, Embleton N, Fildler MN, Hulst JM, Indrio F et al. ESPGHAN committee on nutrition position paper. Intravenous lipid emulsions and risk of hepatotoxicity in infants and children: a systematic review and meta-analysis. J Pediatr Gastroenterol Nutr. 2016;62:776–92. 26825766 10.1097/MPG.0000000000001121

[10] Frongia G, Kessler M, Weih S, Nickkholgh A, Mehrabi A, Holland-Cunz S Comparison of LILT and STEP procedures in children with short bowel syndrome: a systematic review of the literature. J Pediatr Surg. 2013;48:1794–805. 23932625 10.1016/j.jpedsurg.2013.05.018

[11] Avitzur Y, Wang JY, de Silva NT, Burghardt KM, DeAngelis M, Grant D, Ng VL, Jones N, Wales PW Impact of intestinal rehabilitation program and its innovative therapies on the outcome of intestinal transplant candidates. J Pediatr Gastroenterol Nutr. 2015;61:18–23. 25611029 10.1097/MPG.0000000000000735

[12] Sparks EA, Khan FA, Fisher JG, Fullerton BS, Hall A, Raphael BP, Duggan C, Modi BP, Jaksic T Necrotizing enterocolitis is associated with earlier achievement of enteral autonomy in children with short bowel syndrome. J Pediatr Surg. 2016;51:92–95. 26700691 10.1016/j.jpedsurg.2015.10.023 PMC4878438

[13] Fullerton BS, Sparks EA, Hall AM, Duggan C, Jaksic T, Modi BP Enteral autonomy, cirrhosis, and long term transplant-free survival in pediatric intestinal failure patients. J Pediatr Surg. 2016;51:96–100. 26561248 10.1016/j.jpedsurg.2015.10.027 PMC4713317

[14] Lauriti G, Zani A, Aufieri R, Cananzi M, Chiesa PL, Eaton S, Pierro A Incidence, prevention, and treatment of parenteral nutrition-associated cholestasis and intestinal failure-associated liver disease in infants and children: a systematic review. J Parenter Enter Nutr. 2014;38:70–85. 10.1177/0148607113496280 23894170

[15] Khan FA, Squires RH, Litman HJ, Balint J, Carter BA, Fisher JG Horslen SP, Jaksic T, Kocoshis S, Martinez JA Predictors of enteral autonomy in children with intestinal failure: A multicenter cohort study. J Pediatr. 2015;167:29–34. e1. 25917765 10.1016/j.jpeds.2015.03.040 PMC4485931

[16] Totonelli G, Tambucci R, Boscarelli A, Hermans D, Dall'Oglio L, Diamanti A, d'Aische ADB, Pakarinen M, Reding R, Morini F et al. Pediatric Intestinal Rehabilitation and Transplantation Registry: initial report from a European collaborative registry. Eur J Pediatr Surg. 2018;28:75–80. 28838002 10.1055/s-0037-1605349

[17] Guarino A, De Marco G Natural history of intestinal failure, investigated through a national network-based approach. J Pediatr Gastroenterol Nutr. 2003;37:136–41. 12883298 10.1097/00005176-200308000-00010

[18] Lao OB, Healey PJ, Perkins JD, Reyes JD, Goldin AB Outcomes in children with intestinal failure following listing for intestinal transplant. J Pediatr Surg. 2010;45:100–7.; discussion 107. 20105588 10.1016/j.jpedsurg.2009.10.019 PMC2813842

[19] Pironi L, Joly F, Forbes A, Colomb V, Lyszkowska M, Baxter J, Gabe S, Hebuterne X, Gambarara M, Cuerda C Long-term follow-up of patients on home parenteral nutrition in Europe: implications for intestinal transplantation. Gut. 2011;60:17–25. 21068130 10.1136/gut.2010.223255

[20] Stroup DF, Berlin JA, Morton SC, Olkin I, Williamson GD, Rennie D, Moher D, Becker BJ, Sipe TA, Thacker SB Meta-analysis of observational studies in epidemiology: a proposal for reporting. Meta-analysis of observational studies in epidemiology (MOOSE) group. JAMA. 2000;283:2008–12. 10789670 10.1001/jama.283.15.2008

[21] Moher D, Liberati A, Tetzlaff J, Altman DG, Group TP Preferred reporting items for systematic reviews and meta-analyses: the PRISMA statement. PLOS Med. 2009;6:e1000097. 19621072 10.1371/journal.pmed.1000097 PMC2707599

[22] Merritt RJ, Cohran V, Raphael BP, Sentongo T, Volpert D, Warner BW, Goday PS Intestinal rehabilitation programs in the management of pediatric intestinal failure and short bowel syndrome. J Pediatr Gastroenterol Nutr. 2017;65:588–96. 28837507 10.1097/MPG.0000000000001722

[23] Cochrane Bias Methods Group Tool to assess risk of bias in cohort studies. [Internet]. 2013; [cited October 28, 2018]. Available from: http://methods.cochrane.org.

[24] Barendregt JJ, Doi SA, Lee YY, Norman RE, Vos T Meta-analysis of prevalence. J Epidemiol Community Health. 2013;67:974–8. 23963506 10.1136/jech-2013-203104

[25] So S, Patterson C, Gold A, Rogers A, Belza C, de Silva N, Avitzur Y, Wales PW Neurodevelopmental outcomes of infants with intestinal failure at 12 and 26 months corrected age. Early Hum Dev. 2019;130:38–43. 30660017 10.1016/j.earlhumdev.2018.12.020

[26] Ormsby JA, Bukoye B, Lajoie D, Shermont H, Martin L, Leger K, Mahoney J, Potter-Bynoe G, Carpenter J, Ozonoff A et al. Enhanced central venous catheter bundle for pediatric parenteral-dependent intestinal failure. Am J Infect Control. 2018;46:1284–9. 29778436 10.1016/j.ajic.2018.04.209

[27] Jones BA, Hull MA, Potanos KM, Zurakowski D, Fitzgibbons SC, Ching YA, Duggan C, Jaksic T, Kim HB Report of 111 consecutive patients enrolled in the International Serial Transverse Enteroplasty (STEP) Data Registry: a retrospective observational study. J Am Coll Surg. 2013;216:438–46. 23357726 10.1016/j.jamcollsurg.2012.12.018 PMC4887155

[28] Mutanen A, Lohi J, Heikkila P, Jalanko H, Pakarinen MP Loss of ileum decreases serum fibroblast growth factor 19 in relation to liver inflammation and fibrosis in pediatric onset intestinal failure. J Hepatol. 2015;62:1391–7. 25595885 10.1016/j.jhep.2015.01.004

[29] Duro D, Mitchell PD, Kalish LA, Martin C, McCarthy M, Jaksic T, Dunn JML, Nobuhara KK, Sylverster KG et al. Risk factors for parenteral nutrition-associated liver disease following surgical therapy for necrotizing enterocolitis. J Pediatr Gastroenterol Nutr. 2011;52:595–600. 21464752 10.1097/MPG.0b013e31820e8396 PMC3444282

[30] Hermans D, Talbotec C, Lacaille F, Goulet O, Ricour C, Colomb V Early central catheter infections may contribute to hepatic fibrosis in children receiving long-term parenteral nutrition. J Pediatr Gastroenterol Nutr. 2007;44:459–63. 17414144 10.1097/MPG.0b013e318031a5c7

[31] Sharma R, Tepas JJ, Hudak ML, Mollitt DL, Wludyka PS, Teng RJ, Premachandra BR Neonatal gut barrier and multiple organ failure: role of endotoxin and proinflammatory cytokines in sepsis and necrotizing enterocolitis. J Pediatr Surg. 2007;42:454–61. 17336180 10.1016/j.jpedsurg.2006.10.038

[32] Oliveira C, Nasr A, Brindle M, Wales PW Ethanol locks to prevent catheter-related bloodstream infections in parenteral nutrition: a meta-analysis. Pediatrics. 2012;129:318–29. 22232307 10.1542/peds.2011-1602

[33] Stanger JD, Oliveira C, Blackmore C, Avitzur Y, Wales PW The impact of multi-disciplinary intestinal rehabilitation programs on the outcome of pediatric patients with intestinal failure: A systematic review and meta-analysis. J Pediatr Surg. 2013;48:983–92. 23701771 10.1016/j.jpedsurg.2013.02.070

[34] Quirós-Tejeira RE, Ament ME, Reyen L, Herzog F, Merjanian M, Olivares-Serrano N, Vargas JH Long-term parenteral nutritional support and intestinal adaptation in children with short bowel syndrome: A 25-year experience. J Pediatr. 2004;145:157–63. 15289760 10.1016/j.jpeds.2004.02.030

[35] Diamanti A, Panetta F, Gandullia P, Morini F, Noto C, Torre G, Lezo A, Goffredo B, Daniele A, Gambarara M Plasma citrulline as marker of bowel adaptation in children with short bowel syndrome. Langenbeck's Arch Surg. 2011;396:1041–6. 21630079 10.1007/s00423-011-0813-8

[36] Demehri FR, Stephens L, Herrman E, West B, Mehringer A, Arnold MA, Brown PI, Teitelbaum DH Enteral autonomy in pediatric short bowel syndrome: predictive factors one year after diagnosis. J Pediatr Surg. 2015;50:131–5. 25598109 10.1016/j.jpedsurg.2014.10.011

[37] Cunliffe RN, Bowling TE. Artificial nutrition support in intestinal failure: principles and practice of parenteral feeding. Clin Colon Rectal Surg. 2004;17:99–105. 20011254 10.1055/s-2004-828656 PMC2780048

[38] Vegting IL, Tabbers MM, Benninga MA, Wilde JC, Serlie MJ, Tas TA, Jonkers CF, van Ommen CH Prophylactic anticoagulation decreases catheter-related thrombosis and occlusion in children with home parenteral nutrition. J Parenter Enter Nutr. 2012;36:456–62. 10.1177/0148607111416482 22245761

[39] So S, Patterson C, Evans C, Wales PW Motor proficiency and generalized self-efficacy toward physical activity in children with intestinal failure. J Pediatr Gastroenterol Nutr. 2019;68:7–12. 30052565 10.1097/MPG.0000000000002107

