# Supplementary material for: Clinical outcomes in pediatric intestinal failure: a meta-analysis and meta-regression
Source: Am J Clin Nutr. 2019 Jun 7;110(2):430–6. doi: 10.1093/ajcn/nqz110 (PMC6669059; doi:10.1093/ajcn/nqz110)
Supplement: nqz110_Supplemental_File [file nqz110_supplemental_file.pdf]

Supplement

Supplemental figure 1: Preferred Reporting Items for Systematic Reviews and Meta-analyses Flow Diagram 3
Supplemental figure 2: Risk of Bias Chart for studies included in the systematic review 4
Supplemental figure 3: Meta-analysis of mortality 5
Supplemental figure 4: Meta-regression of IFALD and all-cause mortality 6
Supplemental figure 5: Previously reported associations of mortality 7
Supplemental figure 6: Meta-analysis of enteral autonomy 8
Supplemental figure 7: Meta-regression of NEC and enteral autonomy 9
Supplemental figure 8: Previously reported associations of enteral autonomy 10
Supplemental figure 9: Funnel plot of enteral autonomy 11
Supplemental figure 10: Funnel plot of mortality 12
Supplemental figure 11: Meta-regression of gestational age and IFALD 13
Supplemental figure 12: Previously reported associations of IFALD 14
Supplemental figure 13: Change in height & weight z-score 15
Supplemental figure 14: Previously reported associations of sepsis 16
Supplemental figure 15: Previously reported associations of transplantation 17
Supplemental table 1: Summary of Baseline Characteristics and Outcomes in All Patients 18
Supplemental table 2: Characteristics of Included Studies 19
Supplemental table 3: Meta-regression for heterogeneity in total mortality 53
Supplemental table 4: Comparison of clinical outcomes for cohorts separated by era (early, middle, & late) 54
Supplemental table 5: Meta-regression for heterogeneity in enteral autonomy 55
Supplemental table 6: A Sub-analysis for cohorts of ultra-Short Bowel Syndrome (SBS) against those without ultra-SBS 56
Supplemental table 7: Meta-regression for heterogeneity in IFALD 57
Supplemental table 8: Frequency of reporting of baseline characteristics in included cohorts 58
Supplemental table 9: Definitions used by cohorts included in the systematic review 59
Supplemental table 10: Summary of previously published systematic reviews and meta-analyses 62
Supplemental references 65
Supplemental methods 79

Supplemental figure 1: Preferred Reporting Items for Systematic Reviews and Meta-analyses Flow Diagram

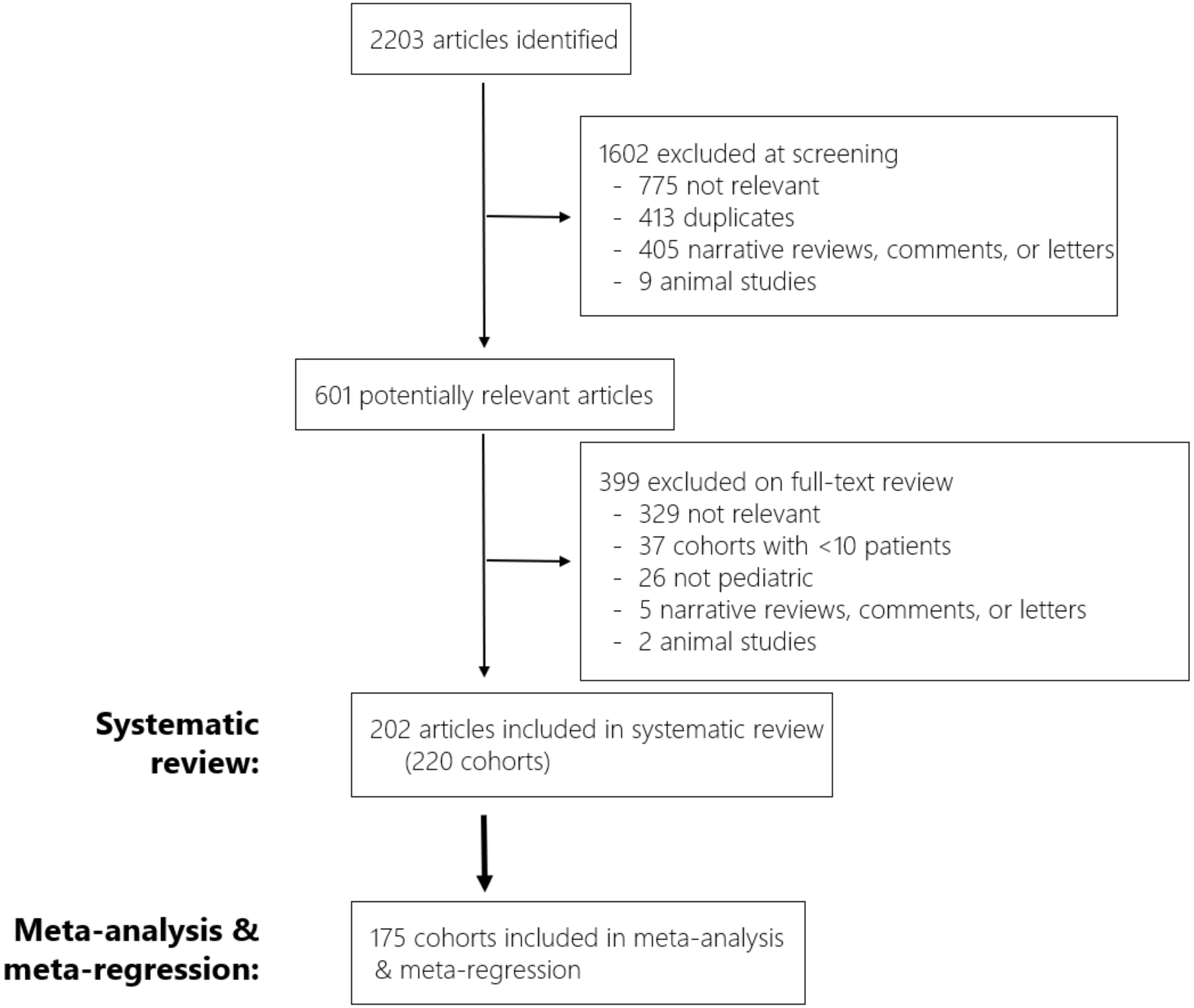

Supplemental figure 2: Risk of Bias Chart for studies included in the systematic review

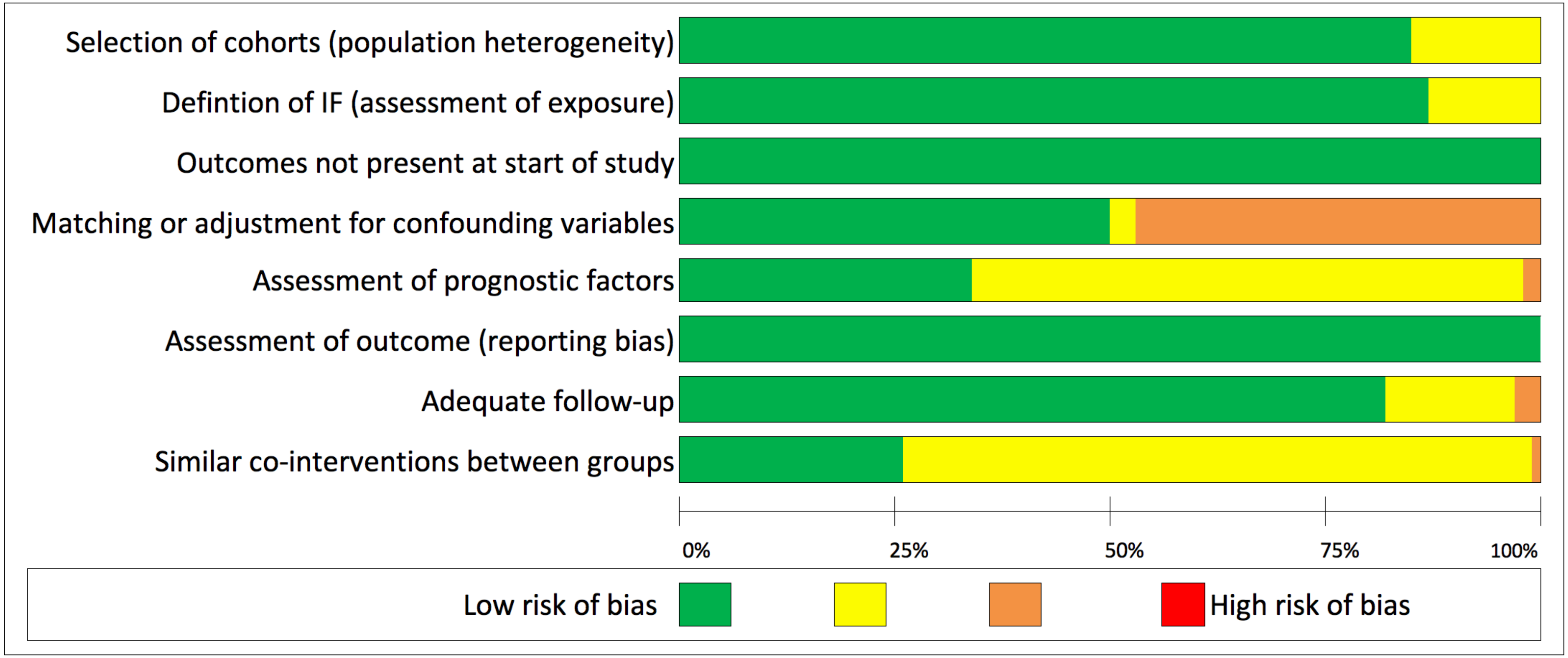

Supplemental figure 3: Meta-analysis of mortality

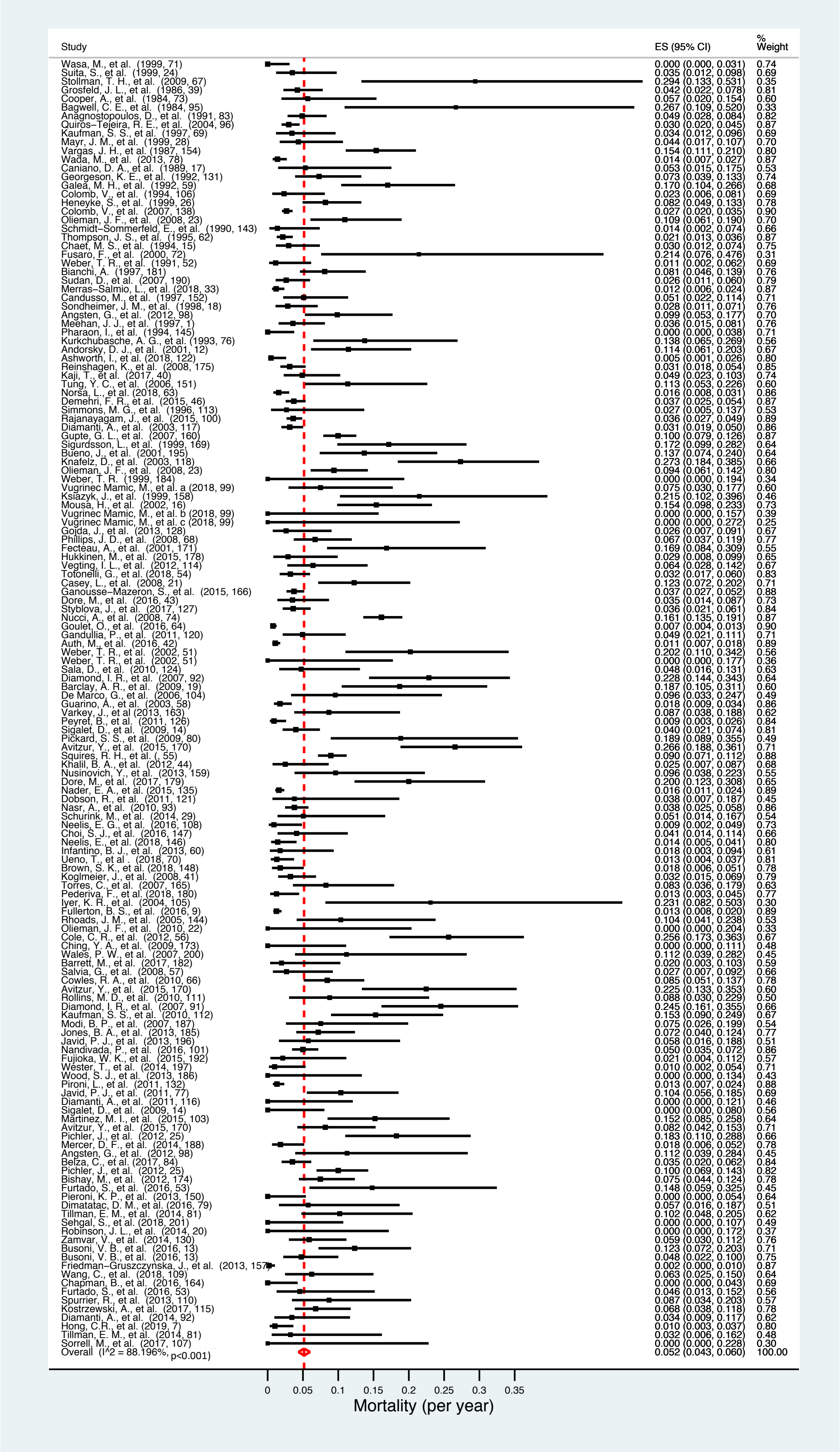

Random-effects meta-analysis by proportion for all-cause mortality per year. Each study is numbered according to Supplemental References as (Year, Reference). CI, confidence interval; ES, effect size.

Supplemental figure 4: Meta-regression of IFALD and all-cause mortality

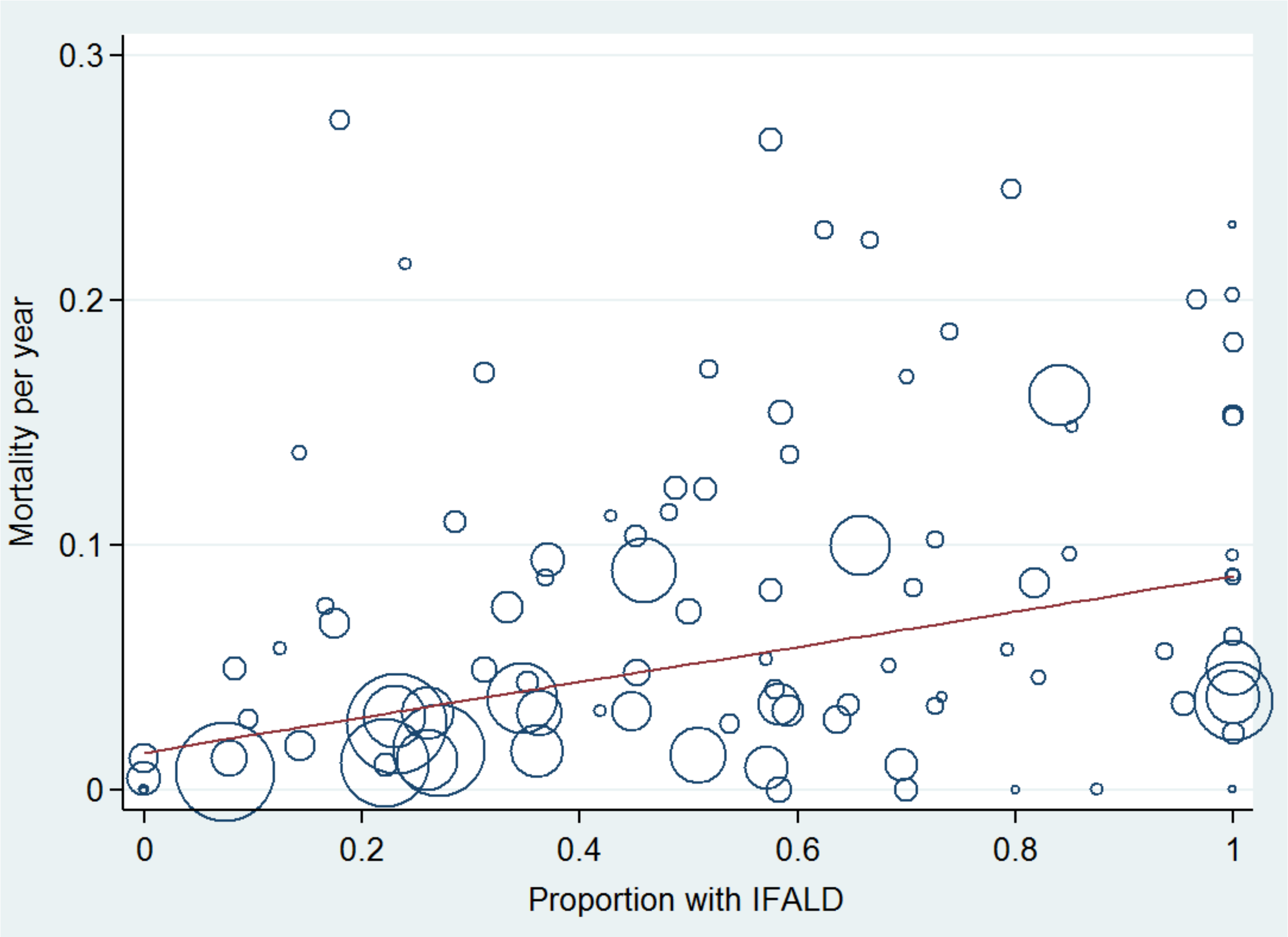

Meta-regression plot between proportion of patients with IFALD in each cohort (x-axis) and mortality per year (y-axis). Each study (n=95) is represented by a circle and size demonstrates weighting of each study, where larger circles indicate more patient-years of follow-up. The line of best fit shows the change in mortality rate for each percentage increase in patients with IFALD: β = 0.072 (95% CI 0.027,0.118), p-value = 0.002.

Supplemental figure 5: Previously reported associations of mortality

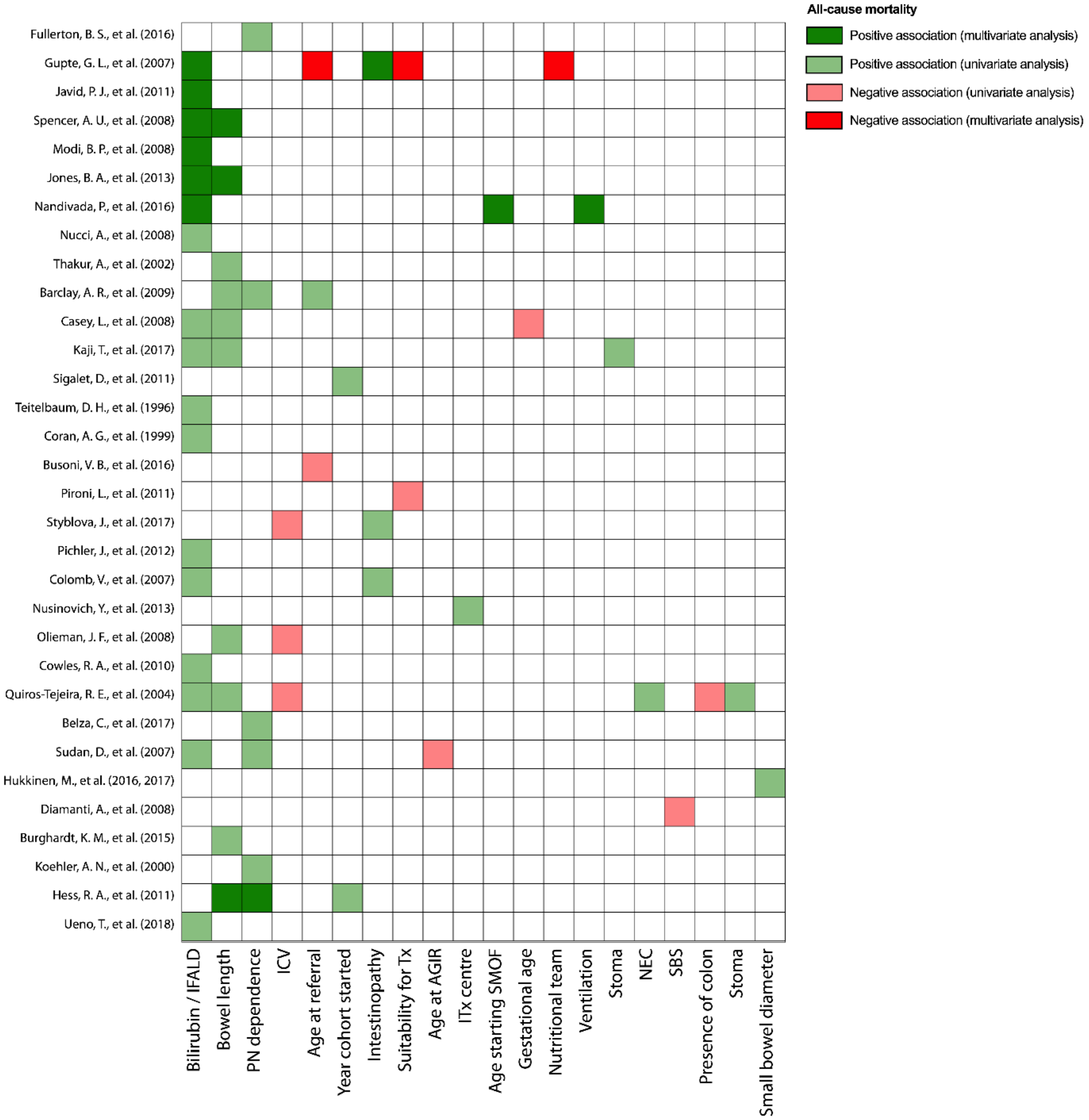

Previously reported associations (or predictors) of all-cause mortality from studies included in the systematic review.

Supplemental figure 6: Meta-analysis of enteral autonomy

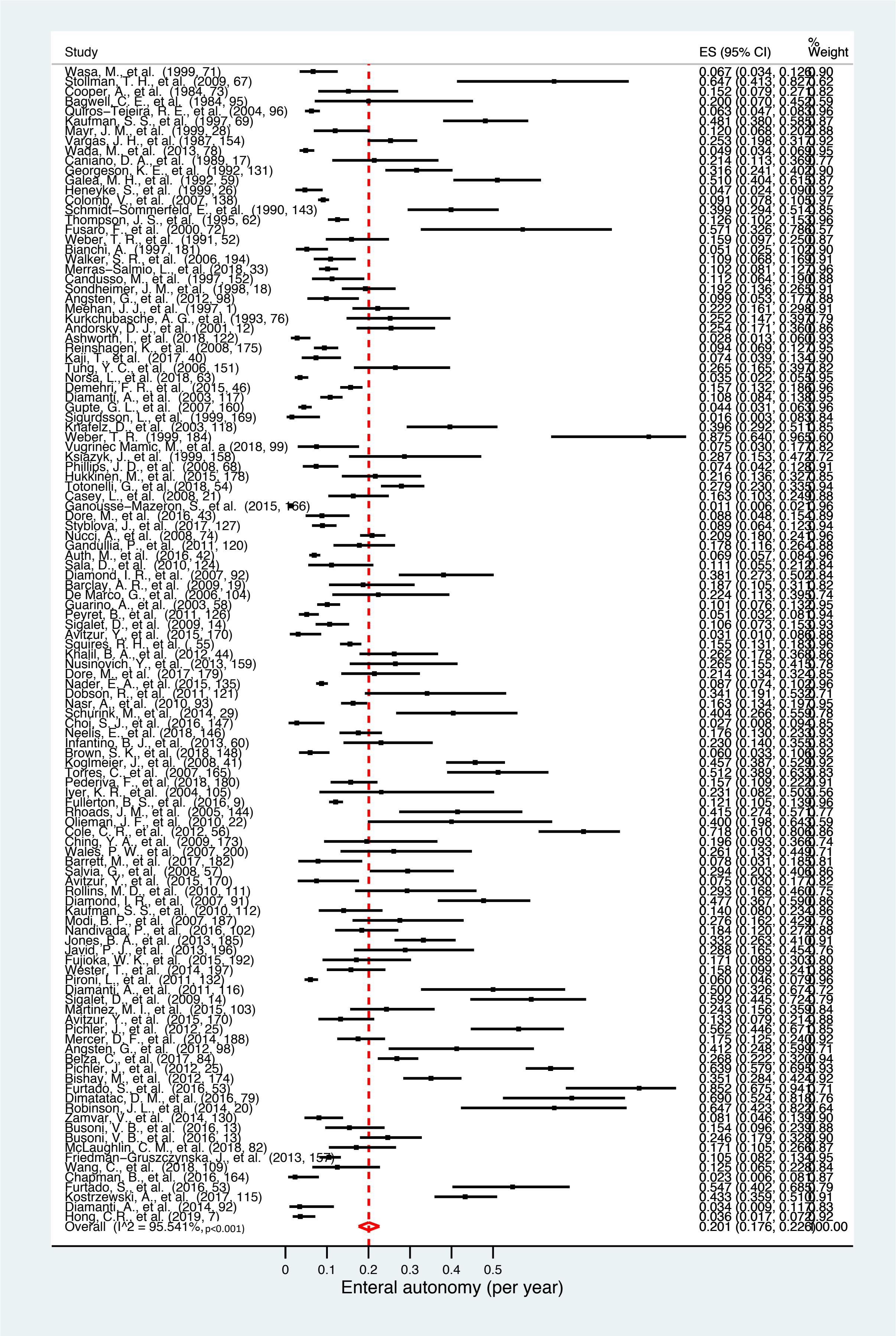

Random-effects meta-analysis by proportion for enteral autonomy per year. Each study is numbered according to Supplemental References as (Year, Reference). CI, confidence interval; ES, effect size.

Supplemental figure 7: Meta-regression of NEC and enteral autonomy

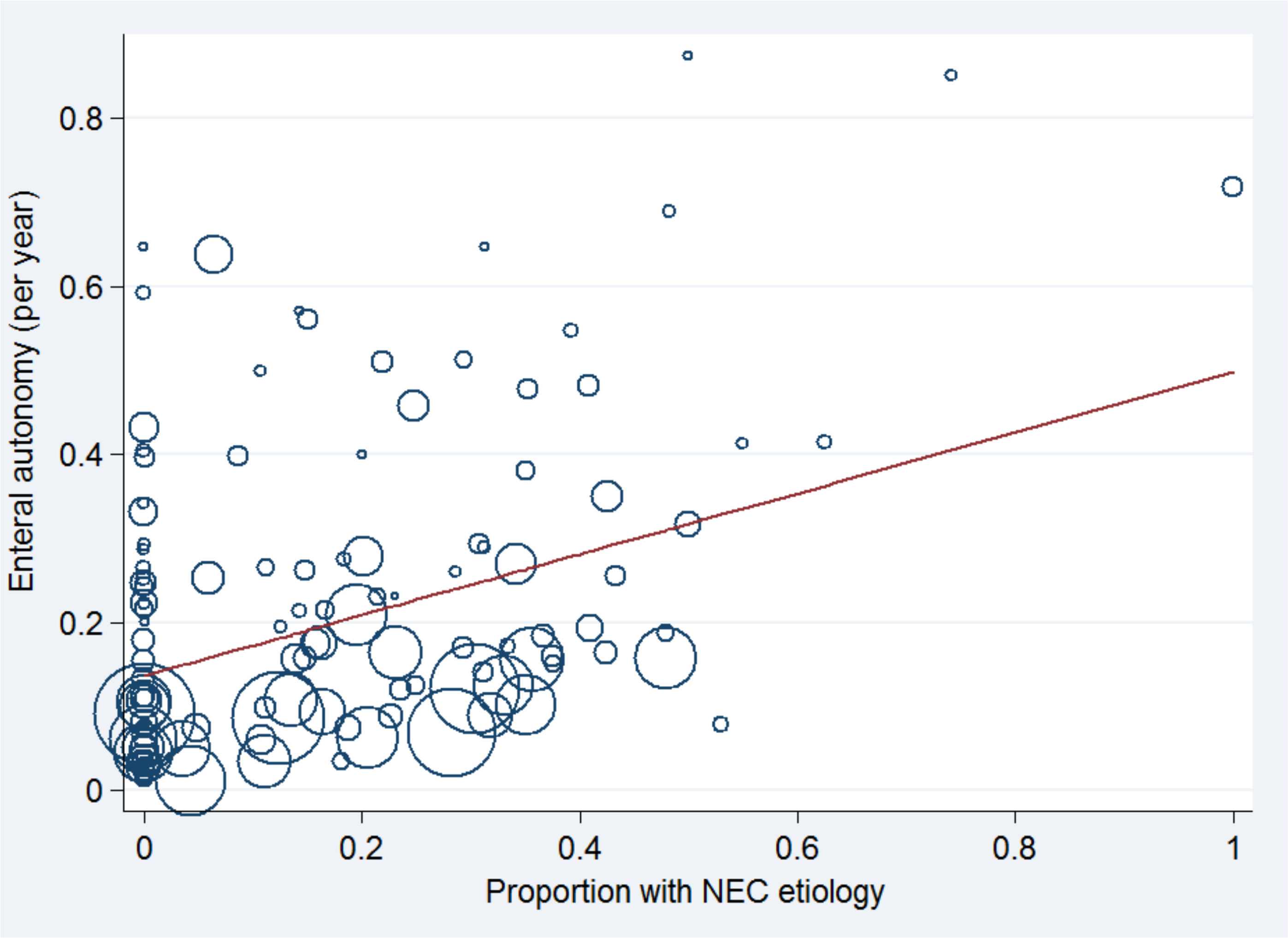

Meta-regression between proportion where necrotizing enterocolitis (NEC) is the underlying etiology and achieving enteral autonomy per year.

Supplemental figure 8: Previously reported associations of enteral autonomy

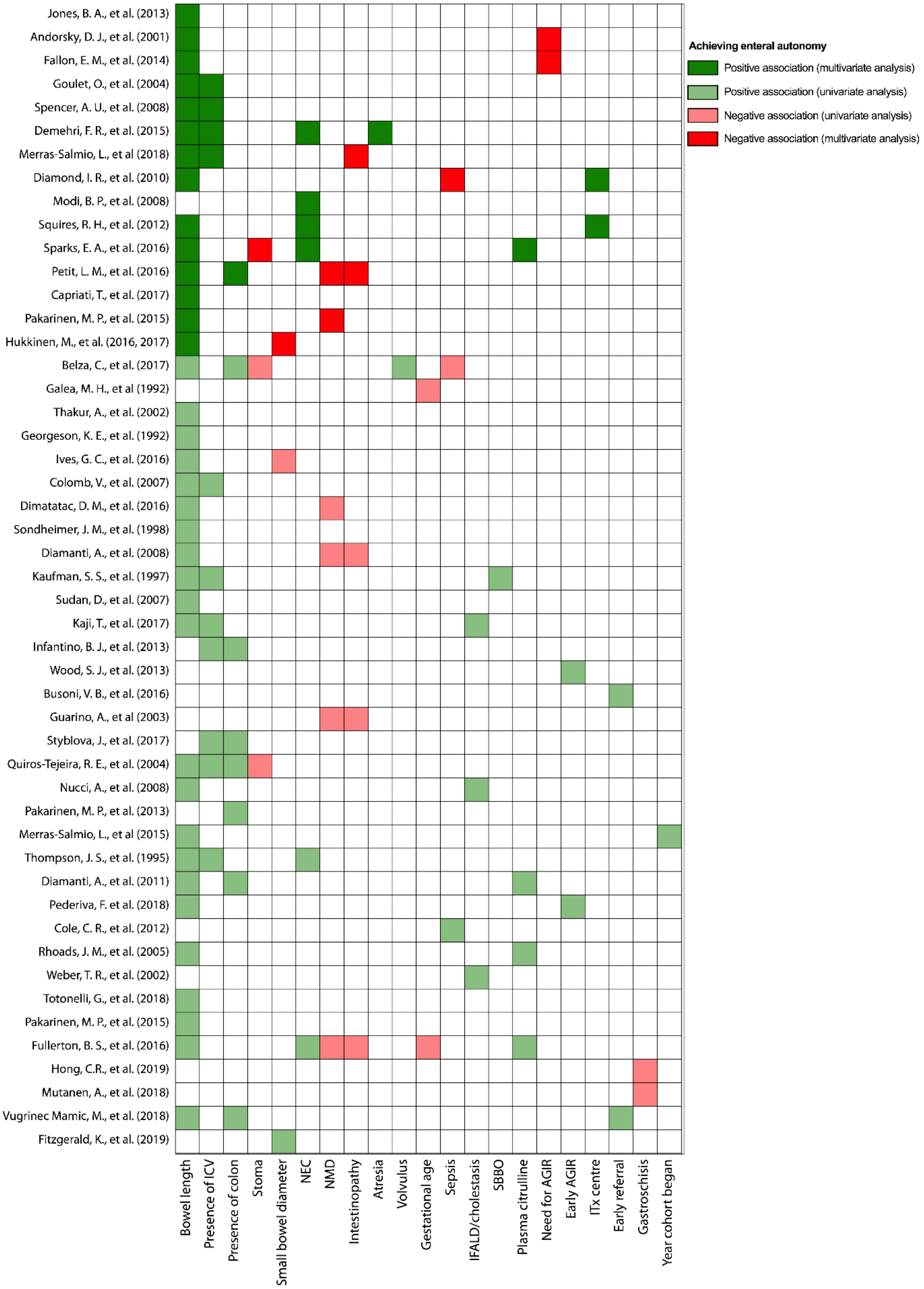

Previously reported associations (or predictors) of enteral autonomy from studies included in the systematic review.

Supplemental figure 9: Funnel plot of enteral autonomy

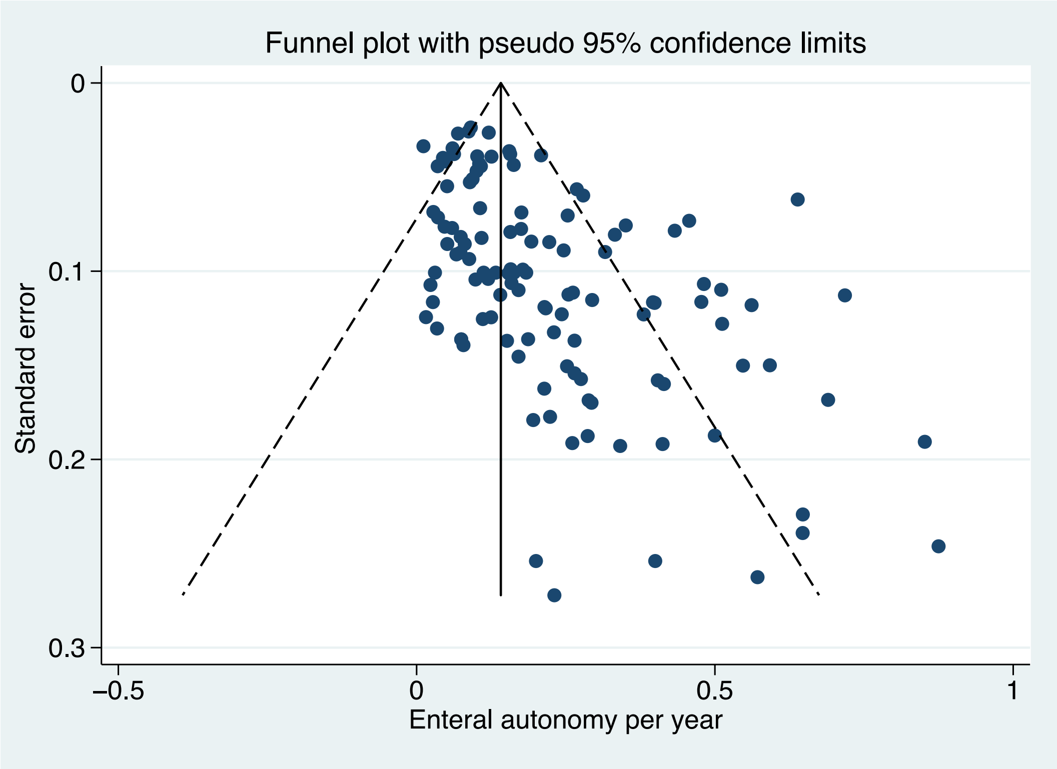

Supplemental figure 10: Funnel plot of mortality

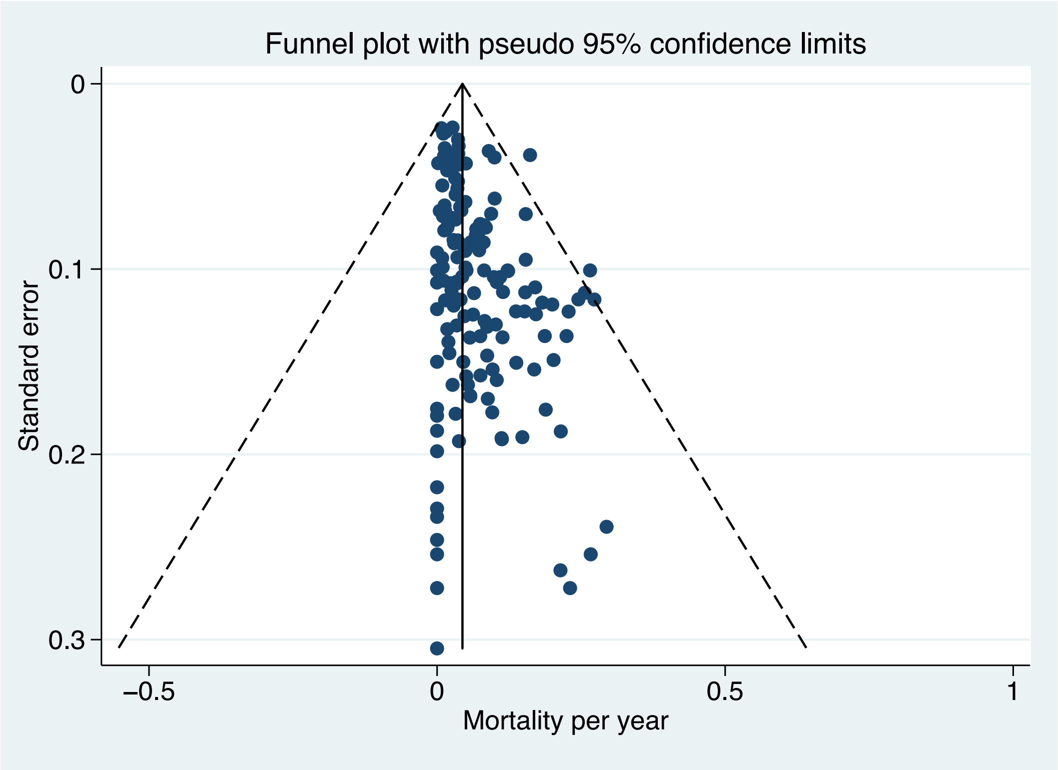

Supplemental figure 11: Meta-regression of gestational age and IFALD

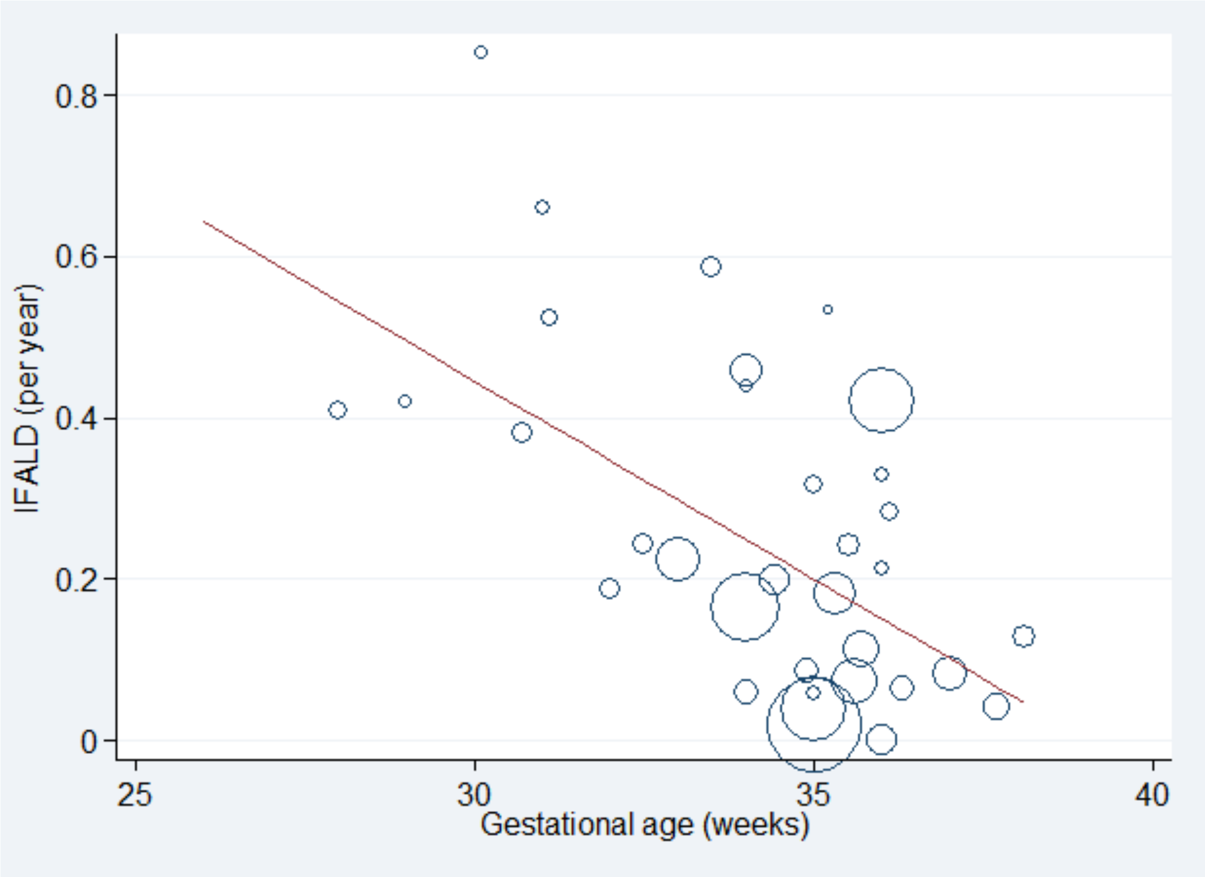

Meta-regression between gestational age and proportion affected with IFALD per year.

Supplemental figure 12: Previously reported associations of IFALD

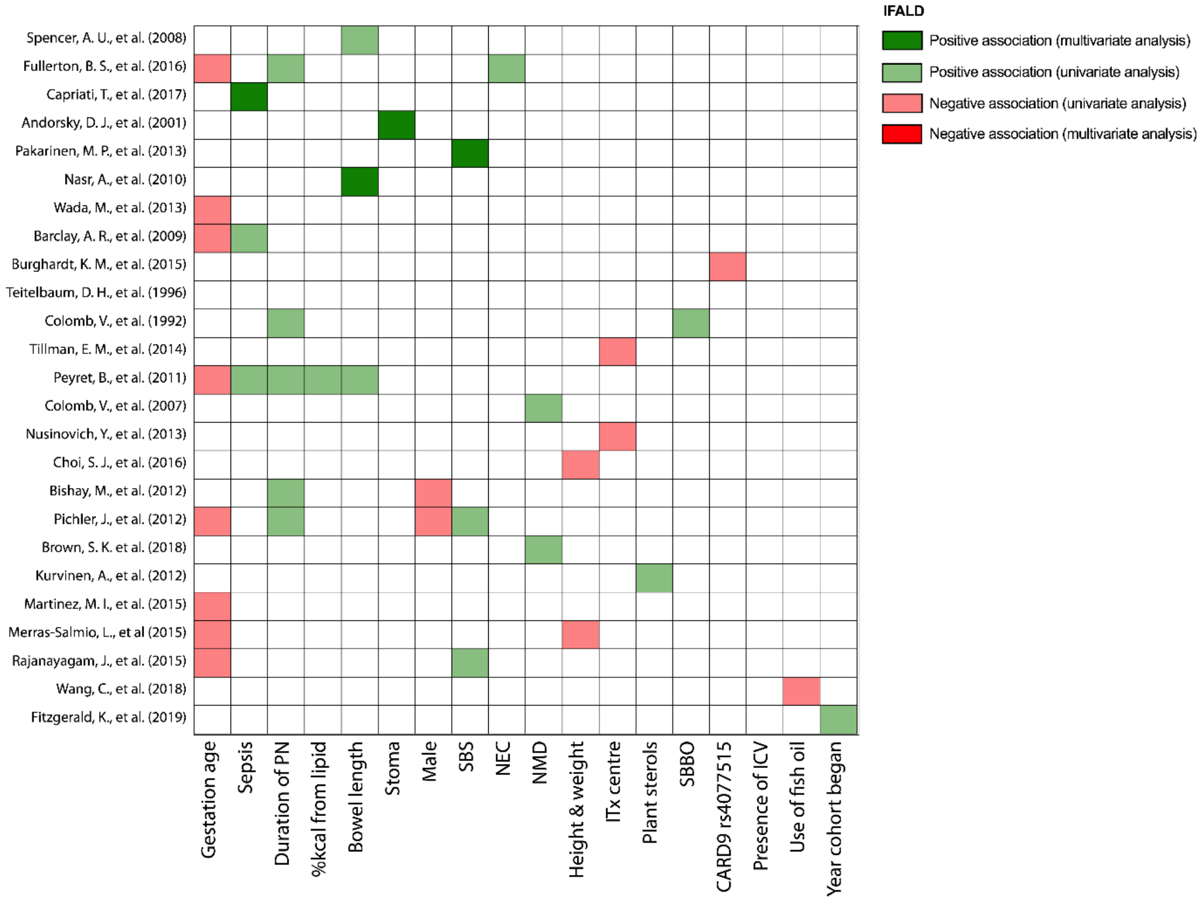

Previously reported associations (or predictors) of enteral autonomy from studies included in the systematic review.

Supplemental figure 13: Change in height & weight z-score

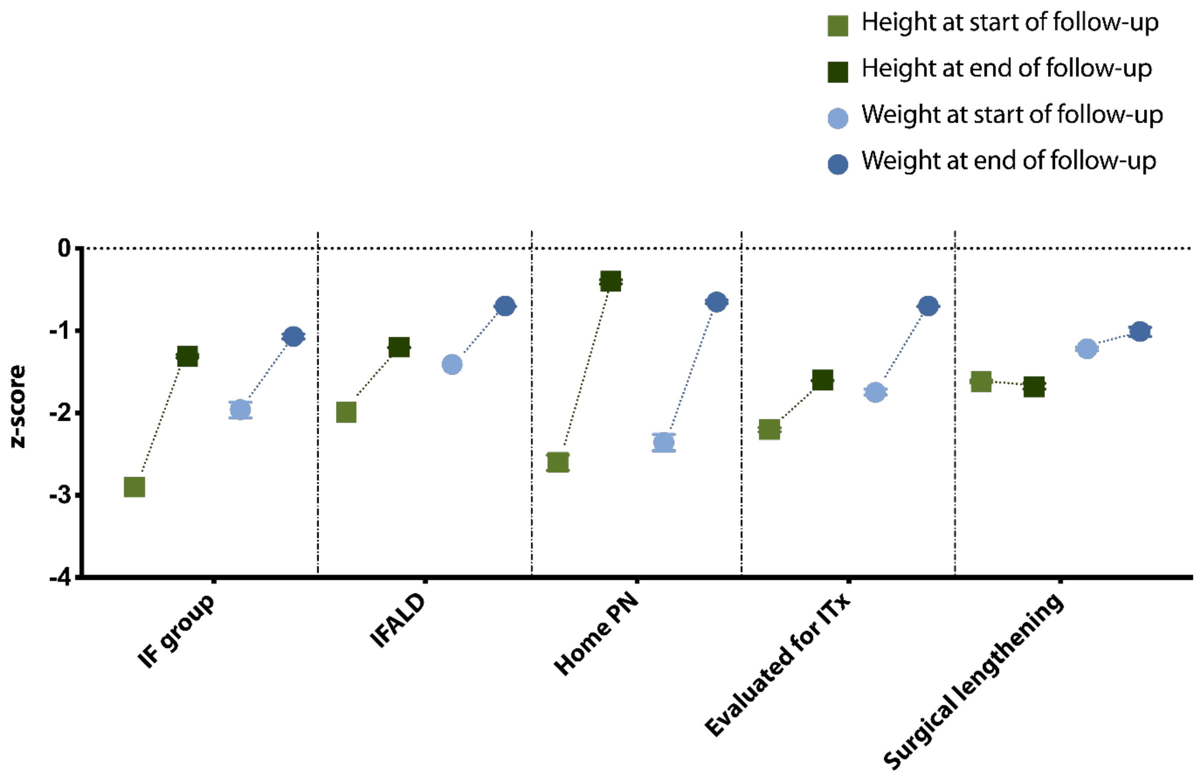

Weighted mean height and weight z-scores at the start and end of follow-up for all cohorts.

Supplemental figure 14: Previously reported associations of sepsis

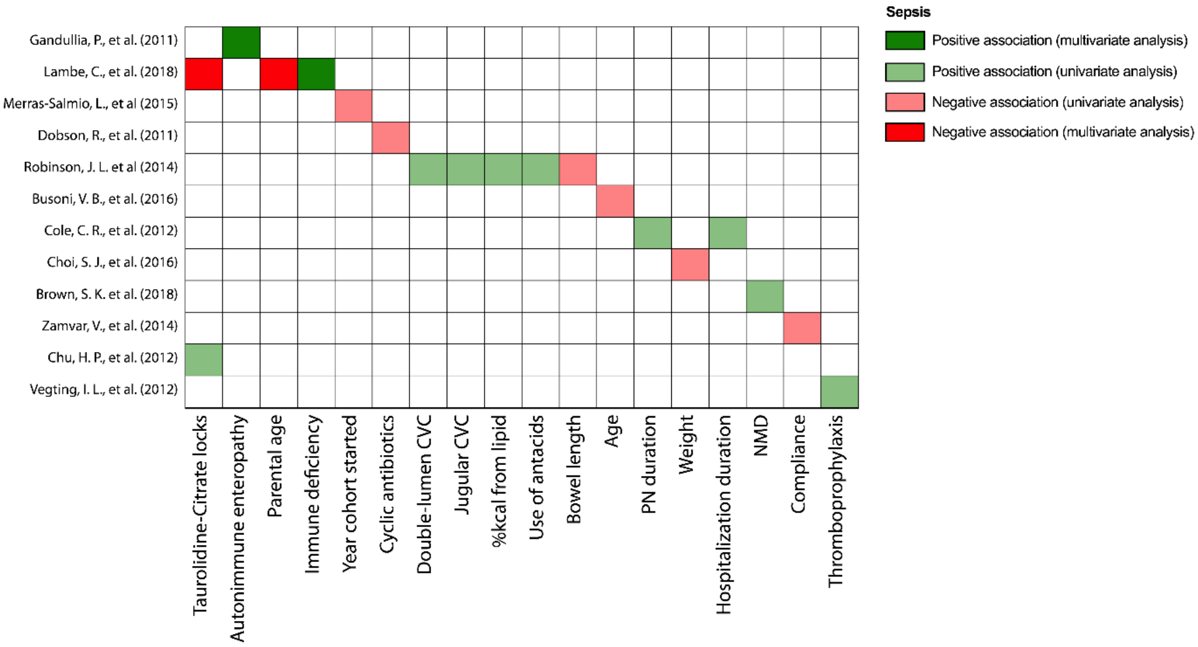

Previously reported associations (or predictors) of sepsis or catheter-related blood stream infection from studies included in the systematic review.

Supplemental figure 15: Previously reported associations of transplantation

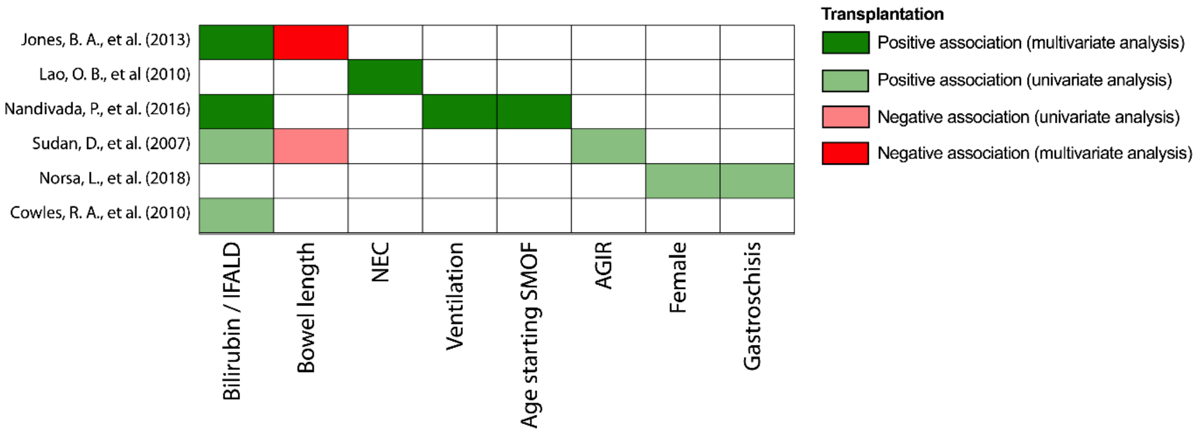

Previously reported associations (or predictors) of transplantation (either intestinal or liver) from studies included in the systematic review.

Supplemental table 1: Summary of Baseline Characteristics and Outcomes in All Patients

		Weighted mean (95% CI)
Age at start of f/u (months)		19.1 (18.6,19.1)
Male (%)		54.6 (54.5,54.6)
Gestational age (weeks)		34.2 (34.1,34.2)
Birth weight (g)		2138 (2123,2138)
Short bowel syndrome (%)		43.2 (42.8,43.2)
Necrotizing enterocolitis (%)		15 (14.9,15.0)
Neuro-muscular disorder (%)		10.7 (10.5,10.7)
Small bowel length (cm)		46.9 (46.2,46.9)
Ileocecal valve (%)		38.8 (38.4,38.8)
Duration of PN (days)		709 (689,709)
Time to enteral autonomy (days)		672 (642,672)
Height at start of f/u (z-score)		-2.01 (-2.05,-2.01)
Height at end of f/u (z-score)		-1.03 (-1.07,-1.03)
Weight at start of f/u (z-score)		-1.57 (-1.61,-1.57)
Weight at end of f/u (z-score)		-0.86 (-0.89,-0.86)
CVC (number of lines per patient per year)		0.56 (0.55,0.58)
Cause of mortality (% of deaths)	Liver failure	22.9 (22.6,22.9)
	Sepsis	17.5 (17.3,17.5)
	Other	19.8 (19.6,19.8)
	Unknown	34.5 (34.1,34.5)
Mortality post-transplant (% of deaths)		12.5 (12.2,12.5)
Type of transplant (% of total transplants)	Isolated liver	12.3 (12.0,12.3)
	Combined liver-small bowel	31.5 (31.1,31.5)
	Isolated small bowel	55.4 (54.9,55.4)
	Sequential small bowel-liver	0.3

Data represents weighted means and 95% confidence intervals. IFALD, intestinal failure associated liver disease.

Supplemental table 2: Characteristics of Included Studies

Name of author, year of publication	Centre & country	Years from → to	RoB score	N	Inclusion criteria/ definition	Person-years of follow-up	Mortality & causes (n, %)	IFALD (n, %)	CRBSI	Enteral autonomy (n, %)	Transplants & type (n, %)	CVC (n)	Neuro outcomes & other comments	Ultra-SBS (Yes/No)
Meehan, J. J., et al., 1997(1)	Alabama, USA	1985 --> 1995	5	47	PN > 3 months	139.5	5 (11); 40% sepsis, 60% other			31 (66)	0 (0);			No
Georgeson, K. E., et al., 1992(2)	Alabama, USA	1978 --> 1990	5	52	Neonatal SBS	123.4	9 (17); 22% liver failure, 22% sepsis, 56% other	26 (50)		39 (75)	0 (0);			No
Capriati, T., et al., 2017(3)	Bambino Gesu, Italy	1996 --> 2016	7	47	Small bowel length <40cm distal to ligament of Treitz at the last resection or exploration	188.0	4 (9); 50% liver failure, 25% sepsis, 25% other	21 (45)		22 (47)	0 (0);	94		No
Casaccia, G., et al., 2006(4)	Bambino Gesu, Italy	1997 --> 2003	4	19	SBS - severe malabsorption, with a residual intestinal length less than 70 cm and need of PN for at least 60 days after bowel reconstruction	30.5	3 (16); 100% unknown	(0)			0 (0);		19 patients <3rd percentile growth; 19 motor developmental delay	No
Diamanti, A., et al., 2003(5)	Bambino Gesu, Italy	1989 --> 2006	5	96	PN>3mth with PN at least 50% kcal	510.4	16 (17); 13% liver failure, 50% sepsis, 38% other	25 (26)	1.4	55 (57)	1 (1); 100% combined liver-bowel			No
Gambarara, M., et al., 2002(6)	Bambino Gesu, Italy	1990 --> 2001	3	114	Non-malignant IF	405.9	18 (16); 39% sepsis, 33% other, 28% unknown			87 (76)	2 (2); 50% isolated liver, 50% isolated small bowel			No

Name of author, year of publication	Centre & country	Years from → to	RoB score	N	Inclusion criteria/ definition	Person-years of follow-up	Mortality & causes (n, %)	IFALD (n, %)	CRBSI	Enteral autonomy (n, %)	Transplants & type (n, %)	CVC (n)	Neuro outcomes & other comments	Ultra-SBS (Yes/No)
Hong, C.R., et al., 2019(7)	Boston, USA	2010--> 2017	4	23	<20% bowel length remaining, volvulus, and >90 days PN	195.5	2 (9); 50% liver failure, 50% sepsis,	16 (70)		7 (30)	5 (17); 40% isolated small bowel, 60% combined liver-bowel		100% normal school at 7-18 years, 67% at 18yrs, and 0% at 0-6years	Yes
Sparks, E. A., et al., 2016(8)	Boston, USA	2002 --> 2014	8	109	PN >90d	472.3	1 (1); 100% unknown			45 (41)	2 (2); 100% isolated small bowel			No
Fullerton, B. S., et al., 2016(9)	Boston, USA	2002 --> 2014	5	313	PN >90 days	1439.8	18 (6); 31% liver failure, 54% sepsis, 31% other, 15% unknown			174 (56)	17 (5); 18% isolated liver, 18% isolated small bowel, 65% combined liver-bowel			No
Fallon, E. M., et al., 2014(10)	Boston, USA	2004 --> 2012	8	63	PN >2 wk, <100cm neonates	135.5	8 (13); 100% unknown			40 (63)	4 (6); 100% combined liver-bowel			No
Modi, B. P., et al., 2008(11)	Boston, USA	1999 --> 2006	4	54	SBS - dependence on PN for at least 90 days	151.2	6 (11); 83% liver failure, 17% other			36 (67)	5 (9); 100% isolated small bowel			No
Andorsky, D. J., et al., 2001(12)	Boston, USA	1986 --> 1998	4	30	IF - dependence on PN for >90 days; neonates	78.6	9 (30); 67% liver failure, 22% sepsis, 11% other			20 (67)	1 (3); 100% combined liver-bowel			No
Busoni, V. B., et al., 2016(13)	Buenos Aires, Argentina	2008 --> 2016	4	41	PN >3 months (inclusion criteria); referred early	97.3	12 (29); 100% unknown	20 (49)		15 (37)	3 (7); 100% isolated small bowel			No

Name of author, year of publication	Centre & country	Years from → to	RoB score	N	Inclusion criteria/ definition	Person-years of follow-up	Mortality & causes (n, %)	IFALD (n, %)	CRBSI	Enteral autonomy (n, %)	Transplants & type (n, %)	CVC (n)	Neuro outcomes & other comments	Ultra-SBS (Yes/No)
Busoni, V. B., et al., 2016(13)	Buenos Aires, Argentina	2008 --> 2016	4	53	PN >3 months (inclusion criteria); referred late	125.8	6 (11); 100% unknown	24 (45)		31 (58)	3 (6); 100% isolated small bowel			No
Sigalet, D., et al., 2009(14)	Calgary, Canada	1998 --> 2006	7	33	<40cm of small bowel or >60 days PN	225.5	9 (27); 100% liver failure			24 (72)	0 (0);			No
Sigalet, D., et al., 2009(14)	Calgary, Canada	2006 --> 2009	7	31	<40cm of small bowel or >60 days PN	43.9	0 (0);			26 (84)	0 (0);			No
Chaet, M. S., et al., 1994(15)	Cincinnati, USA	1980 --> 1992	5	32	<100cm jejunum & ileum	134.4	4 (13); 25% liver failure, 75% other				0 (0);			No
Mousa, H., et al., 2002(16)	Columbus, USA	1992 --> 2001	5	53	CIPO and on PN	110.4	17 (32); 76% liver failure, 24% sepsis	31 (58)			0 (0);			No
Caniano, D. A., et al., 1989(17)	Columbus, USA	1978 --> 1987	7	14	<25% bowel length	37.4	2 (14); 100% liver failure	8 (57)	6	8 (57)	0 (0);	70		No
Sondheimer, J. M., et al., 1998(18)	Colorda, USA	1985 --> 1996	7	44	PN>3mths, post-resection	140.4	4 (9); 100% liver failure	28 (64)	4.35	27 (61)	0 (0);			No
Barclay, A. R., et al., 2009(19)	Edinburgh, UK	1997 --> 2005	5	23	Regional IF database	53.5	10 (43); 10% liver failure, 40% sepsis, 50% other	17 (74)	24.8	10 (43)	5 (22); 20% isolated liver, 80% combined liver-bowel	144		No
Robinson, J. L., et al., 2014(20)	Edmonton, USA	2007 --> 2011	4	16	Children with IF due to a primary intestinal disease who are expected to require PN for a minimum of 30 days	18.5	0 (0);		4.6	12 (75)	0 (0);			No
Casey, L., et al., 2008(21)	Edmonton, USA	1994 --> 2004	6	33	PN >30d	97.9	12 (36); 75% liver failure, 25% unknown	17 (52)	16	16 (48)	1 (3); 100% combined liver-bowel			No

Name of author, year of publication	Centre & country	Years from → to	RoB score	N	Inclusion criteria/ definition	Person-years of follow-up	Mortality & causes (n, %)	IFALD (n, %)	CRBSI	Enteral autonomy (n, %)	Transplants & type (n, %)	CVC (n)	Neuro outcomes & other comments	Ultra-SBS (Yes/No)
Olieman, J. F., et al., 2010(22)	Erasmus, Rotterdam, Netherlands	2002 --> 2007	7	10	SBS: >70% resection of the small bowel and/or PN needed for >42 days after bowel resection and/or residual small bowel length distal to the ligament of Treitz <50 cm for a premature (ga 27-36w), <75 cm for term neonates, and <100 cm for children >12 mo	15.0	0 (0);	8 (80)	11.8	6 (60)	0 (0);	32		No
Olieman, J. F., et al., 2008(23)	Erasmus, Rotterdam, Netherlands	1980 --> 1989	6	28	SBS: loss of >70% of normal small bowel length and/or PN>42 post-resection	91.4	10 (36); 50% liver failure, 50% sepsis	8 (29)	11.1		0 (0);	56		No
Olieman, J. F., et al., 2008(23)	Erasmus, Rotterdam, Netherlands	1990 --> 1999	6	62	70% resection or PN >42d, (<50cm prem, <75cm term, <100cm at 1 yr)	202.4	19 (31); 11% liver failure, 21% sepsis, 68% other	23 (37)	8.7		0 (0);	124		No
Suita, S., et al., 1999(24)	Fukuoka, Japan	1970 --> 1998	4	17	SBS	85.8	3 (18); 33% liver failure, 67% sepsis	11 (65)			0 (0);			No
Pichler, J., et al., 2012(25)	GOSH, UK	2006 --> 2010	5	219	IF	259.9	26 (12); 27% sepsis, 73% other			166 (76)	0 (0);			No
Heneyke, S., et al., 1999(26)	GOSH, UK	1979 --> 1997	6	32	CIPO	170.9	14 (44); 100% unknown			8 (25)	1 (3); 100% isolated small bowel			No

Name of author, year of publication	Centre & country	Years from → to	RoB score	N	Inclusion criteria/ definition	Person-years of follow-up	Mortality & causes (n, %)	IFALD (n, %)	CRBSI	Enteral autonomy (n, %)	Transplants & type (n, %)	CVC (n)	Neuro outcomes & other comments	Ultra-SBS (Yes/No)
Dollery, C. M., et al., 1994(27)	GOSH, UK	1983 --> 1993	6	34	PN >2m for IF	90.8	6 (18); 83% sepsis, 17% other			6 (18)	0 (0);	21 0		No
Mayr, J. M., et al., 1999(28)	Graz, Austria	1976 --> 1998	6	17	Severe SBS	91.8	4 (24); 100% liver failure	6 (35)		11 (65)	0 (0);		5/17 normal development , 6/17 psychomotor delay (3 with congenital cerebral abnormalitie s)	No
Schurink, M., et al., 2014(29)	Groninge n, Netherlan ds	2001 --> 2009	8	19	Anatomical SBS' i.e. IF post-bowel resection	39.6	2 (11); 100% sepsis	13 (68)		16 (84)	1 (5); 100% isolated liver			No
Merras-Salmio, L., et al., 2018(30)	Helskini, Finland	1984 --> 2017	8	100	>50% resection or PN >2m	660.0	8 (8); 43% liver failure, 29% sepsis, 29% other	26 (26)	1.01	67 (67)	5 (5); 100% isolated small bowel		mean height -1.5 z-score	No
Hukkinen, M., et al., 2017(31)	Helskini, Finland	2002 --> 2015	8	61	>50% resection, or more if PN >2m	229.3	5 (8); 100% unknown			49 (80)	0 (0);			No
Pakarinen, M. P., et al., 2015(32)	Helskini, Finland	1988 --> 2013	7	12	<30% length or PN >3m, and <25cm	82.8	2 (17); 100% liver failure			6 (50)	0 (0);			Yes
Pakarinen, M. P., et al., 2015(32)	Helskini, Finland	1988 --> 2013	7	30	<30% length or PN >3m, and >25cm	141.0	2 (7); 100% unknown			27 (90)	0 (0);			No
Merras-Salmio, L., et al., 2015(33)	Helskini, Finland	1998 --> 2008	7	26	<25% length or PN >3m	236.6	3 (12); 100% unknown	8 (31)	1.7	20 (77)	0 (0);	36		No
Merras-Salmio, L., et al., 2015(33)	Helskini, Finland	2009 --> 2014	7	22	<25% length or PN >3m	59.4	1 (5); 100% unknown	16 (73)	0.7	16 (73)	0 (0);	31		No

Name of author, year of publication	Centre & country	Years from → to	RoB score	N	Inclusion criteria/ definition	Person-years of follow-up	Mortality & causes (n, %)	IFALD (n, %)	CRBSI	Enteral autonomy (n, %)	Transplants & type (n, %)	CVC (n)	Neuro outcomes & other comments	Ultra-SBS (Yes/No)
Mutanen, A., et al., 2018(34)	Helsinki, Finland	1997 --> 2016	4	20	IF: PN >3 months and/or small bowel resection >50% of age-adjusted length		2 (10); 50% liver failure, 50% other			18 (90)				No
Pakarinen, M. P., et al., 2013(35)	Helskini, Finland	1984 --> 2011	7	20	<25% length or PN >6m	274.3	2 (10); 100% unknown			11 (55)	2 (10); 100% isolated small bowel			No
Kurvinen, A., et al., 2012(36)	Helskini, Finland	1994 --> 2012	5	11	PN >28d	50.6	0 (0);	3 (27)	0.6	1 (9)	1 (9); 100% isolated small bowel			No
Pakarinen, M. P., et al., 2009(37)	Helskini, Finland	2000 --> 2007	8	20	<25% length or PN >6m	46.0	2 (10); 100% liver failure	11 (55)		12 (60)	0 (0);			No
Pakarinen, M. P., et al., 1995(38)	Helskini, Finland	1987 --> 1992	8	12	>50% resection or PN >3m	26.4	4 (33); 25% sepsis, 75% unknown		6.1	4 (33)	0 (0);			No
Grosfeld, J. L., et al., 1986(39)	Indianapolis, USA	1972 --> 1984	4	60	SBS: 50% expected bowel length	213.6	9 (15); 56% liver failure, 33% sepsis, 11% other			()	0 (0);			No
Kaji, T., et al., 2017(40)	Kagoshima, Japan	1987 --> 2017	7	16	SB<75cm	122.4	6 (38); 40% liver failure, 60% sepsis, 20% other	5 (31)		9 (56)	1 (6); 100% isolated small bowel			No
Koglmeier, J., et al., 2008(41)	Leeds, UK	2001 --> 2004	5	93	IF - PN dependency for 28 days or more	186.0	6 (6); 50% sepsis, 50% other	55 (59)	7.3	85 (91)	1 (1); 100% isolated liver			No
Auth, M., et al., 2016(42)	Liverpool, UK	1996 --> 2015	6	113	PN >27 days (inclusion criteria)	1384.3	15 (13); 33% liver failure, 33% sepsis, 33% unknown	25 (22)		96 (85)	3 (3); 33% isolated small bowel, 67% combined liver-bowel			No

Name of author, year of publication	Centre & country	Years from → to	RoB score	N	Inclusion criteria/ definition	Person-years of follow-up	Mortality & causes (n, %)	IFALD (n, %)	CRBSI	Enteral autonomy (n, %)	Transplants & type (n, %)	CVC (n)	Neuro outcomes & other comments	Ultra-SBS (Yes/No)
Dore, M., et al., 2017(43)	Madrid, Spain	2000 --> 2015	7	30	<10cm bowel	70.0	14 (47); 100% unknown	29 (97)		15 (50)	21 (70); 29% isolated small bowel, 71% combined liver-bowel			Yes
Khalil, B. A., et al., 2012(44)	Manchester, UK	2000 --> 2009	6	27	SBS	80.1	2 (7); 100% unknown			21 (78)	1 (4); 100% combined liver-bowel			No
Ives, G. C., et al., 2016(45)	Michigan, USA	2000 --> 2015	6	29	SBS was defined as dependence on PN for ≥60 days secondary to loss of ≥50% SBL	133.4	2 (7); 100% unknown			16 (55)	0 (0);			No
Demehri, F. R., et al., 2015(46)	Michigan, USA	1988 --> 2013	8	171	SBS - loss of at least 50% of expected small intestinal length, or at least 60 days of PN dependence with some history of loss of intestinal length	701.1	26 (15); 100% unknown			110 (64)	5 (3); 100% isolated small bowel			No

Name of author, year of publication	Centre & country	Years from → to	RoB score	N	Inclusion criteria/ definition	Person-years of follow-up	Mortality & causes (n, %)	IFALD (n, %)	CRBSI	Enteral autonomy (n, %)	Transplants & type (n, %)	CVC (n)	Neuro outcomes & other comments	Ultra-SBS (Yes/No)
Hess, R. A., et al., 2011(47)	Michigan, USA	1990 --> 2009	6	171	SBS - loss of ≥50% of small intestinal length from surgical resection or congenital defect requiring ≥2 months of PN dependence due to intestinal dysfunction. Other etiologies of IF – included a dependency of ≥ 2 mo of PN due to intestinal dysfunction from dysmotility or malabsorption	199.5	21 (12); 100% unknown	90 (53)		114 (67)	0 (0);			No
Spencer, A. U., et al., 2005(48)	Michigan, USA	1997 --> 2005	8	80	>50% resection or PN >2m	408.0	22 (28); 64% liver failure, 18% sepsis, 14% other, 5% unknown			51 (64)	0 (0);			No
Coran, A. G., et al., 1999(49)	Michigan, USA	1983 --> 1993	4	22	SBS	70.2	5 (23); 80% liver failure, 20% sepsis	12 (55)	7.4	5 (23)	0 (0);			No
Teitelbaum, D. H., et al., 1996(50)	Michigan, USA	1986 --> 1996	2	25	SBS: loss of >70% of normal small bowel length	74.2	8 (32); 50% liver failure, 50% sepsis	18 (72)			0 (0);			No
Weber, T. R., et al., 2002(51)	Missouri, USA	1997 --> 2002	3	12	SBS due to NEC	17.8	0 (0);				0 (0);			No

Name of author, year of publication	Centre & country	Years from → to	RoB score	N	Inclusion criteria/ definition	Person-years of follow-up	Mortality & causes (n, %)	IFALD (n, %)	CRBSI	Enteral autonomy (n, %)	Transplants & type (n, %)	CVC (n)	Neuro outcomes & other comments	Ultra-SBS (Yes/No)
Weber, T. R., et al., 1991(52)	Missouri, USA	1981 --> 1991	4	16	<100cm at <2m age	88.0	1 (6); 100% unknown			14 (88)	0 (0);	28	11/15 patients above 50th percentile for height and weight at 2-10 years after bowel resection	No
Furtado, S., et al., 2016(53)	Montreal, Canada	2006 --> 2009	4	27	PN>42 days, <25% length	27.0	4 (15); 100% other	23 (85)	26.2	23 (85)	0 (0);			No
Furtado, S., et al., 2016(53)	Montreal, Canada	2009 --> 2012	4	28	PN>42 days, <25% length	43.9	2 (7); 50% sepsis, 50% other	23 (82)	16.5	24 (86)	0 (0);			No
Totonelli, G., et al., 2018(54)	Multi (Bambino Gesu, Italy)	1994 --> 2015	4	114	>75% PN for >4wk	279.3	9 (8); 25% liver failure, 75% sepsis	51 (45)	0.85	78 (68)	2 (2); 100% isolated liver	31 4		No
Squires, R. H., et al., 2012(55)	Multi (US & Canda)	2000 --> 2007	8	272	Infants with IF were included if they were < 1 year of age and required prolonged support with PN, defined as >60 days out of 74 consecutive days	759.3	68 (25); 48% liver failure, 19% sepsis, 28% other, 22% unknown	125 (46)	8.9	118 (43)	60 (22); 100% isolated small bowel			No

Name of author, year of publication	Centre & country	Years from → to	RoB score	N	Inclusion criteria/ definition	Person-years of follow-up	Mortality & causes (n, %)	IFALD (n, %)	CRBSI	Enteral autonomy (n, %)	Transplants & type (n, %)	CVC (n)	Neuro outcomes & other comments	Ultra-SBS (Yes/No)
Cole, C. R., et al., 2012(56)	Multi	2002 --> 2012	5	78	IF - infant with NEC had gastrointestinal surgery that resulted in PN dependence > 6 weeks duration	78.0	20 (26); 100% unknown		12.8	56 (72)	0 (0);			No
Salvia, G., et al., 2008(57)	Multi	2003 --> 2008	5	26	Neonates, TPN>1mth or partial PN>3mths, primary intestinal disease	74.8	2 (8); 50% liver failure, 50% other	14 (54)		22 (85)	1 (4); 100% isolated small bowel			No
Guarino, A., et al., 2003(58)	Multi (Italian)	1997 --> 2001	6	109	IF - clinical condition resulting from a primary intestinal disease for which PN, providing at least 75% of total calories for not less than 4 weeks or at least 50% of total calories for not less than 3 months, is needed.	456.9	8 (7); 100% unknown			46 (42)	4 (4); 100% isolated small bowel			No

Name of author, year of publication	Centre & country	Years from → to	RoB score	N	Inclusion criteria/ definition	Person-years of follow-up	Mortality & causes (n, %)	IFALD (n, %)	CRBSI	Enteral autonomy (n, %)	Transplants & type (n, %)	CVC (n)	Neuro outcomes & other comments	Ultra-SBS (Yes/No)
Galea, M. H., et al., 1992(59)	Multi, UK	1978 --> 1988	4	64	<25% + 1 SD (<50cm for <35wk, and <72cm for >35wk)	82.3	14 (22); 14% liver failure, 50% sepsis, 36% other	20 (31)		42 (66)	0 (0);		Better height gain in adapted cohort but weight no different; at end: height -1.2 (-6.3,+0.6); weight -0.5 (-4.1,+1.2)	No
Infantino, B. J., et al., 2013(60)	Nebraska, USA	2001 --> 2011	5	28	<20cm and <2yrs at referral	56.5	1 (4); 100% unknown			13 (46)	4 (14); 75% isolated small bowel, 25% combined liver-bowel			Yes
Bruzoni, M., et al., 2008(61)	Nebraska, USA	1980 --> 2008	4	30	SBS <180cm	615.0	1 (3);				8 (27); 63% isolated small bowel, 38% combined liver-bowel			No
Thompson, J. S., et al., 1995(62)	Nebraska, USA	1980 --> 1994	6	112	<120cm and/or needed PN on discharge	653.3	14 (13); 63% liver failure, 63% sepsis, 50% other			57 (51)	16 (14); 19% isolated small bowel, 81% combined liver-bowel			No
Norsa, L., et al., 2018(63)	Necker, France	1987 --> 2007	7	36	Ultra-short bowel syndrome	510.6	8 (22);	13 (36)	2.99	6 (17)	20 (56); 65% isolated small bowel, 35% combined liver-bowel		end of follow-up z-scores weight -1.04 +/- 1, height -0.43 +/- 1.3	Yes

Name of author, year of publication	Centre & country	Years from → to	RoB score	N	Inclusion criteria/ definition	Person-years of follow-up	Mortality & causes (n, %)	IFALD (n, %)	CRBSI	Enteral autonomy (n, %)	Transplants & type (n, %)	CVC (n)	Neuro outcomes & other comments	Ultra-SBS (Yes/No)
Goulet, O., et al., 2005(64)	Necker, France	1975 --> 2005	5	87	Neonatal resection and needed PN	1313.7	9 (10); 100% liver failure, 29% other			69 (79)	2 (2); 100% isolated small bowel			No
Baglin-Gobet, S., et al., 1999(65)	Necker, France	1975 --> 1991	6	87	Neonatal extensive resection	1131.0	9 (10); 100% unknown			69 (79)	0 (0);			No
Cowles, R. A., et al., 2010(66)	New York, USA	2003 --> 2009	5	93	IF	165.6	14 (15); 100% unknown	76 (82)			15 (16); 100% isolated small bowel			No
Stollman, T. H., et al., 2009(67)	Nijmegen, Netherlands	1971 --> 2004	4	17	JIA and stenosis	17.0	5 (29); 100% unknown			16 (94)	0 (0);			No
Phillips, J. D., et al., 2008(68)	North Carolina, USA	1993 --> 2007	5	21	Gastroschisis with intestinal atresia and dysmotility	148.8	10 (48); 40% liver failure, 40% sepsis, 20% other			11 (52)	0 (0);			No
Kaufman, S. S., et al., 1997(69)	Omaha, USA	1976 --> 1994	5	49	PN >3 months (inclusion criteria)	87.2	3 (6); 67% liver failure, 33% sepsis			42 (86)	0 (0);			No
Ueno, T., et al., 2018(70)	Osaka, Japan	2001 --> 2018	5	77	Patients <18 years old with IF treated with PN for >6 months	231	3 (4); 100% unknown	6 (8)			1 (1); 100% isolated small bowel			No
Wasa, M., et al., 1999(71)	Osaka, Japan	1969 --> 1998	5	12	<75cm	120.0	0 (0);	7 (58)	0.63	8 (67)	0 (0);	83		No
Fusaro, F., et al., 2000(72)	Padua, Italy	1981 --> 1997	6	14	Neonatal SBS	14.0	3 (21); 100% other			8 (57)	0 (0);	55		No

Name of author, year of publication	Centre & country	Years from → to	RoB score	N	Inclusion criteria/ definition	Person-years of follow-up	Mortality & causes (n, %)	IFALD (n, %)	CRBSI	Enteral autonomy (n, %)	Transplants & type (n, %)	CVC (n)	Neuro outcomes & other comments	Ultra-SBS (Yes/No)
Cooper, A., et al., 1984(73)	Philadelphia, USA	1973 --> 1983	6	16	>50% jejunum & ileum resection	52.8	3 (19); 100% liver failure	15 (94)		8 (50)	0 (0);		at end follow up mean weight = 26th percentile; height = 24th and W/H = 41st	No
Nucci, A., et al., 2008(74)	Pittsburgh, USA	1996 --> 2006	6	389	IF	778.0	109 (28); 100% unknown	284 (73)		192 (49)	119 (31); 100% isolated small bowel			No
Koehler, A. N., et al., 2000(75)	Pittsburgh, USA	1996 --> 1999	3	103	IF	206.0	27 (26); 100% unknown	68 (66)		49 (48)	22 (21);			no
Kurkchubasche, A. G., et al., 1993(76)	Pittsburgh, USA	1985 --> 1992	6	21	Severe SBS: SB<60cm	43.6	6 (29); 40% liver failure, 20% sepsis, 80% other	3 (14)	6.4	11 (52)	1 (5); 100% combined liver-bowel			no
Javid, P. J., et al., 2011(77)	Seattle, USA	2005 --> 2009	6	62	IF	86.8	9 (15); 100% unknown	28 (45)			0 (0);			no
Wada, M., et al., 2013(78)	Sendai, Japan	1978 --> 2012	5	57	Residual small bowel length <40 cm or requirement for PN support >42 days	575.1	8 (14); 57% liver failure, 43% sepsis, 14% other	29 (51)		28 (49)	7 (12); 86% isolated small bowel, 14% sequental liver-bowel			no
Dimatatac, D. M., et al., 2016(79)	Singapore	2007 --> 2015	6	29	PN >60 days, neonates	34.8	2 (7); 100% other	23 (79)		24 (83)	0 (0);			no
Pickard, S. S., et al., 2009(80)	Stanford, USA	1999 --> 2007	5	13	PN dependence >3 months	31.8	6 (46); 100% unknown				4 (31); 100% isolated small bowel			no

Name of author, year of publication	Centre & country	Years from → to	RoB score	N	Inclusion criteria/ definition	Person-years of follow-up	Mortality & causes (n, %)	IFALD (n, %)	CRBSI	Enteral autonomy (n, %)	Transplants & type (n, %)	CVC (n)	Neuro outcomes & other comments	Ultra-SBS (Yes/No)
Tillman, E. M., et al., 2014(81)	Tennessee, USA	2007 --> 2009	4	33	Infants with NEC, PN > 2 weeks	58.8	6 (18); 50% liver failure, 50% other	24 (73)			0 (0);			no
Tillman, E. M., et al., 2014(81)	Tennessee, USA	2010 --> 2011	4	31	Infants with NEC, PN > 2 weeks	31.0	1 (3);	13 (42)			0 (0);			no
McLaughlin, C. M., et al., 2018(82)	Texas, USA	2008 --> 2016	4	41	<25% bowel length / PN for >6wk	82.0	;			14 (34)	0 (0);			no
Anagnostopoulos, D., et al., 1991(83)	Thessaloniki, Greece	1975 --> 1989	5	59	SBS	245.1	12 (20); 42% liver failure, 50% sepsis, 8% other				0 (0);			no
Belza, C., et al., 2017(84)	Toronto, Canada	2006 --> 2013	6	120	>6 weeks PN and <25% bowel length for age	313.0	11 (9); 18% liver failure, 85% other, 18% unknown	70 (58)		84 (70)	3 (3); 100% isolated liver		Height -0.59 and weight -0.64 in those with enteral autonomy	no
So, S., et al., 2016(85)	Toronto, Canada	2011 --> 2013	4	33	PN>6wks or <25% small bowel	36.5	0 (0);				1 (3); 100% isolated liver			no

Name of author, year of publication	Centre & country	Years from → to	RoB score	N	Inclusion criteria/ definition	Person-years of follow-up	Mortality & causes (n, %)	IFALD (n, %)	CRBSI	Enteral autonomy (n, %)	Transplants & type (n, %)	CVC (n)	Neuro outcomes & other comments	Ultra-SBS (Yes/No)
So, S., et al., 2019(86)	Toronto, Canada	2011--> 2013	7	31	PN duration >6 weeks or <25% bowel length remaining	82.7	0 (0)			21 (68)	0 (0)		At 1yr, 36% below average on cognitive composite and 80% below average with gross motor skills. At 2yrs, >50% below average on cognitive composite and 52% below average on motor assessment.	No
Thacker, K., et al., 2015(87)	Toronto, Canada	2004 --> 2013	3	13	USBS: residual SB<20cm	29.9	0 (0);	9 (69)		2 (15)	3 (23); 100% isolated small bowel		Height and weight Z-scores were normal in all patients	Yes
Thacker, K., et al., 2015(87)	Toronto, Canada	1996 --> 2004	3	8	USBS: residual SB<20cm	8.0	7 (88); 100% unknown				0 (0);		Height and weight Z-scores were normal in all patients	Yes

Name of author, year of publication	Centre & country	Years from → to	RoB score	N	Inclusion criteria/ definition	Person-years of follow-up	Mortality & causes (n, %)	IFALD (n, %)	CRBSI	Enteral autonomy (n, %)	Transplants & type (n, %)	CVC (n)	Neuro outcomes & other comments	Ultra-SBS (Yes/No)
Burghardt, K. M., et al., 2015(88)	Toronto, Canada	1998 --> 2005	8	99	<75% or PN >42d	161.7	33 (33); 100% unknown	49 (49)			7 (7); 43% isolated liver, 29% isolated small bowel, 29% combined liver-bowel			no
Burghardt, K. M., et al., 2015(88)	Toronto, Canada	2006 --> 2012	8	91	<75% or PN >42d	148.6	16 (18); 100% unknown	37 (41)			10 (11); 30% isolated liver, 10% isolated small bowel, 60% combined liver-bowel			no
Elfvin, A., et al., 2015(89)	Toronto, Canada	2000 --> 2009	7	97	requiring PN for >42 and for >90 days	97.0	21 (22); 100% unknown	57 (59)			1 (1); 100% isolated liver			no
Burghardt, K. M., et al., 2014(90)	Toronto, Canada	2010 --> 2014	7	59	>75% bowel loss (for GA) or TPN >42d	59.0	0 (0);	40 (68)			6 (10); 50% isolated liver, 17% isolated small bowel, 33% combined liver-bowel			no
Diamond, I. R., et al., 2010(91)	Toronto, Canada	2003 --> 2007	8	106	SBS - intestinal length of less than the 25th percentile for gestational age, or who remain on PN at 42 days after resection	106.0	26 (25); 54% liver failure, 15% sepsis, 31% other			70 (66)	0 (0);			no

Name of author, year of publication	Centre & country	Years from → to	RoB score	N	Inclusion criteria/ definition	Person-years of follow-up	Mortality & causes (n, %)	IFALD (n, %)	CRBSI	Enteral autonomy (n, %)	Transplants & type (n, %)	CVC (n)	Neuro outcomes & other comments	Ultra-SBS (Yes/No)
Diamond, I. R., et al., 2007(92)	Toronto, Canada	2003 --> 2005	7	54	SBS— intestinal length that was less than the 25th percentile for their gestational age or those who continue to receive parenteral nutrition (PN) at 42 days after resection	73.4	18 (33); 33% liver failure, 33% sepsis, 33% other	43 (80)		35 (65)	4 (7); 25% isolated liver, 75% combined liver-bowel	108		no
Diamond, I. R., et al., 2007(92)	Toronto, Canada	1997 --> 1998	7	40	SBS—intestinal length that was less than the 25th percentile for their gestational age or those who continue to receive parenteral nutrition (PN) at 42 days after resection	65.7	15 (38); 60% liver failure, 20% sepsis, 20% other	25 (63)		25 (63)	0 (0);	100		no
Nasr, A., et al., 2007(93)	Toronto, Canada	1997 --> 2005	4	70	SBS is need for PN>6wks post-resection or bowel length <25% expected by GA	166.2	;	37 (53)			0 (0);			no
Wales, P. W., et al., 2004(94)	Toronto, Canada	1997 --> 2001	8	40	SBS: the need for TPN >42 days after bowel resection, or a residual small bowel length <25% expected for gestational age	65.3	15 (38); 60% liver failure, 20% sepsis, 20% other	25 (63)		25 (63)	0 (0);			no
Bagwell, C. E., et al., 1984(95)	Toronto, Canada	1974 --> 1984	6	10	Neonatal intestinal pseudo-obstruction	15.0	4 (40); 50% liver failure, 50% sepsis			3 (30)	0 (0);			no

Name of author, year of publication	Centre & country	Years from → to	RoB score	N	Inclusion criteria/ definition	Person-years of follow-up	Mortality & causes (n, %)	IFALD (n, %)	CRBSI	Enteral autonomy (n, %)	Transplants & type (n, %)	CVC (n)	Neuro outcomes & other comments	Ultra-SBS (Yes/No)
Quiros-Tejeira, R. E., et al., 2004(96)	UCLA, USA	1975 --> 2000	8	78	PN > 3mths	702.0	21 (27); 29% liver failure, 38% sepsis, 29% other, 5% unknown	18 (23)		44 (56)	3 (4); 33% isolated liver, 67% combined liver-bowel			no
Thakur, A., et al., 2002(97)	UCLA, USA	1977 --> 1999	5	17	PN > 3 months	117.3	4 (24); 75% liver failure, 25% other			9 (53)	1 (6); 100% isolated small bowel			no
Angsten, G., et al., 2012(98)	Uppsala, Sweden	1985 --> 2005	6	18	SBS - residual small bowel length distal to the ligament of Treitz <50 cm for a preterm infant (ga 22–37 weeks), <75 cm for term-born neonates, and/ or need for PN supplementation of >50% of nutrition requirements by PN for >60 days	91.2	9 (50); 67% liver failure, 33% sepsis			9 (50)	0 (0);	63		no

Name of author, year of publication	Centre & country	Years from → to	RoB score	N	Inclusion criteria/ definition	Person-years of follow-up	Mortality & causes (n, %)	IFALD (n, %)	CRBSI	Enteral autonomy (n, %)	Transplants & type (n, %)	CVC (n)	Neuro outcomes & other comments	Ultra-SBS (Yes/No)
Angsten, G., et al., 2012(98)	Uppsala, Sweden	2006 --> 2010	6	20	SBS - residual small bowel length distal to the ligament of Treitz <50 cm for a preterm infant (ga 22–37 weeks), <75 cm for term-born neonates, and/ or need for PN supplementation of >50% of nutrition requirements by PN for longer than 60 days.	26.7	3 (15); 67% liver failure, 33% sepsis			11 (55)	0 (0);	40		no
Vugrinec Mamic, M., et al., 2018(99)	Zagreb, Croatia	1992 --> 2015	4	12	Patients treated with prolonged (<2 months) PN	53.4	4 (33); 50% liver failure, 50% sepsis	2 (17)	1.8	4 (33)				No
Vugrinec Mamic, M., et al., 2018(99)	Zagreb, Croatia	1992 --> 2015	4	26	Patients treated with prolonged (<2 months) PN	20.6	0 (0)	0 (0)	2.2	25 (96)				No
Vugrinec Mamic, M., et al., 2018(99)	Zagreb, Croatia	1992 --> 2015	4	44	Patients treated with prolonged (<2 months) PN	10.3	0 (0)	0 (0)	3.5	44 (100)				No

Name of author, year of publication	Centre & country	Years from → to	RoB score	N	Inclusion criteria/ definition	Person-years of follow-up	Mortality (n, %) & causes (%)	CRBSI per 1000 catheter days	Enteral autonomy (n, %)	Transplants (n, %) & type (%)	CVC, n	Neuro outcomes & other comments
Rajanayagam, J., et al., 2015(100)	Birmingham, UK	1989 --> 2013	3	221	IFALD	1105.0	40 (18); 100% unknown			62 (28); 31% isolated liver, 69% combined liver-bowel		
Nandivada, P., et al., 2016(101)	Boston, USA	2004 --> 2014	8	182	Bili >2mg/dL for 2wk	540.1	27 (15); 67% liver failure, 11% sepsis, 22% other			8 (4); 25% isolated liver, 75% combined liver-bowel		
Nandivada, P., et al., 2016(102)	Boston, USA	2004 --> 2015	6	30	Bili >2mg/dL for 2wk	97.9	0 (0)		18 (60)	0 (0)		
Martinez, M. I., et al., 2015(103)	Buenos Aires, Argentina	2006 --> 2014	4	29	IF	65.7	10 (34); 30% liver failure, 20% sepsis, 30% other		16 (55)	18 (62); 100% isolated small bowel		
Pichler, J., et al., 2012(25)	GOSH, UK	2006 --> 2010	5	60	IF + raised ALT/ALP/GGT +/- raised bili	71.2	13 (22); 23% liver failure, 46% sepsis, 31% other		40 (67)	0 (0)		
Weber, T. R., et al., 2002(51)	Missouri, USA	1997 --> 2002	3	30	SBS due to NEC and Child's B or C	44.5	9 (30); 33% liver failure, 67% other			0 (0)		

Name of author, year of publication	Centre & country	Years from → to	RoB score	N	Inclusion criteria/ definition	Person-years of follow-up	Mortality (n, %) & causes (%)	CRBSI per 1000 catheter days	Enteral autonomy (n, %)	Transplants (n, %) & type (%)	CVC, n	Neuro outcomes & other comments
De Marco, G., et al., 2006(104)	Naples, Italy	1997 --> 2004	8	12	PN provided at least 75% of the total caloric input for not <4 weeks,or at least 50% of calories for not <3 months.	31.3	3 (25); 100% unknown	5.6	7 (58)	0 (0)		
Iyer, K. R., et al., 2004(105)	Nebraska, USA	2002 --> 2004	5	13	SBS and bili >2 or abnormal Bx	13.0	3 (23); 67% sepsis, 33% other		3 (23)	0 (0)		
Colomb, V., et al., 1994(106)	Necker, France	1979 --> 1994	5	21	PN dependent	85.8	2 (10); 50% liver failure, 50% unknown			1 (5); 100% isolated small bowel		
Sorrell, M., et al., 2017(107)	San Antonio, USA	2011 --> 2013	3	13	>50% resected, or dysmotile, bili >4mg/dL, >60% PN for >28d	13.0	0 (0)	2.3		0 (0)		
Nasr, A., et al., 2010(108)	Toronto, Canada	2001 --> 2005	8	104	Neonates with conj bili >34 on PN post-resection	527.8	20 (19); 25% liver failure, 75% unknown		86 (83)	4 (4); 100% isolated liver		

Name of author, year of publication	Centre & country	Years from → to	RoB score	N	Inclusion criteria/ definition	Person-years of follow-up	Mortality (n, %) & causes (%)	CRBSI per 1000 catheter days	Enteral autonomy (n, %)	Transplants (n, %) & type (%)	CVC, n	Neuro outcomes & other comments
Wang, C., et al., 2018(109)	UCLA, USA	2009 --> 2016	8	48	GI disorder requiring long-term PN and cholestasis with bilirubin >2 mg/dL	64	4 (8); 75% sepsis, 25% other		8 (17)	4 (8); 25% isolated liver, 75% combined liver-bowel		
Spurrier, R., et al., 2013(110)	UCLA, USA	2010 --> 2013	6	28	Bili >2mg/dL, PN >30d for >60% kcal	46.0	4 (14); 100% unknown			1 (4); 100% isolated liver		
Rollins, M. D., et al., 2010(111)	Utah, USA	2003 --> 2008	4	23	PN>6mths + IFALD (conj bili>2mg/dL + transaminases >2xN)	34.1	3 (13); 33% liver failure		10 (43)	4 (17); 75% isolated liver, 25% isolated small bowel		All met normal neuro-develop-mental milestones
Kaufman, S. S., et al., 2010(112)	Washington, USA	2003 --> 2007	6	61	SBS (6wk PN after resection) + cholestasis	78.4	12 (20); 100% liver failure		11 (18)	36 (59); 14% isolated liver, 3% isolated small bowel, 83% combined liver-bowel		

Name of author, year of publication	Centre & country	Years from → to	RoB score	N	Inclusion criteria/ definition	Person-years of follow-up	Mortality (n, %) & causes (%)	IFALD (n, %)	CRBSI	Enteral autonomy (n, %)	Transplants (n, %) & type (%)	CVC, (n)	Neuro outcomes & other comments	Ultra (Yes/No)
Simmons, M. G., et al., 1996(113)	Alabama, USA	1989 --> 1996	4	18	On HPN with >50% calories as PN	37.4	1 (6); 100% other				0 (0);			No
Vegting, I. L., et al., 2012(114)	Amsterdam, Netherlands	1994 --> 2010	5	32	On home PN	77.9	5 (16); 100% unknown		3.6		0 (0);	154		No
Herbison, K., et al., 2017(115)	Auckland, NZ	2010 --> 2015	4	109	IF: patients receiving PN >21 days as an inpatient	161.7	11 (10); 100% other	19 (17)		70 (64)	0 (0);			No
Diamanti, A., et al., 2014(116)	Bambino Gesu, Italy	2010 --> 2014	8	11	SBS neonatally, HPN, <10cm	58.3	2 (18); 50% liver failure, 50% other	8 (73)	0.79	2 (18)	1 (9); 100% isolated small bowel	24		Yes
Diamanti, A., et al., 2011(117)	Bambino Gesu, Italy	2005 --> 2010	5	28	PN >6m, SBS, normal renal function	28.0	0 (0)			14 (50)	0 (0);			No
Knafelz, D., et al., 2003(118)	Bambino Gesu, Italy	1990 --> 2003	5	61	On HPN	73.2	20 (33); 100% unknown	11 (18)	0.55	29 (48)	1 (2); 100% isolated small bowel	81		No
Gambarara, M., et al., 2001(119)	Bambino Gesu, Italy	1990 --> 2001	6	41	On HPN	58.1	9 (22); 22% liver failure, 78% other		0.55		1 (2); 100% combined liver-bowel	58		No
Gandullia, P., et al., 2011(120)	Genova, Italy	1996 --> 2011	8	36	On home PN (>3m PN and stable)	101.1	5 (14); 100% unknown	3 (8)	1.79	18 (50)	1 (3); 100% isolated small bowel	99		No
Dobson, R., et al., 2011(121)	Glasgow, UK	2001 --> 2007	6	15	>28d PN for IF	26.4	1 (7); 100% other	11 (73)	13.8	9 (60)	0 (0);	43		No
Ashworth, I., et al., 2018(122)	GOSH, UK	1986 --> 2010	5	13	IF & tufting enteropathy on histology	212.3	1 (8); 100% liver failure	0 (0)		6 (46)	0 (0);		All 12 in normal jobs or in mainstream school	No

Name of author, year of publication	Centre & country	Years from → to	RoB score	N	Inclusion criteria/ definition	Person-years of follow-up	Mortality (n, %) & causes (%)	IFALD (n, %)	CRBSI	Enteral autonomy (n, %)	Transplants (n, %) & type (%)	CVC, (n)	Neuro outcomes & other comments	Ultra (Yes/No)
Chu, H. P., et al., 2012(123)	GOSH, UK	2009 --> 2012	5	19	On home PN	44.8	0 (0)		4.2		0 (0);			No
Sala, D., et al., 2010(124)	GOSH, UK	1997 --> 2007	6	13	HPN with volvulus/ gastroschisis	63.1	3 (23); 33% liver failure, 33% sepsis, 33% unknown			7 (54)	0 (0);			No
Bisset, W. M., et al., 1992(125)	GOSH, UK	1987 --> 1992	6	10	On home PN	26.0	1 (10); 100% other	3 (30)	2.1	1 (10)	0 (0);	19	All children maintained weight within normal range, 14/15 within 2 std dev of mean for height; all children within normal	No
Peyret, B., et al., 2011(126)	Lyon, France	1998 --> 2007	5	42	HPN >2 years	331.8	3 (7); 100% other	24 (57)	3	17 (40)	3 (7); 100% isolated small bowel			No
Styblova, J., et al., 2017(127)	Multi, Czech Republic	1995 --> 2011	4	66	On HPN	359.1	13 (20); 23% liver failure, 15% sepsis, 38% other, 23% unknown		1.58	32 (48)	0 (0);	78		No
Goulet, O., 2016(128)	Multi, France	1996 --> 2014	6	268	On HPN	1742.0	13 (5); 100% unknown	20 (7)	0.94		0 (0);			No
Neelis, E. G., et al., 2016(129)	Multi	2001 --> 2013	4	37	>75% PN for >4wk	112.5	1 (3);				5 (14); 60% isolated small bowel, 40% combined liver-bowel			No

Name of author, year of publication	Centre & country	Years from → to	RoB score	N	Inclusion criteria/ definition	Person-years of follow-up	Mortality (n, %) & causes (%)	IFALD (n, %)	CRBSI	Enteral autonomy (n, %)	Transplants (n, %) & type (%)	CVC, (n)	Neuro outcomes & other comments	Ultra (Yes/No)
Zamvar, V., et al., 2014(130)	Multi	2008 --> 2012	4	34	On HPN for IF with >2yrs follow-up	136.2	8 (24); 62.5% unknown		3.7	11 (32)	8 (24); 100% isolated small bowel			No
Gojda, J., et al., 2013(131)	Multi	1993 --> 2013	7	21	On HPN	76.3	2 (10); 100% unknown		1.1		0 (0);			No
Pironi, L., et al., 2011(132)	Multi	2004 --> 2008	6	166	HPN patient with 'benign' IF	830.0	11 (7); 27% liver failure, 45% unknown			50 (30)	12 (7); 100% isolated small bowel			No
Lambe, C., et al., 2018(133)	Necker, France	2008 --> 2014	7	195	On home PN	682.5	13 (7); 100% unknown		1.7	55 (28)	12 (6); 100% isolated small bowel	215		No
Petit, L. M., et al., 2016(134)	Necker, France	2000 --> 2009	7	151	On home PN (>3m PN and stable)	213.9	2 (1); 50% liver failure, 50% other			82 (54)	12 (8); 58% isolated small bowel, 42% combined liver-bowel			No
Nader, E. A., et al., 2015(135)	Necker, France	2000 --> 2013	6	251	On home PN (>3m PN and stable)	1506.0	24 (10); 8% liver failure, 63% sepsis, 29% other	68 (27)		131 (52)	19 (8); 74% isolated small bowel, 26% combined liver-bowel	384		No
Artru, S., et al., 2014(136)	Necker, France	1990 --> 2014	4	41	<30cm with ICV, or 40-80cm without ICV	574.0	14 (34);	14 (34)		5 (12)	24 (59); 63% isolated small bowel, 37% combined liver-bowel		BMI at end -1.14 +/- 1.15	Yes

Name of author, year of publication	Centre & country	Years from → to	RoB score	N	Inclusion criteria/ definition	Person-years of follow-up	Mortality (n, %) & causes (%)	IFALD (n, %)	CRBSI	Enteral autonomy (n, %)	Transplants (n, %) & type (%)	CVC, (n)	Neuro outcomes & other comments	Ultra (Yes/No)
Halac, U., et al., 2011(137)	Necker, France	1995 --> 2009	6	24	All with MVID	112.8	7 (29); 14% liver failure, 14% sepsis, 14% other, 57% unknown	20 (83)		7 (29)	13 (54); 69% isolated small bowel, 31% combined liver-bowel	110		No
Colomb, V., et al., 2007(138)	Necker, France	1980 --> 1999	6	302	On home PN (>3m PN and stable)	1792.3	48 (16); 13% liver failure, 10% sepsis, 71% other, 6% unknown	70 (23)	1.2	163 (54)	14 (5); 7% isolated liver, 43% isolated small bowel, 50% combined liver-bowel	326		No
Colomb, V., et al., 1992(139)	Necker, France	1980 --> 1992	5	43	PN >2yrs	140.4	1 (2); 100% liver failure	9 (21)			0 (0);			No
De Potter, S., et al., 1992(140)	Necker, France	1981 --> 1990	6	156	On home PN	156.0	29 (19); 3% liver failure, 7% sepsis, 83% other	7 (4)	1.1	49 (31)	0 (0);	179		No
Ricour, C., et al., 1990(141)	Necker, France	1981 --> 1988	6	112	On HPN	263.9	18 (16); 17% sepsis, 83% other	19 (17)	1.68	49 (44)	0 (0);	140		No
Ricour, C., et al., 1985(142)	Necker, France	1977 --> 1982	3	13	Extensive SB resection	27.0	0 (0)			7 (54)	0 (0);	42	normal weight and height	No
Schmidt-Sommerfeld, E., et al., 1990(143)	New York, USA	1980 --> 1985	6	35	On HPN	72.7	1 (3); 100% liver failure		4	29 (83)	0 (0);	101		No
Rhoads, J. M., et al., 2005(144)	North Carolina, USA	2002 --> 2005	6	24	PN>2wks	38.6	4 (17); 100% liver failure			16 (67)	0 (0);			No

Name of author, year of publication	Centre & country	Years from → to	RoB score	N	Inclusion criteria/ definition	Person-years of follow-up	Mortality (n, %) & causes (%)	IFALD (n, %)	CRBSI	Enteral autonomy (n, %)	Transplants (n, %) & type (%)	CVC, (n)	Neuro outcomes & other comments	Ultra (Yes/No)
Pharaon, I., et al., 1994(145)	Paris (Robertson), France	1985 --> 1994	5	20	IF	98.0	0 (0)	14 (70)	1.4		0 (0);	58		No
Neelis, E., et al., 2018(146)	Rotterdam, Netherlands	2001 --> 2015	6	52	>70% bowel resection	176.8	3 (6); 100% unknown			37 (71)	0 (0);		Height for age reduced after weaning.	No
Choi, S. J., et al., 2016(147)	Seoul, Korea	2001 --> 2014	5	19	PN >6m	73.3	3 (16); 33% sepsis, 67% other	11 (58)	2.7	2 (11)	1 (5); 100% combined liver-bowel			No
Brown, SK., et al., 2018(148)	Southampton, UK	2001 --> 2016	5	28	On home PN	168.0	3 (11); 67% liver failure	4 (14)	2.7	10 (36)	1 (4); 100% combined liver-bowel			No
Wiskin, A. E., et al., 2012(149)	Southampton, UK	2001 --> 2011	8	19	PN >28d	33.8	3 (16); 33% sepsis, 67% other	2 (11)	4	6 (32)	1 (5); 100% isolated small bowel	75		No
Pieroni, K. P., et al., 2013(150)	Stanford, Lucile-Packard, USA	2007 --> 2011	5	14	On HPN	67.1	0 (0)		2.7		0 (0);	46		No
Tung, Y. C., et al., 2006(151)	Taipei, Taiwan	1987 --> 2002	5	27	PN >2 months	52.9	6 (22); 83% sepsis, 17% other	13 (48)	3	14 (52)	0 (0);	83		No
Candusso, M., et al., 1997(152)	Trieste, Italy	1985 --> 1997	6	23	PN > 3 months	97.9	5 (22); 40% liver failure, 60% other		1.39	7 (30)	0 (0);	108		No
Moukarzel, A. A., et al., 1994(153)	UCLA, USA	1979 --> 1994	6	27	>5yrs HPN	229.5	0 (0)	7 (26)	1.13		0 (0);	123		No
Vargas, J. H., et al., 1987(154)	UCLA, USA	1977 --> 1987	6	102	PN > 3mths	201.5	31 (30); 6% liver failure, 29% sepsis, 65% other		1.29	51 (50)	0 (0);	234	1 at special school but rest at mainstream/graduated and one at school for gifted	No

Name of author, year of publication	Centre & country	Years from → to	RoB score	N	Inclusion criteria/ definition	Person-years of follow-up	Mortality (n, %) & causes (%)	IFALD (n, %)	CRBSI	Enteral autonomy (n, %)	Transplants (n, %) & type (%)	CVC, (n)	Neuro outcomes & other comments	Ultra (Yes/No)
Dorney, S. F., et al., 1985(155)	UCLA, USA	1977 --> 1984	5	13	<38cm jejunoileum	30.7	4 (31); 50% sepsis, 25% other, 25% unknown	12 (92)	1.54	5 (38)	0 (0);	27	weight: 7 children percentile improved, 2 stayed the same; height: 6 children percentile improved or stayed the same	Yes
Olszewska, K., et al., 2018(156)	Warsaw, Poland	2000 --> 2014	6	17	Ultra-short bowel syndrome	17.0	0 (0)	2 (12)		0 (0)	0 (0);			Yes
Friedman-Gruszczynska, J., et al., 2013(157)	Warsaw, Poland	2008 --> 2010	5	181	None, assume established IF if pt on HPN	543.0	1 (1); 100% sepsis		0.99	57 (31)				No
Ksiazyk, J., et al., 1999(158)	Warsaw, Poland	1992 --> 1997	5	25	On home PN	27.9	6 (24); 33% liver failure, 17% sepsis, 50% other	6 (24)	2.18	8 (32)	0 (0);	83		No
Nusinovich, Y., et al., 2013(159)	Washington, USA	2000 --> 2010	3	20	Intestinal atresias only	41.5	4 (20); 100% liver failure	17 (85)		11 (55)	2 (10); 100% combined liver-bowel			No

Name of author, year of publication	Centre & country	Years from → to	RoB score	N	Inclusion criteria/ definition	Person-years of follow-up	Mortality (n, %) & causes (%)	IFALD (n, %)	CRBSI	Enteral autonomy (n, %)	Transplants (n, %) & type (%)	CVC (n)	Neuro outcomes & other comments
Gupte, G. L., et al., 2007(160)	Birmingham, UK	1989 --> 2003	6	152	Referred for ITx assessment	631.5	37 (24); 5% sepsis, 54% unknown	100 (66)		18 (12)	33 (22); 27% isolated liver, 58% isolated small bowel, 15% combined liver-bowel		
Kelly, D., 2002(161)	Birmingham, UK	1989 --> 2000	5	80	Referred for ITx assessment	80.0	42 (53); 74% unknown				17 (21); 100% combined liver-bowel		
Beath, S. V., et al., 1995(162)	Birmingham, UK	1989 --> 1994	5	22	Chronic intestinal failure	22.0	14 (64); 71% liver failure, 7% sepsis, 21% other	12 (55)			2 (9); 100% combined liver-bowel		
Varkey, J., et al., 2013(163)	Gothenberg, Sweden	1998 --> 2011	4	19	Referred for ITx assessment	57.6	5 (26); 80% liver faiure, 60% sepsis	7 (37)			5 (26); 60% isolated small bowel, 40% combined liver bowel		
Chapman, B., et al., 2016(164)	Melbourne, Australia	2009 --> 2014	5	22	IF patients referred for ITx assessment	86.4	0 (0)			2 (9)	2 (9); 100% combined liver-bowel		
Torres, C., et al., 2007(165)	Nebraska, USA	2001 --> 2005	8	51	IRP enrolment (multiple indications)	60.5	5 (10); 60% sepsis, 20% unknown	36 (71)		31 (61)	10 (20); 30% isolated small bowel, 70% combined liver-bowel		
Ganousse-Mazeron, S., et al., 2015(166)	Necker, France	1995 --> 2008	7	118	ITx referral	885.0	33 (28); 48% liver failure, 30% sepsis, 21% other	41 (35)		10 (8)	62 (53); 52% isolated small bowel, 48% combined liver-bowel	6<br>1<br>4	

Name of author, year of publication	Centre & country	Years from → to	RoB score	N	Inclusion criteria/ definition	Person-years of follow-up	Mortality (n, %) & causes (%)	IFALD (n, %)	CRBSI	Enteral autonomy (n, %)	Transplants (n, %) & type (%)	CVC (n)	Neuro outcomes & other comments
Ruemmele, F. M., et al., 2004(167)	Necker, France	1995 --> 2003	7	12	MVID assessed for ITx	36.0	4 (33); 50% liver failure, 25% sepsis, 25% other			6 (50)	7 (58); 43% isolated small bowel, 57% combined liver-bowel		
Hugar, L. A., et al., 2018(168)	Pittsburgh, USA	2002 --> 2016	6	26	Clinical or genetic diagnosis of MMIHS, + IF diagnosis (definition = lack of functional intestinal mass needed for nutrient absorption and growth)	111.8	0 (0)				10 (38); 50% isolated small bowel, 50% combined liver-bowel		
Sigurdsson, L., et al., 1999(169)	Pittsburgh, USA	1989 --> 1997	6	27	CIPO and assessed for ITx	64.1	11 (41); 45% liver failure, 18% sepsis, 27% other, 9% unknown	14 (52)		1 (4)	8 (30); 13% isolated small bowel, 88% combined liver-bowel		
Avitzur, Y., et al., 2015(170)	Toronto, Canada	2003 --> 2005	5	18	need for PN treatment for >42 days or bowel length<25% of the predicted length for age at the time of primary surgery	53.4	12 (67); 41% liver failure, 33% sepsis, 25% other	12 (67)		4 (22)	5 (28); 100% isolated liver		
Avitzur, Y., et al., 2015(170)	Toronto, Canada	2006 --> 2009	5	33	need for PN treatment for >42 days or bowel length <25% of predicted length for age at the time of primary surgery	97.9	8 (24); 13% liver failure, 88% other	19 (58)		13 (29)	6 (18); 100% isolated liver		
Avitzur, Y., et al., 2015(170)	Toronto, Canada	1999 --> 2002	5	33	need for PN treatment for >42 days or bowel length <25% of	97.9	26 (79); 62% liver failure, 12% sepsis,	19 (58)		3 (9)	8 (24); 100% isolated liver		

Name of author, year of publication	Centre & country	Years from → to	RoB score	N	Inclusion criteria/ definition	Person-years of follow-up	Mortality (n, %) & causes (%)	IFALD (n, %)	CRBSI	Enteral autonomy (n, %)	Transplants (n, %) & type (%)	CVC (n)	Neuro outcomes & other comments
					predicted length for age at the time of primary surgery		27% other						
Fecteau, A., et al., 2001(171)	Toronto, Canada	1993 --> 1999	6	20	Referred for ITx assessment	41.5	7 (35); 43% sepsis, 57% other	14 (70)			2 (10); 100% combined liver-bowel		

Name of author, year of publication	Centre & country	Years from → to	RoB score	N	Inclusion criteria/ definition	Person-years of follow-up	Mortality (n, %) & causes (%)	IFALD (n, %)	CRBSI	Enteral autonomy (n, %)	Transplants (n, %) & type (%)	Lines (n)	Neuro outcomes & other comments
Garnett, G. M., et al., 2014(172)	Boston, USA	2004 --> 2012	7	15	STEP	33.8	1 (7); 100% liver failure	2 (13)		3 (20)	1 (7); 100% combined liver-bowel		
Ching, Y. A., et al., 2009(173)	Boston, USA	2002 --> 2008	7	16	STEP	30.7	0 (0)			6 (38)	2 (13); 100% combined liver-bowel		
Bishay, M., et al., 2012(174)	GOSH, UK	2006 --> 2010	7	87	Infants with PN > 28d	174.0	13 (15); 8% liver failure, 62% sepsis, 31% other	29 (33)		61 (70)	0 (0);		
Reinshagen, K., et al., 2008(175)	Heidelberg, Germany	1987 --> 2007	6	55	ILT	383.9	12 (22); 33% liver failure, 25% sepsis, 25% other, 17% unknown	20 (36)		36 (65)	0 (0);		at end follow up 31 patients increased weight percentile
Hosie, S., et al., 2006(176)	Heidelberg, Germany	1982 --> 2006	5	49	AGIR	87.2	9 (18); 44% liver failure, 33% sepsis, 22% other			19 (39)	0 (0);		

Name of author, year of publication	Centre & country	Years from →to	RoB score	N	Inclusion criteria/ definition	Person-years of follow-up	Mortality (n, %) & causes (%)	IFALD (n, %)	CRBSI	Enteral autonomy (n, %)	Transplants (n, %) & type (%)	Lines (n)	Neuro outcomes & other comments
Waag, K. L., et al., 1999(177)	Heidelberg, Germany	1984 --> 1999	6	25	>6m >50% PN	150.0	7 (28); 43% liver failure, 29% sepsis, 29% other			17 (68)	0 (0);		
Hukkinen, M., et al., 2015(178)	Helsinki, Finland	1994 --> 2014	8	21	AGIR	69.3	2 (10); 50% sepsis, 50% other	2 (10)		15 (71)	3 (14); 100% isolated small bowel		
Dore, M., et al., 2016(179)	Madrid, Spain	1995 --> 2016	7	22	SBS undergoing surgical techniques	113.7	4 (18); 100% unknown	21 (95)		10 (45)	5 (23); 100% isolated small bowel		
Pederiva, F., et al., 2018(180)	Manchester, UK	2002 --> 2012	5	43	SBS undergoing AGIR	159.1	2 (5); 50% sepsis, 50% other	0 (0)		25 (58)	0 (0);		
Bianchi, A., 1997(181)	Manchester, UK	1982 --> 1997	6	20	SBS undergoing LILT	136.0	11 (55); 91% liver failure, 9% sepsis			7 (35)	0 (0);		
Barrett, M., et al., 2017(182)	Michigan, USA	2003 --> 2014	7	17	STEP	51.0	1 (6); 100% other			4 (24)	0 (0);		
Miyasaka, E. A., et al., 2011(183)	Michigan, USA	1995 --> 2009	5	14	SBS with ILP	79.8	2 (14); 50% sepsis, 50% other			6 (43)	1 (7); 100% isolated small bowel		
Weber, T. R., 1999(184)	Missouri, USA	1991 --> 1999	6	16	SBS: <100cm bowel & isoperistaltic lengthening	16.0	0 (0)			14 (88)	0 (0);		
Jones, B. A., et al., 2013(185)	Multi	2004 --> 2010	8	97	None, assume established IF if receiving STEP	153.5	11 (11); 100% unknown			51 (53)	5 (5); 100% isolated small bowel		
Wood, S. J., et al., 2013(186)	Multi	2004 --> 2010	5	14	Neonatal SBS undergoing AGIR	24.9	0 (0)				1 (7); 100% isolated liver	62	
Modi, B. P., et al., 2007(187)	Multi (Boston, USA)	2004 --> 2006	4	38	STEP	39.9	3 (8); 100% liver failure			11 (29)	3 (8); 100% combined liver-bowel		

Name of author, year of publication	Centre & country	Years from → to	RoB score	N	Inclusion criteria/ definition	Person-years of follow-up	Mortality (n, %) & causes (%)	IFALD (n, %)	CRBSI	Enteral autonomy (n, %)	Transplants (n, %) & type (%)	Lines (n)	Neuro outcomes & other comments
Mercer, D. F., et al., 2014(188)	Nebraska, USA	2006 --> 2011	5	51	STEP	165.8	3 (6); 33% liver failure, 33% sepsis, 33% other			29 (57)	7 (14); 100% isolated small bowel		
Andres, A. M., et al., 2008(189)	Nebraska, USA	2002 --> 2007	6	14	Repeat lengthening after previous Bianchi or STEP	16.9	0 (0)			9 (64)	4 (29); 75% isolated small bowel, 25% combined liver-bowel		
Sudan, D., et al., 2007(190)	Nebraska, USA	1982 --> 2007	5	50	ILT	190.0	5 (10); 100% liver failure				0 (0);		
Thompson, J. S., et al., 2000(191)	Nebraska, USA	1990 --> 2000	5	13	ILT	38.6	1 (8); 100% unknown			6 (46)	2 (15); 100% isolated small bowel		
Fujioka, W. K., et al., 2015(192)	New York, USA	2004 --> 2012	6	18	STEP	46.8	1 (6); 100% unknown		0.64	8 (44)	2 (11); 50% isolated liver, 50% isolated small bowel		
Oh, P. S., et al., 2014(193)	New York, USA	2004 --> 2011	7	15	STEP	25.5	0 (0)			5 (33)	1 (7); 100% isolated small bowel		
Walker, S. R., et al., 2006(194)	Pittsburgh, USA	1984 --> 2004	5	19	Bianchi	147.1	4 (21); 75% unknown			16 (84)	9 (47); 100% isolated small bowel		
Bueno, J., et al., 2001(195)	Pittsburgh, USA	1990 --> 2001	8	27	LILT for IF and TPN dependency	65.7	9 (33); 100% liver failure	16 (59)			14 (52); 50% isolated small bowel, 50% combined liver-bowel		
Javid, P. J., et al., 2013(196)	Seattle, USA	2004 --> 2011	5	16	All STEPs	34.7	2 (13); 50% liver failure, 50% sepsis	2 (13)		10 (63)	2 (13); 100% isolated small bowel		

Name of author, year of publication	Centre & country	Years from → to	RoB score	N	Inclusion criteria/ definition	Person-years of follow-up	Mortality (n, %) & causes (%)	IFALD (n, %)	CRBSI	Enteral autonomy (n, %)	Transplants (n, %) & type (%)	Lines (n)	Neuro outcomes & other comments
Wester, T., et al., 2014(197)	Stockholm, Sweden	2004 --> 2015	6	27	STEP	101.5	1 (4); 100% sepsis	6 (22)		16 (59)	0 (0);		
Fitzgerald, K. et al., 2018(198)	Toronto, Canada	2003 --> 2005	6	12	STEP	47	3 (33); 100% liver failure			7 (58)	2 (17); 50% isolated small bowel, 50% combined liver-bowel		
Fitzgerald, K. et al., 2018(198)	Toronto, Canada	2006 --> 2016	6	24	STEP	102	1 (4); 100% liver failure			8 (33)	4 (17); 50% isolated liver, 50% combined liver-bowel		
Oliveira, C., et al., 2012(199)	Toronto, Canada	2003 --> 2005	6	12	STEP	68.0	2 (17); 100% liver failure	5 (42)		7 (58)	2 (17); 100% combined liver-bowel		
Wales, P. W., et al., 2007(200)	Toronto, Canada	2003 --> 2006	7	14	STEP	26.8	3 (21); 67% liver failure, 33% other	6 (43)		7 (50)	2 (14); 100% combined liver-bowel		
Sehgal, S., et al., 2018(201)	Washington, USA	2007 --> 2013	4	16	>4wk PN	32.0	0 (0)	14 (88)	15.1		0 (0);		
Shah, A. A., et al., 2019(202)	Washington, USA	2004 --> 2014	5	22	Extreme short bowel 10-35cm without IFALD; raised bilirubin and >35cm bowel, or advanced IFALD with >50cm	78.8	0 (0)			11 (50)	1 (5); 100% isolated small bowel		

Characteristics of studies included in systematic review. Unique studies are those without overlapping cohorts (based on years and centres of inclusion) and were included in meta-analysis. Risk of bias (RoB) score is an eight point score (0-8) from the Cochrane Risk of Bias Tool for Cohort Studies, where a score of 8 would represent the lowest risk of bias. CRBSI, catheter-related blood stream infections per 1000 catheter days; CVC, total number of central venous catheters used; GA, gestational age; IFALD, intestinal failure associated liver disease; ILP, intestinal lengthening procedure; LILT, longitudinal intestinal lengthening and tailoring procedure; STEP, serial transverse enteroplasty procedure; PN, parenteral nutrition; SBS, short bowel syndrome; TPN, total (exclusive) parenteral nutrition.

Supplemental table 3: Meta-regression for heterogeneity in total mortality

Variable	Beta co-efficient	95% CI	P-value
Proportion with IFALD	.072083	(.027,.118)	.002
CRBSI per 1000 catheter days	.007376	(.003,.012)	.003
Proportion with liver failure	.099588	(.022,.177)	.013
Year follow-up ended	-.00181	(-.003,-.0003)	.018
Proportion with NEC	.04556	(-.019,-.110)	.167
Age at the start of follow-up	-.00024	(-.001,.0002)	.280
Proportion with ileocecal valve	.020014	(-.032,.072)	.450
Gestational age at birth	-.00369	(-.015,.008)	.516
Birth weight	-.0000229	(-.0001,.0001)	.557
Proportion with short bowel syndrome	.005137	(-.024,.034)	.726
Proportion due to neuromuscular disorder	.012369	(-.064,.088)	.748
Small bowel length	-.00011	(-.001,.001)	.786
Year follow-up started	.000138	(-.001,.001)	.812
Proportion male	-.00667	(-.104,.091)	.892
Quality score	.000114	(-.009,.009)	.980
Mean duration of PN	-.0000173	(-.00005,.00001)	.273

Supplemental table 4: Comparison of clinical outcomes for cohorts separated by era (early, middle, & late)

Variable	Early (pre-2000)	Middle (2000-2005)	Late (post-2005)
CRBSI (per 1000 catheter days) (95% CI)	2.8 (2.7,2.9)	3.3 (3.2,3.4)	3.9 (3.7,4.1)
Total mortality (%/year) (95% CI)	5.9 (4.2,7.9)	5.1 (4.0,6.2)	4.5 (3.6,6.7)
Liver failure (%/year) (95% CI)	3.8 (2.4,5.4)	5.0 (3.7,6.5)	3.3 (1.6,5.4)
Enteral autonomy (%/year) (95% CI)	22 (16,28)	16 (14,19)	30 (21,40)

Comparison of outcomes by meta-analysis when studies were grouped such that the “Early” group had follow-up completed before 2000, “Middle” group had follow-up that started after 2000 but before 2005, and “Late” were cohorts where follow-up began after 2005. CRBSI, catheter-related blood stream infection.

Supplemental table 5: Meta-regression for heterogeneity in enteral autonomy

Variable	Beta co-efficient	95% CI	P-value
Gestational age at birth	-.05544	(-.074,.037)	$2.4x10^{-7}$
Proportion with NEC	.36252	(.206,.519)	$1.2x10^{-5}$
CRBSI per 1000 catheter days	.017392	(.009,.026)	$1.0x10^{-4}$
Small bowel length	.002631	(.001,.004)	$2.5x10^{-4}$
Mean duration of PN	-.00014	(-.0002,-.0001)	.003
Birth weight	-.00023	(-.0004,-.0001)	.006
Proportion due to neuromuscular disorder	-.25179	(-.438,-.065)	.009
Year follow-up started	.003195	(.0003,.006)	.030
Proportion with IFALD	.133811	(.008,.260)	.038
Proportion with liver failure	-.13524	(-.315,.044)	.138
Proportion male	-.19656	(-.468,.075)	.153
Quality score	-.01782	(-.043,.007)	.162
Year follow-up ended	-.00194	(-.006,.002)	.333
Proportion with ileocecal valve	.065639	(-.079,.210)	.369
Age at the start of follow-up	-.00056	(-.002,.001)	.410
Proportion with short bowel syndrome	-.02696	(-.100,.046)	.468

Supplemental table 6: A Sub-analysis for cohorts of ultra-Short Bowel Syndrome (SBS) against those without ultra-SBS

Variable		
	**Ultra-SBS**	**Non-ultra SBS**
Age at start of f/u (months) (95% CI)	0.7 (0.6,0.8)	6.3 (6.0,6.4)
Male (%) (95% CI)	71.5 (71.1,71.8)	58 (57,0.59)
Gestational age (weeks) (95% CI)	36.2 (36,36.4)	34 (34,34.1)
Birth weight (g) (95% CI)	No data	2190 (2182,2197)
Short bowel syndrome (%) (95% CI)	76.4 (73.6,79.3)	44 (0.43,0.44)
Necrotizing enterocolitis (%) (95% CI)	9.7 (9.2,10.1)	25 (0.24,0.25)
Neuro-muscular disorder (%) (95% CI)	0.7 (0.6,0.9)	9.9 (0.09,0.1)
Small bowel length (cm) (95% CI)	16.1 (15.9,16.4)	47.2 (46.8,47.6)
Ileocecal valve (%) (95% CI)	40.4 (38.1,42.6)	49 (0.48,0.49)
Duration of PN (days) (95% CI)	No data	395 (389,401)
Time to enteral autonomy (days) (95% CI)	2309 (2236,2382)	284 (281,288)
Height at start of f/u (z-score) (95% CI)	-2.9 (-2.9,-2.9)	No data
Height at end of f/u (z-score) (95% CI)	-0.52 (-0.5,-0.5)	-1.50 (-1.51,-1.47)
Weight at start of f/u (z-score) (95% CI)	-2.1 (-2.1,-2.1)	-1.9 (-2.04,-1.76)
Weight at end of f/u (z-score) (95% CI)	-0.99 (-1,-1)	-1.10 (-1.14,-1.06)
CRBSI (per 1000 catheter days) (95% CI)	3.0 (3.0,3.0)	5.8 (5.6,5.9)
Total mortality (%/year) (95% CI)	3.9 (0.3,10.7)	5.6 (4.4,7.0)
IFALD (%/year) (95% CI)	14 (1.4,35)	20 (15,26)
Liver failure (%/year) (95% CI)	5.1 (0.3,14)	3.2 (2.3,4.2)
Transplant (%/year) (95% CI)	8.4 (1.9,18.5)	0.4 (0.1,0.9)
Enteral autonomy (%/year) (95% CI)	10.4 (3.1,21)	25 (21,30)
Thrombosis (%/year) (95% CI)	2.2 (1.2,3.8)	8.1 (3.1,15.1)

Supplemental table 7: Meta-regression for heterogeneity in IFALD

Variable	Beta co-efficient	95% CI	P-value
CRBSI per 1000 catheter days	.020	(.013,.026)	$1.7x10^{-7}$
Proportion with NEC	.380	(.218,.543)	$1.2x10^{-5}$
Gestational age at birth	-.049	(-.075,-.023)	$5.5x10^{-4}$
Birth weight	-.0003	(-.001,-.0001)	0.002
Year follow-up started	.005	(.002,.008)	0.003
Small bowel length	.002	(.0003,.004)	0.026
Proportion with ileocecal valve	.163	(-.050,.375)	0.131
Year follow-up ended	-.003	(-.007,.001)	0.132
Proportion male	-.327	(-.758,.104)	0.134
Age at the start of follow-up	-.001	(-.003,.0004)	0.135
Proportion with short bowel syndrome	-.056	(-.140,.027)	0.184
Quality score	-.012	(-.039,.014)	0.355
Proportion due to neuromuscular disorder	-.058	(-.238,.123)	0.528
Mean duration of PN	-.0001	(0.0001,.00002)	0.151

Supplemental table 8: Frequency of reporting of baseline characteristics in included cohorts

Baseline characteristic	Proportion of cohorts reporting
Sex	60% (132/220)
Ethnicity	7% (15/220)
Aetiology	90% (197/220)
Gestational age	42% (92/220)
Birth weight	31% (69/220)
Small bowel length	58% (127/220)
Presence of ICV	47% (103/220)

Supplemental table 9: Definitions used by cohorts included in the systematic review

**Definitions of intestinal failure (with or without short bowel syndrome)**	**Proportion of cohorts reporting**
42d PN, or <25% bowel length	20.5% (17/83)
3m PN	15.7% (13/83)
1m PN	7.2% (6/83)
2m PN	4.8% (4/83)
42d PN	4.8% (4/83)
2m PN or <50% bowel length	3.6% (3/83)
42d PN or <30% bowel length	3.6% (3/83)
6m PN or <25% bowel length	2.4% (2/83)
3m PN or <25% bowel length	2.4% (2/83)
3m PN, or <50% bowel length	2.4% (2/83)
2m of at least 50% PN, or <50cm in pre-term, or <75cm in term	2.4% (2/83)
2m PN, or <40cm bowel length	2.4% (2/83)
2m of at least 50% PN	2.4% (2/83)
<100cm bowel length	2.4% (2/83)
<50% bowel length	2.4% (2/83)
<75cm bowel length	2.4% (2/83)
<25% bowel length	2.4% (2/83)
2wk PN	2.4% (2/83)
6m PN	1.2% (1/83)
3m PN, or <30% bowel length	1.2% (1/83)
42d PN, or <40cm bowel length	1.2% (1/83)
2m PN, or 70cm bowel length	1.2% (1/83)
1m (100% PN) or 3m (partial PN)	1.2% (1/83)
1m (>75% PN) or 3m (>50% PN)	1.2% (1/83)
<180cm bowel length	1.2% (1/83)
<60cm bowel length	1.2% (1/83)
<30% bowel length	1.2% (1/83)
40cm bowel length	1.2% (1/83)
Lack of functional intestinal mass needed for nutrient absorption and growth	1.2% (1/83)

**Duration of PN in definitions of intestinal failure (excluding HPN)**	**Proportion of cohorts reporting**
42 days	27.1% (22/81)
3 months	25.9% (21/81)
2 months	18.5% (15/81)
1 month	17.3% (14/81)
6 months	4.9% (4/81)
2 weeks	4.9% (4/81)
3 weeks	1.2% (1/81)
**Definitions of bowel length for short bowel syndrome**	**Proportion of cohorts reporting**
<25%	43.1% (22/51)
<50%	15.7% (8/51)
<30%	13.7% (7/51)
<40cm	7.8% (4/51)
<100cm	5.9% (3/51)
<75cm	3.9% (2/51)
<50cm (term) / <75cm (pre-term)	3.9% (2/51)
<120cm	2.0% (1/51)
<70cm	2.0% (1/51)
<60cm	2.0% (1/51)
**Definitions of ultra-short bowel syndrome**	**Proportion of cohorts reporting**
<20cm	33.3% (3/9)
<10cm	22.2% (2/9)
<30cm with ICV, or <40-80cm without ICV	11.1% (1/9)
<38cm	11.1% (1/9)
<30%	11.1% (1/9)
<20% residual gut and >90 days PN	11.1% (1/9)

**Definitions of IFALD**	**Proportion of cohorts reporting**
Bilirubin >2mg/dL for >2wk	25% (3/12)
Bilirubin >2mg/dL	16.7% (2/12)
Bilirubin >2mg/dL for >3wk	8.3% (1/12)
Bilirubin >2mg/dL for >6wk and ALT >2x ULN	8.3% (1/12)
Bilirubin >2mg/dL, or abnormal biopsy	8.3% (1/12)
Bilirubin >4mg/dL	8.3% (1/12)
Child's stage B or C	8.3% (1/12)
Cholestasis for >6wk	8.3% (1/12)
Abnormal LFTs	8.3% (1/12)

Supplemental table 10: Summary of previously published systematic reviews and meta-analyses

Author and year	Systemic review or meta-analysis	Topic/question	Number of studies included		Number of patients included		Key findings
			SR	MA	SR	MA	
Stanger et al (2013)(203)	Systematic review and meta-analysis	Impact of multi-disciplinary intestinal rehabilitation programs on the outcome of pediatric IF patients	15	3	150 3	130	IRP reduce CRBSI and overall survival, compared to historical controls. Weaning from PN, IFALD, and risk of liver transplant were reduced but did not reach statistical significance.
Frongia et al (2013)(204)	Systematic review	Comparison of LILT and STEP procedures in children with SBS	39	N/A	472	N/A	Both are indicated for a failure to achieve enteral autonomy, whilst end-stage liver disease is a contraindication. Both need a dilated intestinal segment but STEP can be performed on the duodenum, which is not possible for LILT. There is similar lengthening (~70%), increase in enteral tolerance, and reduced cholestasis for both but STEP seems to have lower mortality and transplantation rates.
Oliveira et al (2012)(205)	Meta-analysis	Ethanol Locks to Prevent CRSBIs in PN	N/A	4	N/A	53	Ethanol lock (EL) was found to be more effective than heparin lock (HL) with a 81% reduction and CRBSI and 72% reduction in CVC replacement. 108-150 catheter days of EL were needed to prevent 1 CRBSI and 122-689 days to avoid 1 catheter replacement. Thrombotic events were uncommon.
Lauriti et al (2014)(206)	Systematic review	Incidence, Prevention, and Treatment of PNAC and IFALD in Infants and Children	23	N/A	328 0	N/A	Incidence of PNAC was 29% and IFLAD was 50% for children having >14 days PN, without change in the last two decades. Incidence of PNAC increased from <30 days PN, to 30-60 days PN, to>60 days PN. Efficacious prevention strategies in preterm neonates include oral erythromycin and amino-acid free PN with enteral whey protein.
Nakamura et al (2017)(207)	Meta-analysis	Clinical outcome of ITx in patients with total intestinal aganglionosis	N/A	13	N/A	63	35% had isolated ITx with 67% having multivisceral transplant. After a mean follow-up 40months, survival was 66% with the oldest survivor 13 years old. ITx appears to be a feasible treatment option.
Belza et al (2017)(208)	Systematic review	Impact of multidisciplinary teams for management of IF	4	N/A	383	N/A	Similar to findings from Stanger et al. (2013), IRP improve survival, enteral autonomy, reduce CVC use, reduce transplantation, and reduced end-stage liver disease.
Fernandes et al (2016)(209)	Systematic review and meta-analysis	Improved enteral tolerance in children following STEP procedure	8	7	183	86	After STEP, mean enteral tolerance increased from 35% to 70%. 87% (but not all) children improved after STEP.

Author and year	Systemic review or meta-analysis	Topic/question	Number of studies included		Number of patients included		Key findings
			SR	MA	SR	MA	
Reddy et al (2013)(210)	Systematic review	Role of probiotics in SBS in infants and children	11	N/A	30	N/A	Insufficient data to support the use of probiotics. A crossover RCT did not show improved intestinal permeabilitly and other studies were either case reports or case series that did not demonstrate improved clinical outcomes.
King et al (2013)(211)	Systematic review	Patient outcomes of intestinal bowel lengthening (STEP and Bianchi) in children with SBS	33	N/A	403	N/A	Survival post-procedure is 89% in the last 15 years and appears to be improving. Bianchi procedure has a higher rate of weaning but also with increased transplantation. STEP has higher complication rate. There was different follow-up durations between procedures, which may impact results.
Duran (2005)(212)	Systematic review	The effects of long-term TPN on gut mucosal immunity in children with SBS	13	N/A	414	N/A	There was insufficient evidence to suggest that TPN causes deleterious effects on the gut e.g. villous atrophy, impaired neutrophil function. The evidence reviewed suggested that enteral nutrition was no safer than TPN.
Bergholz et al (2014)(213)	Systematic review and meta-analysis	Comparison of the outcome of newborns with simple (sGS) and complex gastroschisis (cGS: gastroschisis with atresia, necrosis, perforation or volvulus)	13	13	130 1	1301	cGS has significantly greater mortality (17%) than sGC (2.2%). cGS is associated with later initiation of enteral feeds, longer duration of PN, and are more likely to have sepsis, NEC, and SBS, as well as go home on PN.
Barclay et al (2011)(214)	Systematic review	Medical and nutritional interventions for management of IF and its resultant complications in children	33	N/A	No dat a	N/A	There is limited quality evidence for any medical/nutritional intervention assessed. Growth hormone & glutamine have not been assessed by RCT. Ursodeoxycholic acid improves bilirubin but its effect on the liver long-term is not clear. Hydrolysed formulae and elemental feeds have not been studied by RCT. There is some benefit for fish oil and olive oil PN, plus uncontrolled evidence for lipid cycling. There isn’t sufficient evidence to support use of metronidazole prophylactically.
Rangel et al (2012)(215)	Systematic review	PNAC and PNALD in children	88	N/A	N/A	N/A	Similar results to those of Lauriti et al. (2014). NEC & PN duration as significant risk factors but unclear about prematurity. Fish oil lipid helps reduce PNALD and trophic feeding is of benefit. Some evidence to suggest benefit from oral bile acids or erythromycin.

Author and year	Systemic review or meta-analysis	Topic/question	Number of studies included		Number of patients included		Key findings
			SR	MA	SR	MA	
Hau et al (2018)(216)	Systematic review and meta-analysis	GI sequelae after surgery for NEC	58	42	4260	No data	Common to have GI complications after surgery for NEC, including: strictures (24%), recurrence (8%), IF (13%), and adhesional ileus (6%). Strictures were most common after enterostomy without bowel resection. Significant study heterogeneity may have limited findings.
Richards et al (1997)(217)	Systematic review	Effectiveness of HPN	65	N/A	No data	N/A	A broad systematic review that also covered adults on HPN, though the majority of patients were young. Most morbidity is related to the CVC. The outcome for adults is poor, particularly in the context of malignancy.
Dicken et al (2011)(218)	Systematic review	Medical management of motility disorders in patients with IF: a focus on NEC, gastroschisis, and intestinal atresia	92	N/A	N/A	N/A	Overall, there was limited data for any intervention. Loperamide reduces stool volumes and slows transit times but does not improve absorption. Dysmotile patients are at risk of carbohydrate fermentation and lactic acidosis with absorbents (pectin or guar gum). Cholestyramine may be used for patients with bile acid diarrhoea and an intact colon.
Seida et al (2013)(219)	Systematic review	Benefits and safety of parenteral ω-3 fatty acid lipid emulsions for children with IF and other conditions	8	N/A		N/A	ω-3 fatty acid lipid emulsions improve IFLAD biochemistry but have no impact on mortality. There is very limited evidence for any other clinical outcomes so long-term studies needed into benefit & harms.
Rahhal et al (2018)(220)	Systematic review and meta-analysis	Effectiveness and safety of ethanol locks in pediatric patients with IF as compared with standard heparin locks	9	147	9	147	Consistent with the earlier review by Oliveira et al. (2012), ethanol locks reduced CRBSI by 63% (mean difference 6.3 CRBSI per 1000 catheter days) and reduced need for replacement. However heparin locks were associated with reduced need for catheter repair.

cGS, complex gastroschisis; CRBSI, catheter-related blood stream infections per 1000 catheter days; CVC, total number of central venous catheters used; EL, ethanol lock; GA, gestational age; HL, heparin lock; IFALD, intestinal failure associated liver disease; ILP, intestinal lengthening procedure; IRP, intestinal rehabilitation programmes; LILT, longitudinal intestinal lengthening and tailoring procedure; MA, meta-analysis; PN, parenteral nutrition; PNAC, PN-associated cholestasis; SBS, short bowel syndrome; sGS, simple gastroschisis; SR, systematic review; STEP, serial transverse enteroplasty procedure; TIA, total intestinal aganglionosis; TPN, total parenteral nutrition.

Supplemental references

1. Meehan JJ, Georgeson KE. Prevention of liver failure in parenteral nutrition-dependent children with short bowel syndrome. J Pediatr Surg. 1997;32:473–4.
2. Georgeson KE, Breaux CW. Outcome and intestinal adaptation in neonatal short-bowel syndrome. J Pediatr Surg. 1992;27:344–50.
3. Capriati T, Giorgio D, Fusaro F, Candusso M, Schingo P, Caldaro T, Laureti F, Elia D, Diamanti A. Pediatric Short Bowel Syndrome: Predicting Four-Year Outcome after Massive Neonatal Resection. Eur J Pediatr Surg Off J Austrian Assoc Pediatr Surg . [et al] = Zeitschrift fur Kinderchirurgie. United States; 2018;28:455–63.
4. Casaccia G, Trucchi A, Spirydakis I, Giorlandino C, Aite L, Capolupo I, Catalano OA, Bagolan P. Congenital intestinal anomalies, neonatal short bowel syndrome, and prenatal/neonatal counseling. J Pediatr Surg. 2006;41:804–7.
5. Diamanti A, Gambarara M, Knafelz D, Marcellini M, Boldrini R, Ferretti F, Papadatou B, Castro M. Prevalence of liver complications in pediatric patients on home parenteral nutrition: indications for intestinal or combined liver-intestinal transplantation. Transpl Proc. 2003/12/31. 2003;35:3047–9.
6. Gambarara M, Diamanti A, Castro M, Knafelz D, Ferretti F, Papadatou B, D'Orio F. Clinical outcome and etiology of chronic non-malignant intestinal failure: A pediatric series. Transplant Proc. 2002;34:3363–5.
7. Hong CR, Han SM, Staffa SJ, Carey AN, Modi BP, Jaksic T. Long-term outcomes of ultrashort bowel syndrome due to malrotation with midgut volvulus managed at an interdisciplinary pediatric intestinal rehabilitation center. J Pediatr Surg. United States; 2019;
8. Sparks EA, Khan FA, Fisher JG, Fullerton BS, Hall A, Raphael BP, Duggan C, Modi BP, Jaksic T. Necrotizing enterocolitis is associated with earlier achievement of enteral autonomy in children with short bowel syndrome. J Pediatr Surg. United States; 2016;51:92–5.
9. Fullerton BS, Sparks EA, Hall AM, Duggan C, Jaksic T, Modi BP. Enteral autonomy, cirrhosis, and long term transplant-free survival in pediatric intestinal failure patients. J Pediatr Surg. Elsevier Inc.; 2016;51:96–100.
10. Fallon EM, Mitchell PD, Nehra D, Potemkin AK, O'Loughlin AA, Gura KM, Puder M. Neonates with short bowel syndrome: An optimistic future for parenteral nutrition independence. JAMA Surg. 2014;149:663–70.
11. Modi BP, Langer M, Ching YA, Valim C, Waterford SD, Iglesias J, Duro D, Lo C, Jaksic T, Duggan C. Improved survival in a multidisciplinary short bowel syndrome program. J Pediatr Surg. United States; 2008;43:20–4.
12. Andorsky DJ, Lund DP, Lillehei CW, Jaksic T, DiCanzio J, Richardson DS, Collier SB, Lo C, Duggan C. Nutritional and other postoperative management of neonates with short bowel syndrome correlates with clinical outcomes. J Pediatr. 2001;139:27–33.
13. Busoni VB, Lobos PA, Ussher F, Izquierdo C, Frangi F, Claria RS, D'Agostino D, Orsi M. Improved outcome of pediatric intestinal failure with early referral to a multidisciplinary team. J Pediatr Gastroenterol Nutr. 63:S119.
14. Sigalet D, Boctor D, Robertson M, Lam V, Brindle M, Sarkhosh K, Driedger L, Sajedi M. Improved outcomes in paediatric intestinal failure with aggressive prevention of liver disease. Eur J Pediatr Surg Off J Austrian Assoc Pediatr Surg . [et al] = Zeitschrift fur Kinderchirurgie. United States; 2009;19:348–53.
15. Chaet MS, Farrell MK, Ziegler MM, Warner BW. Intensive nutritional support and remedial surgical intervention for extreme short bowel syndrome. J Pediatr Gastroenterol Nutr. United States; 1994;19:295–8.

16. Mousa H, Hyman PE, Cocjin J, Flores AF, Di Lorenzo C. Long-term outcome of congenital intestinal pseudoobstruction. Dig Dis Sci. 2002;47:2298–305.

17. Caniano DA, Starr J, Ginn-Pease ME. Extensive short-bowel syndrome in neonates: outcome in the 1980s. Surgery. United States; 1989;105:119–24.

18. Sondheimer JM, Cadnapaphornchai M, Sontag M, Zerbe GO. Predicting the duration of dependence on parenteral nutrition after neonatal intestinal resection. J Pediatr. United States; 1998;132:80–4.

19. Barclay AR, Paxton CE, Gillett P, Hoole D, Livingstone J, Young D, Menon G, Munro F, Wilson DC. Regionally acquired intestinal failure data suggest an underestimate in national service requirements. Arch Dis Child. 2009;94:938–43.

20. Robinson JL, Casey LM, Huynh HQ, Spady DW. Prospective Cohort Study of the Outcome of and Risk Factors for Intravascular Catheter-Related Bloodstream Infections in Children with Intestinal Failure. J Parenter Enter Nutr. 2014;38:625–30.

21. Casey L, Lee K-H, Rosychuk R, Turner J, Huynh HQ. 10-Year Review of Pediatric Intestinal Failure: Clinical Factors Associated With Outcome. Nutr Clin Pract. 2008;23:436–42.

22. Olieman JF, Poley MJ, Gischler SJ, Penning C, Escher JC, van den Hoonaard TL, van Goudoever JB, Bax NMA, Tibboel D, IJsselstijn H. Interdisciplinary management of infantile short bowel syndrome: resource consumption, growth, and nutrition. J Pediatr Surg. Elsevier Inc.; 2010;45:490–8.

23. Olieman JF, Tibboel D, Penning C. Growth and nutritional aspects of infantile short bowel syndrome for the past 2 decades. J Pediatr Surg. United States; 2008;43:2061–9.

24. Suita S, Masumoto K, Yamanouchi T, Nagano M, Nakamura M. Complications in neonates with short bowel syndrome and long-term parenteral nutrition. JPEN J Parenter Enteral Nutr. United States; 1999;23:S106-9.

25. Pichler J, Horn V, Macdonald S, Hill S. Intestinal failure-associated liver disease in hospitalised children. Arch Dis Child. England; 2012;97:211–4.

26. Heneyke S, Smith V V, Spitz L, Milla PJ. Chronic intestinal pseudo-obstruction: treatment and long term follow up of 44 patients. Arch Dis Child. 1999;81:21–7.

27. Dollery CM, Sullivan ID, Bauraind O, Bull C, Milla PJ. Thrombosis and embolism in parenteral nutrition long-term central venous access. Lancet. 1993;344:1043–5.

28. Mayr JM, Schober PH, Weissensteiner U, Hollwarth ME. Morbidity and mortality of the short-bowel syndrome. Eur J Pediatr Surg Off J Austrian Assoc Pediatr Surg . [et al] = Zeitschrift fur Kinderchirurgie. United States; 1999;9:231–5.

29. Schurink M, Hulscher JBF, Nieuwenhuijs VB, Heineman E, Rings EHHM. A surgical perspective of the outcome of a multidisciplinary intestinal rehabilitation program for children with short bowel syndrome in The Netherlands. Transplant Proc. United States; 2014;46:2102–8.

30. Merras-Salmio L, Mutanen A, Ylinen E, Rintala R, Koivusalo A, Pakarinen MP. Pediatric Intestinal Failure: The Key Outcomes for the First 100 Patients Treated in a National Tertiary Referral Center During 1984-2017. JPEN J Parenter Enteral Nutr. United States; 2018;

31. Hukkinen M, Kivisaari R, Merras-Salmio L, Koivusalo A, Pakarinen MP. Small Bowel Dilatation Predicts Prolonged Parenteral Nutrition and Decreased Survival in Pediatric Short Bowel Syndrome. Ann Surg. 2017;266:369–75.

32. Pakarinen MP, Pakkasjarvi N, Merras-Salmio L, Koivusalo A, Rintala R. Intestinal rehabilitation of infantile onset very short bowel syndrome. J Pediatr Surg. United States; 2015;50:289–92.

33. Merras-Salmio L, Pakarinen MP. Refined Multidisciplinary Protocol-Based Approach to Short Bowel Syndrome Improves Outcomes. J Pediatr Gastroenterol Nutr. United States; 2015;61:24–9.

34. Mutanen A, Koivusalo A, Pakarinen M. Complicated Gastroschisis Is Associated with Greater Intestinal Morbidity than Gastroschisis or Intestinal Atresia Alone. Eur J Pediatr Surg Off J Austrian Assoc Pediatr Surg . [et al] = Zeitschrift fur Kinderchirurgie. United States; 2018;28:495–501.

35. Pakarinen MP, Kurvinen A, Koivusalo AI, Ruuska T, Makisalo H, Jalanko H, Rintala RJ. Surgical treatment and outcomes of severe pediatric intestinal motility disorders requiring parenteral nutrition. J Pediatr Surg. United States; 2013;48:333–8.

36. Kurvinen A, Nissinen MJ, Andersson S, Korhonen P, Ruuska T, Taimisto M, Kalliomaki M, Lehtonen L, Sankilampi U, Arikoski P, et al. Parenteral plant sterols and intestinal failure-associated liver disease in neonates. J Pediatr Gastroenterol Nutr. United States; 2012;54:803–11.

37. Pakarinen MP, Koivusalo AI, Rintala RJ. Outcomes of intestinal failure--a comparison between children with short bowel and dysmotile intestine. J Pediatr Surg. United States; 2009;44:2139–44.

38. Pakarinen M, Halttunen J, Rintala R, Kuusanmaki P. Gut failure in pediatric and adult patients. Candidates for small-bowel transplantation in southern Finland. Scand J Gastroenterol. England; 1995;30:764–70.

39. Grosfeld JL, Rescorla FJ, West KW. Short bowel syndrome in infancy and childhood. Analysis of survival in 60 patients. Am J Surg. 1986;151:41–6.

40. Kaji T, Nakame K, Machigashira S, Kawano T, Masuya R, Yamada W, Yamada K, Onishi S, Moriguchi T, Sugita K, et al. Predictors of a successful outcome for infants with short bowel syndrome: a 30-year single-institution experience. Surg Today. Japan; 2017;47:1391–6.

41. Koglmeier J, Day C, Puntis JWL. Clinical outcome in patients from a single region who were dependent on parenteral nutrition for 28 days or more. Arch Dis Child. England; 2008;93:300–2.

42. Auth M, Garrett H, Jones E, Minford J. Successful management of infants with intestinal failure over two decades. J Pediatr Gastroenterol Nutr. 62:889.

43. Dore M, Junco PT, Moreno AA, Cerezo VN, Muñoz MR, Galán AS, Sánchez AV, Prieto G, Ramos E, Hernandez F, et al. Ultrashort Bowel Syndrome Outcome in Children Treated in a Multidisciplinary Intestinal Rehabilitation Unit. Eur J Pediatr Surg. 2017;27:116–20.

44. Khalil BA, Ba'ath ME, Aziz A, Forsythe L, Gozzini S, Murphy F, Carlson G, Bianchi A, Morabito A. Intestinal rehabilitation and bowel reconstructive surgery: improved outcomes in children with short bowel syndrome. J Pediatr Gastroenterol Nutr. United States; 2012;54:505–9.

45. Ives GC, Demehri FR, Sanchez R, Barrett M, Gadepalli S, Teitelbaum DH. Small Bowel Diameter in Short Bowel Syndrome as a Predictive Factor for Achieving Enteral Autonomy. J Pediatr. Elsevier Inc.; 2016;178:275–277.e1.

46. Demehri FR, Stephens L, Herrman E, West B, Mehringer A, Arnold MA, Brown PI, Teitelbaum DH. Enteral autonomy in pediatric short bowel syndrome: Predictive factors one year after diagnosis. J Pediatr Surg. Elsevier Inc.; 2015;50:131–5.

47. Hess RA, Welch KB, Brown PI, Teitelbaum DH. Survival outcomes of pediatric intestinal failure patients: Analysis of factors contributing to improved survival over the past two decades. J Surg Res. Elsevier Inc; 2011;170:27–31.

48. Spencer AU, Neaga A, West B, Safran J, Brown P, Btaiche I, Kuzma-O'Reilly B, Teitelbaum DH. Pediatric short bowel syndrome: redefining predictors of success. Ann Surg. United States; 2005;242:403–12.

49. Coran AG, Spivak D, Teitelbaum DH. An analysis of the morbidity and mortality of short-bowel syndrome in the pediatric age group. Eur J Pediatr Surg Off J Austrian Assoc Pediatr Surg . [et al] = Zeitschrift fur Kinderchirurgie. United States; 1999;9:228–30.

50. Teitelbaum DH, Drongowski R, Spivak D. Rapid development of hyperbilirubinemia in infants

with the short bowel syndrome as a correlate to mortality: possible indication for early small bowel transplantation. Transplant Proc. United States; 1996;28:2699–700.

51. Weber TR, Keller MS. Adverse effects of liver dysfunction and portal hypertension on intestinal adaptation in short bowel syndrome in children. Am J Surg. United States; 2002;184:582–6; discussion 586.

52. Weber TR, Tracy TJ, Connors RH. Short-bowel syndrome in children. Quality of life in an era of improved survival. Arch Surg. United States; 1991;126:841–6.

53. Furtado S, Ahmed N, Forget S, Sant'Anna A. Outcomes of Patients with Intestinal Failure after the Development and Implementation of a Multidisciplinary Team. Can J Gastroenterol Hepatol. Hindawi Publishing Corporation; 2016;2016.

54. Totonelli G, Tambucci R, Boscarelli A, Hermans D, Dall'Oglio L, Diamanti A, d'Aische A du B, Pakarinen M, Reding R, Morini F, et al. Pediatric Intestinal Rehabilitation and Transplantation Registry: Initial Report from a European Collaborative Registry. Eur J Pediatr Surg Off J Austrian Assoc Pediatr Surg . [et al] = Zeitschrift fur Kinderchirurgie. United States; 2018;28:75–80.

55. Squires RH, Duggan C, Teitelbaum DH, Wales PW, Balint J, Venick R, Rhee S, Sudan D, Mercer D, Martinez JA, et al. Natural history of pediatric intestinal failure: initial report from the Pediatric Intestinal Failure Consortium. J Pediatr. United States; 2012;161:723–8.e2.

56. Cole CR, Hansen NI, Higgins RD, Bell EF, Shankaran S, Laptook AR, Walsh MC, Hale EC, Newman NS, Das A, et al. Bloodstream infections in very low birth weight infants with intestinal failure. J Pediatr. 2012;160:54–9.e2.

57. Salvia G, Guarino A, Terrin G, Cascioli C, Paludetto R, Indrio F, Lega L, Fanaro S, Stronati M, Corvaglia L, et al. Neonatal onset intestinal failure: an Italian Multicenter Study. J Pediatr. United States; 2008;153:674–6, 676.e1-2.

58. Guarino A, De Marco G. Natural history of intestinal failure, investigated through a national network-based approach. J Pediatr Gastroenterol Nutr. United States; 2003;37:136–41.

59. Galea MH, Holliday H, Carachi R, Kapila L. Short-bowel syndrome: A collective review. J Pediatr Surg. 1992;27:592–6.

60. Infantino BJ, Mercer DF, Hobson BD, Fischer RT, Gerhardt BK, Grant WJ, Langnas AN, Quiros-Tejeira RE. Successful rehabilitation in pediatric ultrashort small bowel syndrome. J Pediatr. 2013;163:1361–6.

61. Bruzoni M, Sudan DL, Cusick RA, Thompson JS. Comparison of short bowel syndrome acquired early in life and during adolescence. Transplantation. 2008;86:63–6.

62. Thompson JS, Langnas AN, Pinch LW, Kaufman S, Quigley EM, Vanderhoof JA. Surgical approach to short-bowel syndrome. Experience in a population of 160 patients. Ann Surg. United States; 1995;222:600–7.

63. Norsa L, Artru S, Lambe C, Talbotec C, Pigneur B, Ruemmele F, Colomb V, Capito C, Chardot C, Lacaille F, et al. Long term outcomes of intestinal rehabilitation in children with neonatal very short bowel syndrome: Parenteral nutrition or intestinal transplantation. Clin Nutr. England; 2018;

64. Goulet O, Baglin-Gobet S, Talbotec C, Fourcade L, Colomb V, Sauvat F, Jais J-P, Michel J-L, Jan D, Ricour C. Outcome and long-term growth after extensive small bowel resection in the neonatal period: a survey of 87 children. Eur J Pediatr Surg Off J Austrian Assoc Pediatr Surg . [et al] = Zeitschrift fur Kinderchirurgie. United States; 2005;15:95–101.

65. Baglin-Gobet S, Jais JP, Dabbas M, Colomb V, Jan D, Jobert-Giraud A, Révillon Y, Ricour C, Goulet O. GROWTH AFTER NEONATAL SMALL BOWEL RESECTION. J Pediatr Gastroenterol Nutr. 1999;28.

66. Cowles RA, Ventura KA, Martinez M, Lobritto SJ, Harren PA, Brodlie S, Carroll J, Jan DM. Reversal of intestinal failure-associated liver disease in infants and children on parenteral

nutrition: experience with 93 patients at a referral center for intestinal rehabilitation. J Pediatr Surg. Elsevier Inc.; 2010;45:84–8.

67. Stollman TH, de Blaauw I, Wijnen MHWA, van der Staak FHJM, Rieu PNMA, Draaisma JMT, Wijnen RMH. Decreased mortality but increased morbidity in neonates with jejunoileal atresia; a study of 114 cases over a 34-year period. J Pediatr Surg. United States; 2009;44:217–21.

68. Phillips JD, Raval M V, Redden C, Weiner TM. Gastroschisis, atresia, dysmotility: surgical treatment strategies for a distinct clinical entity. J Pediatr Surg. United States; 2008;43:2208–12.

69. Kaufman SS, Loseke CA, Lupo J V, Young RJ, Murray ND, Pinch LW, Vanderhoof JA. Influence of bacterial overgrowth and intestinal inflammation on duration of parenteral nutrition in children with short bowel syndrome. J Pediatr. United States; 1997;131:356–61.

70. Ueno T, Wada M, Hoshino K, Matsuura T, Ida S, Okuyama H. Three-Year Prospective Follow-up of Potential Pediatric Candidate for Intestinal Transplantation. Transplant Proc. United States; 2018;50:2779–82.

71. Wasa M, Takagi Y, Sando K, Harada T, Okada A. Intestinal adaptation in pediatric patients with short-bowel syndrome. Eur J Pediatr Surg Off J Austrian Assoc Pediatr Surg . [et al] = Zeitschrift fur Kinderchirurgie. United States; 1999;9:207–9.

72. Fusaro F, Gamba PG, Orzali A, De Coppi P, Scarpa MG, Zanon GF. Vascular access in patients affected by short bowel syndrome. J Vasc Access. United States; 2000;1:33–5.

73. Cooper A, Floyd TF, Ross AJ, Bishop HC, Templeton JM, Ziegler MM. Morbidity and mortality of short-bowel syndrome acquired in infancy: An update. J Pediatr Surg. 1984;19:711–8.

74. Nucci A, Burns RC, Armah T, Lowery K, Yaworski JA, Strohm S, Bond G, Mazariegos G, Squires R. Interdisciplinary management of pediatric intestinal failure: a 10-year review of rehabilitation and transplantation. J Gastrointest Surg. United States; 2008;12:426–9.

75. Koehler AN, Yaworski JA, Gardner M, Kocoshis S, Reyes J, Barksdale EMJ. Coordinated interdisciplinary management of pediatric intestinal failure: a 2-year review. J Pediatr Surg. United States; 2000;35:380–5.

76. Kurkchubasche AG, Rowe MI, Smith SD. Adaptation in short-bowel syndrome: reassessing old limits. J Pediatr Surg. United States; 1993;28:1069–71.

77. Javid PJ, Malone FR, Bittner R, Healey PJ, Horslen SP. The optimal timing of referral to an intestinal failure program: the relationship between hyperbilirubinemia and mortality. J Pediatr Surg. United States; 2011;46:1052–6.

78. Wada M, Nishi K, Nakamura M, Kudo H, Yamaki S, Sasaki H, Sato T, Fukuzawa T, Tanaka H, Kazama T, et al. Life-threatening risk factors and the role of intestinal transplantation in patients with intestinal failure. Pediatr Surg Int. Germany; 2013;29:1115–8.

79. Dimatatac DM, Laksmi NK, Qi T. Predicting outcome of neonatal short bowel syndrome. J Pediatr Gastroenterol Nutr. 63:S266.

80. Pickard SS, Feinstein JA, Popat RA, Huang L, Dutta S. Short- and long-term outcomes of necrotizing enterocolitis in infants with congenital heart disease. Pediatrics. United States; 2009;123:e901-6.

81. Tillman EM, Norman JL, Huang EY, Lazar LF, Crill CM. Evaluation of parenteral nutrition-associated liver disease in infants with necrotizing enterocolitis before and after the implementation of feeding guidelines. Nutr Clin Pract. United States; 2014;29:234–7.

82. McLaughlin CM, Channabasappa N, Pace J, Nguyen H, Piper HG. Growth Trajectory in Children With Short Bowel Syndrome During the First 2 Years of Life. J Pediatr Gastroenterol Nutr. United States; 2018;66:484–8.

83. D. A, J. V, D. S, N. M. Morbidity and mortality of short bowel syndrome in infancy and childhood. Eur J Pediatr Surg. 1991;1:273–6.

84. Belza C, Fitzgerald K, de Silva N, Avitzur Y, Steinberg K, Courtney-Martin G, Wales PW. Predicting Intestinal Adaptation in Pediatric Intestinal Failure: A Retrospective Cohort Study. Ann Surg. United States; 2017;

85. So S, Patterson C, Gold A, Rogers A, Kosar C, de Silva N, Burghardt KM, Avitzur Y, Wales PW. Early neurodevelopmental outcomes of infants with intestinal failure. Early Hum Dev. Ireland; 2016;101:11–6.

86. So S, Patterson C, Gold A, Rogers A, Belza C, de Silva N, Avitzur Y, Wales PW. Neurodevelopmental outcomes of infants with intestinal failure at 12 and 26months corrected age. Early Hum Dev. Ireland; 2019;130:38–43.

87. Thacker K, Sukkar G, De-Silva N, Wales P, Avitzur Y. Long term outcome of children with ultrashort small bowel syndrome-a new reality with improved outcome. J Gastroenterol Hepatol. 30:170.

88. Burghardt KM, Wales PW, De Silva N, Stephens D, Yap J, Grant D, Avitzur Y. Pediatric intestinal transplant listing criteria - A call for a change in the new era of intestinal failure outcomes. Am J Transplant. 2015;15:1674–81.

89. Elfvin A, Dinsdale E, Wales PW, Moore AM. Low birthweight, gestational age, need for surgical intervention and gram-negative bacteraemia predict intestinal failure following necrotising enterocolitis. Acta Paediatr Int J Paediatr. 2015;104:771–6.

90. Burghardt KM, Avinashi V, Kosar C, Xu W, Wales PW, Avitzur Y, Muise A. A CARD9 polymorphism is associated with decreased likelihood of persistent conjugated hyperbilirubinemia in intestinal failure. PLoS One. 2014;9:1–6.

91. Diamond IR, Struijs MC, de Silva NT, Wales PW. Does the colon play a role in intestinal adaptation in infants with short bowel syndrome? A multiple variable analysis. J Pediatr Surg. Elsevier Inc.; 2010;45:975–9.

92. Diamond IR, de Silva N, Pencharz PB, Kim JH, Wales PW. Neonatal short bowel syndrome outcomes after the establishment of the first Canadian multidisciplinary intestinal rehabilitation program: preliminary experience. J Pediatr Surg. United States; 2007;42:806–11.

93. Nasr A, Avitzur Y, Ng VL, De Silva N, Wales PW. The use of conjugated hyperbilirubinemia greater than 100 micromol/L as an indicator of irreversible liver disease in infants with short bowel syndrome. J Pediatr Surg. United States; 2007;42:359–62.

94. Wales PW, de Silva N, Kim J, Lecce L, To T, Moore A. Neonatal short bowel syndrome: population-based estimates of incidence and mortality rates. J Pediatr Surg. 2004;39:690–5.

95. Bagwell CE, Filler RM, Cutz E, Stringer D, Ein SH, Shandling B, Stephens CA, Wesson DE. Neonatal intestinal pseudoobstruction. J Pediatr Surg. 1984;19:732–9.

96. Quiros-Tejeira RE, Ament ME, Reyen L, Herzog F, Merjanian M, Olivares-Serrano N, Vargas JH. Long-term parenteral nutritional support and intestinal adaptation in children with short bowel syndrome: a 25-year experience. J Pediatr. United States; 2004;145:157–63.

97. Thakur A, Chiu C, Quiros-Tejeira RE, Reyen L, Ament M, Atkinson JB, Fonkalsrud EW. Morbidity and mortality of short-bowel syndrome in infants with abdominal wall defects. Am Surg. United States; 2002;68:75–9.

98. Angsten G, Finkel Y, Lucas S, Kassa AM, Paulsson M, Lilja HE. Improved outcome in neonatal short bowel syndrome using parenteral fish oil in combination with ω-6/9 lipid emulsions. J Parenter Enter Nutr. 2012;36:587–95.

99. Vugrinec Mamic M, Hojsak I, Misak Z, Kerman V, Kolacek S. Treatment in a Tertiary Intestinal Rehabilitation Center Improves Outcome for Children With Short Bowel Syndrome. Gastroenterol Nurs. United States; 2018;

100. Rajanayagam J, Beath S, Gowen H, Hartley J, Sharif K, Muiesan P, Lloyd C, Mirza DF, Gupte GL. 24-year outcomes of pediatric intestinal failure associated liver disease-experience of a transplant centre. Transplantation. 1:S109.

101. Nandivada P, Baker MA, Mitchell PD, O'Loughlin AA, Potemkin AK, Anez-Bustillos L, Carlson SJ, Dao DT, Fell GL, Gura KM, et al. Predictors of failure of fish-oil therapy for intestinal failure-associated liver disease in children. Am J Clin Nutr. United States; 2016;104:663–70.

102. Nandivada P, Fell GL, Mitchell PD, Potemkin AK, O'Loughlin AA, Gura KM, Puder M. Long-Term Fish Oil Lipid Emulsion Use in Children With Intestinal Failure-Associated Liver Disease [Formula: see text]. JPEN J Parenter Enteral Nutr. United States; 2017;41:930–7.

103. Martinez MI, Rumbo C, ez A, Niveyro S, Saa G, Gondolesi GE. Clinical course of pediatric patients with intestinal failure associated liver disease. Transplantation. 1:S30–1.

104. De Marco G, Sordino D, Bruzzese E, Di Caro S, Mambretti D, Tramontano A, Colombo C, Simoni P, Guarino A. Early treatment with ursodeoxycholic acid for cholestasis in children on parenteral nutrition because of primary intestinal failure. Aliment Pharmacol Ther. 2006;24:387–94.

105. Iyer KR, Horslen S, Torres C, Vanderhoof JA, Langnas AN, Tracy T. Functional Liver Recovery Parallels Autologous Gut Salvage in Short Bowel Syndrome. J Pediatr Surg. 2004;39:340–4.

106. Colomb V, Goulet O, De Potter S, Ricour C. Liver disease associated with long-term parenteral nutrition in children. Transpl Proc. 1994/06/01. 1994;26:1467.

107. Sorrell M, Moreira A, Green K, Jacob R, Tragus R, Keller L, Quinn A, McCurnin D, Gong A, El Sakka A, et al. Favorable Outcomes of Preterm Infants With Parenteral Nutrition-associated Liver Disease Treated With Intravenous Fish Oil-based Lipid Emulsion. J Pediatr Gastroenterol Nutr. United States; 2017;64:783–8.

108. Nasr A, Diamond IR, de Silva NT, Wales PW. Is the use of parenteral omega-3 lipid emulsions justified in surgical neonates with mild parenteral nutrition-associated liver dysfunction? J Pediatr Surg. United States; 2010;45:980–6.

109. Wang C, Venick RS, Shew SB, Dunn JCY, Reyen L, Gou R, Calkins KL. Long-Term Outcomes in Children With Intestinal Failure-Associated Liver Disease Treated With 6 Months of Intravenous Fish Oil Followed by Resumption of Intravenous Soybean Oil. JPEN J Parenter Enteral Nutr. United States; 2018;

110. Spurrier R, Grant CN, Yan PY, Grikscheit T, Merritt R. Parenteral fish oil reverses cholestasis in parenteral nutrition associated liver disease. Gastroenterology. 1:S1027.

111. Rollins MD, Scaife ER, Jackson WD, Meyers RL, Mulroy CW, Book LS. Elimination of soybean lipid emulsion in parenteral nutrition and supplementation with enteral fish oil improve cholestasis in infants with short bowel syndrome. Nutr Clin Pract. United States; 2010;25:199–204.

112. Kaufman SS, Pehlivanova M, Fennelly EM, Rekhtman YM, Gondolesi GE, Little CA, Matsumoto CS, Fishbein TM. Predicting liver failure in parenteral nutrition-dependent short bowel syndrome of infancy. J Pediatr. United States; 2010;156:580–5.e1.

113. Simmons MG, Georgeson KE, Figueroa R, Mock DL 2nd. Liver failure in parenteral nutrition-dependent children with short bowel syndrome. Transplant Proc. United States; 1996;28:2701.

114. Vegting IL, Tabbers MM, Benninga MA, Wilde JC, Serlie MJ, Tas TA, Jonkers CF, van Ommen CH. Prophylactic anticoagulation decreases catheter-related thrombosis and occlusion in children with home parenteral nutrition. JPEN J Parenter Enteral Nutr. United States; 2012;36:456–62.

115. Kostrzewski A, Herbison K, Dijkstra K, Dean R, McLeod B, Evans H. Intestinal failure in paediatric inpatients: A five year audit at a tertiary children's hospital in New Zealand. J Pediatr Gastroenterol Nutr. 64:986.

116. Diamanti A, Conforti A, Panetta F, Torre G, Candusso M, Bagolan P, Papa RE, Grimaldi C, Fusaro F, Capriati T, et al. Long-term outcome of home parenteral nutrition in patients with ultra-short bowel syndrome. J Pediatr Gastroenterol Nutr. United States; 2014;58:438–42.

117. Diamanti A, Panetta F, Gandullia P, Morini F, Noto C, Torre G, Lezo A, Goffredo B, Daniele A, Gambarara M. Plasma citrulline as marker of bowel adaptation in children with short bowel syndrome. Langenbeck's Arch Surg. Germany; 2011;396:1041–6.

118. Knafelz D, Gambarara M, Diamanti A, Papadatou B, Ferretti F, Tarissi De Iacobis I, Castro M. Complications of home parenteral nutrition in a large pediatric series. Transplant Proc. United States; 2003;35:3050–1.

119. Gambarara M, Ferretti F, Papadatou B, Diamanti A, Castro M. Central vein catheter-related complications associated with home parenteral nutrition in children: experience in 41 patients. Nutrition. United States; 2001;17:970–1.

120. Gandullia P, Lugani F, Costabello L, Arrigo S, Calvi A, Castellano E, Vignola S, Pistorio A, Barabino A V. Long-term home parenteral nutrition in children with chronic intestinal failure: A 15-year experience at a single Italian centre. Dig Liver Dis. Editrice Gastroenterologica Italiana; 2011;43:28–33.

121. Dobson R, McGuckin C, Walker G, Lucas C, McGrogan P, Russell RK, Young D, Flynn DM, Barclay AR. Cycled enteral antibiotics reduce sepsis rates in paediatric patients on long-term parenteral nutrition for intestinal failure. Aliment Pharmacol Ther. 2011;34:1005–11.

122. Ashworth I, Wilson A, Aquilina S, Parascandalo R, Mercieca V, Gerada J, Macdonald S, Simchowitz V, Hill S. Reversal of Intestinal Failure in Children With Tufting Enteropathy Supported With Parenteral Nutrition at Home. J Pediatr Gastroenterol Nutr. United States; 2018;66:967–71.

123. Chu HP, Brind J, Tomar R, Hill S. Significant reduction in central venous catheter-related bloodstream infections in children on HPN after starting treatment with taurolidine line lock. J Pediatr Gastroenterol Nutr. 2012;55:403–7.

124. Sala D, Chomto S, Hill S. Long-term outcomes of short bowel syndrome requiring long-term/home intravenous nutrition compared in children with gastroschisis and those with volvulus. Transplant Proc. United States; 2010;42:5–8.

125. Bisset WM, Stapleford P, Long S, Chamberlain A, Sokel B, Milla PJ. Home parenteral nutrition in chronic intestinal failure Unit, Hospital for Sick Children, London. Arch ofDisease Child. 1992;67:109–14.

126. Peyret B, Collardeau S, Touzet S, Loras-Duclaux I, Yantren H, Michalski MC, Chaix J, Restier-Miron L, Bouvier R, Lachaux A, et al. Prevalence of liver complications in children receiving long-term parenteral nutrition. Eur J Clin Nutr. England; 2011;65:743–9.

127. Styblova J, Kalousova J, Adamcova M, Bajerova K, Bronsky J, Fencl F, Karaskova E, Keslova P, Melek J, Pozler O, et al. Paediatric Home Parenteral Nutrition in the Czech Republic and Its Development: Multicentre Retrospective Study 1995-2011. Ann Nutr Metab. Switzerland; 2017;71:99–106.

128. Goulet O. Home parenteral nutrition (home-PN) in France: A national survey. Clin Nutr. 35:S235.

129. Neelis EG, Roskott AM, Dijkstra G, Wanten GJ, Serlie MJ, Tabbers MM, Damen G, Olthof ED, Jonkers CF, Kloeze JH, et al. Presentation of a nationwide multicenter registry of intestinal failure and intestinal transplantation. Clin Nutr. England; 2016;35:225–9.

130. Zamvar V, Puntis JWL, Gupte G, Lazonby G, Holden C, Sexton E, Bunford C, Protheroe S, Beath S V. Social circumstances and medical complications in children with intestinal failure. Arch Dis Child. England; 2014;99:336–41.

131. Gojda J, Jirka A, Strenkova J, Jarkovsky J, Tesinsky P. 20 years of home parenteral nutrition national registry in Czech Republic. Clin Nutr. 32:S90.

132. Pironi L, Joly F, Forbes A, Colomb V, Lyszkowska M, Baxter J, Gabe S, Hebuterne X, Gambarara M, Gottrand F, et al. Long-term follow-up of patients on home parenteral nutrition in Europe: implications for intestinal transplantation. Gut. England; 2011;60:17–25.

133. Lambe C, Poisson C, Talbotec C, Goulet O. Strategies to Reduce Catheter-Related Bloodstream Infections in Pediatric Patients Receiving Home Parenteral Nutrition: The Efficacy of Taurolidine-Citrate Prophylactic-Locking. JPEN J Parenter Enteral Nutr. United States; 2018;42:1017–25.

134. Petit L-M, Girard D, Ganousse-Mazeron S, Talbotec C, Pigneur B, Elie C, Corriol O, Poisson C, Goulet O, Colomb V. Weaning Off Prognosis Factors of Home Parenteral Nutrition for Children With Primary Digestive Disease. J Pediatr Gastroenterol Nutr. United States; 2016;62:462–8.

135. Abi Nader E, Lambe C, Talbotec C, Pigneur B, Lacaille F, Garnier-Lengline H, Petit L-M, Poisson C, Rocha A, Corriol O, et al. Outcome of home parenteral nutrition in 251 children over a 14-y period: report of a single center. Am J Clin Nutr. United States; 2016;103:1327–36.

136. Artru S, Lacaille F, Lambe C, Chardot C, Pigneur B, Lengline H, Gastineau S, Talbotec C, Ruemmele F, Colomb V, et al. Ultra short bowel syndrome (USBS) in children: long-term parenteral nutrition in comparison with intestinal transplantation. J Pediatr Gastroenterol Nutr. 2014;58:250.

137. Halac U, Lacaille F, Joly F, Hugot JP, Talbotec C, Colomb V, Ruemmele FM, Goulet O. Microvillous inclusion disease: How to improve the prognosis of a severe congenital enterocyte disorder. J Pediatr Gastroenterol Nutr. 2011;52:460–5.

138. Colomb ÃV, Dabbas-tyan ÃM, Taupin P, Ce Ã, Re Y, Jan D, Potter ÃS De, Miche Ã, Herreman ÃK, Corriol O, et al. Long-term Outcome of Children Receiving Home Parenteral Nutrition : A 20-year Single-center Experience in 302 Patients. 2007;347–53.

139. Colomb V, Goulet O, Rambaud C, De Potter S, Sadoun E, Ben Hariz M, Jan D, Brousse N, Ricour C. Long-term parenteral nutrition in children: liver and gallbladder disease. Transplant Proc. United States; 1992;24:1054–5.

140. De Potter S, Goulet O, Lamor M, Corriol O, Colomb V, Sadoun E, Ricour C. 263 patient-years of home parenteral nutrition in children. Transplant Proc. United States; 1992;24:1056–7.

141. Ricour C, Gorski a M, Goulet O, de Potter S, Corriol O, Postaire M, Nihoul-Fekete C, Jan D, Revillon Y, Lortat-Jacob S, et al. Home parenteral nutrition in children: 8 years of experience with 112 patients. Clin Nutr. 1990;9:65–71.

142. Ricour C, Duhamel JF, Arnaud-Battandier F, Collard Y, Revillon Y, Nihoul-Fekete C. [Extensive resection of the small intestine in children]. Arch Fr Pediatr. France; 1985;42:285–90.

143. Schmidt-Sommerfeld E, Snyder G, Rossi TM, Lebenthal E. Catheter-related complications in 35 children and adolescents with gastrointestinal disease on home parenteral nutrition. JPEN J Parenter Enteral Nutr. United States; 1990;14:148–51.

144. Rhoads JM, Plunkett E, Galanko J, Lichtman S, Taylor L, Maynor A, Weiner T, Freeman K, Guarisco JL, Wu GY. Serum citrulline levels correlate with enteral tolerance and bowel length in infants with short bowel syndrome. J Pediatr. United States; 2005;146:542–7.

145. Pharaon I, Despres C, Aigrain Y, Grini A, Faure C, Matarazzo P, Navarro J, Cathelineau L, Cezard JP. Long-term parenteral nutrition in children who are potentially candidates for small bowel transplantation. Transplant Proc. United States; 1994;26:1442.

146. Neelis E, Olieman J, Rizopoulos D, Wijnen R, Rings E, de Koning B, Hulst J. Growth, Body Composition and Micronutrient Abnormalities During and After Weaning Off Home Parenteral Nutrition. J Pediatr Gastroenterol Nutr. United States; 2018;

147. Choi SJ, Lee KJ, Choi JS, Yang HR, Moon JS, Chang JY, Ko JS. Poor Prognostic Factors in Patients with Parenteral Nutrition-Dependent Pediatric Intestinal Failure. Pediatr Gastroenterol Hepatol Nutr. 2016;19:44.

148. Brown SK, Davies N, Smyth E, Heather N, Cole C, Keys SC, Beattie RM, Batra A. Intestinal Failure: The Evolving Demographic and Patient Outcomes on Home Parenteral Nutrition. Acta Paediatr. 2018;doi: 10.1111/apa.14401 [Epub ahead of print].

149. Wiskin AE, Cole C, Owens DR, Morgan M, Burge DM, Beattie RM. Ten-year experience of home parenteral nutrition in a single centre. Acta Paediatr. Norway; 2012;101:524–7.

150. Pieroni KP, Nespor C, Ng M, Garcia M, Hurwitz M, Berquist WE, Kerner JA. Evaluation of ethanol lock therapy in pediatric patients on long-term parenteral nutrition. Nutr Clin Pract. 2013;28:226–31.

151. Tung Y-C, Ni Y-H, Lai H-S, Hsieh D-Y, Chang M-H. Clinical improvement following home parenteral nutrition in pediatric patients with intestinal failure. J Formos Med Assoc. Singapore; 2006;105:399–403.

152. Candusso M, Giglio L, Faraguna D. 100 PT/YR pediatric home parenteral nutrition experience. Transplant Proc. United States; 1997;29:1864–5.

153. Moukarzel AA, Haddad I, Ament ME, Buchman AL, Reyen L, Maggioni A, Baron HI, Vargas J. 230 patient years of experience with home long-term parenteral nutrition in childhood: natural history and life of central venous catheters. J Pediatr Surg. United States; 1994;29:1323–7.

154. Vargas JH, Ament ME, Berquist WE. Long-term home parenteral nutrition in pediatrics: ten years of experience in 102 patients. J Pediatr Gastroenterol Nutr. United States; 1987;6:24–32.

155. S.F.A. D, M.E. A. Improved survival in very short small bowel of infancy with use of long-term parenteral nutrition. J Pediatr. 1985;107:521–5.

156. Olszewska K, Ksiazyk J, Kozlowski D, Pajdowska M, Janusz M, Jaworski M. Nutritional therapy complications in children with ultra-short bowel syndrome include growth deficiency but not cholestasis. Acta Paediatr. Norway; 2018;107:1088–93.

157. Friedman-Gruszczyńska J, Ossolińska M, Popińska K, Ksiazyk JB. Parenteral nutrition mixtures prepared at home by trained parents are as safe as pharmacy-made mixtures: A 3-y prospective study. Nutrition. 2013;29:988–92.

158. Ksiazyk J, Lyszkowska M, Kierkus J, Bogucki K, Ratynska A, Tondys B, Socha J. Home parenteral nutrition in children: the Polish experience. J Pediatr Gastroenterol Nutr. United States; 1999;28:152–6.

159. Nusinovich Y, Revenis M, Torres C. Long-term outcomes for infants with intestinal atresia studied at Children's National Medical Center. J Pediatr Gastroenterol Nutr. United States; 2013;57:324–9.

160. Gupte GL, Beath S V., Protheroe S, Murphy MS, Davies P, Sharif K, McKiernan PJ, De Goyet JDV, Booth IW, Kelly DA. Improved outcome of referrals for intestinal transplantation in the UK. Arch Dis Child. 2007;92:147–52.

161. Kelly D. Transplantation--new beginnings and new horizons. J Pediatr Gastroenterol Nutr. United States; 2002;34 Suppl 1:S51-3.

162. Beath S V, Booth IW, Murphy MS, Buckels J a, Mayer a D, McKiernan PJ, Kelly D a. Nutritional care and candidates for small bowel transplantation. Arch Dis Child. 1995;73:348–50.

163. Varkey J, Simren M, Bosaeus I, Krantz M, Gabel M, Herlenius G. Survival of patients evaluated for intestinal and multivisceral transplantation - the Scandinavian experience. Scand J Gastroenterol. England; 2013;48:702–11.

164. Chapman B, De Cruz P, Jones R, Hardikar W, Testro A. Intestinal Failure and Transplant: The Australian Experience (2009 to 2014). Transplant Proc. Elsevier Inc; 2016;48:463–7.

165. Torres C, Sudan D, Vanderhoof J, Grant W, Botha J, Raynor S, Langnas A. Role of an intestinal rehabilitation program in the treatment of advanced intestinal failure. J Pediatr Gastroenterol Nutr. United States; 2007;45:204–12.

166. Ganousse-Mazeron S, Lacaille F, Colomb-Jung V, Talbotec C, Ruemmele F, Sauvat F, Chardot C, Canioni D, Jan D, Revillon Y, et al. Assessment and outcome of children with

intestinal failure referred for intestinal transplantation. Clin Nutr. Elsevier Ltd; 2015;34:428–35.

167. Ruemmele FM, Jan D, Lacaille F, Cezard J-P, Canioni D, Phillips AD, Peuchmaur M, Aigrain Y, Brousse N, Schmitz J, et al. New perspectives for children with microvillous inclusion disease: early small bowel transplantation. Transplantation. United States; 2004;77:1024–8.

168. Hugar LA, Chaudhry R, Fuller TW, Cannon GM, Schneck FX, Ost MC, Stephany HA. Urologic Phenotype and Patterns of Care in Patients With Megacystis Microcolon Intestinal Hypoperistalsis Syndrome Presenting to a Major Pediatric Transplantation Center. Urology. United States; 2018;119:127–32.

169. Sigurdsson L, Reyes J, Kocoshis SA, Mazariegos G, Abu-Elmagd KM, Bueno J, Di Lorenzo C. Intestinal transplantation in children with chronic intestinal pseudo-obstruction. Gut. England; 1999;45:570–4.

170. Avitzur Y, Wang JY, De Silva NT, Burghardt KM, Deangelis M, Grant D, Ng VL, Jones N, Wales PW. Impact of Intestinal Rehabilitation Program and Its Innovative Therapies on the Outcome of Intestinal Transplant Candidates. J Pediatr Gastroenterol Nutr. 2015;61:18–23.

171. Fecteau A, Atkinson P, Grant D. Early referral is essential for successful pediatric small bowel transplantation: The canadian experience. J Pediatr Surg. 2001;36:681–4.

172. Garnett GM, Kang KH, Jaksic T, Woo RK, Puapong DP, Kim HB, Johnson SM. First STEPs: Serial transverse enteroplasty as a primary procedure in neonates with congenital short bowel. J Pediatr Surg. Elsevier Inc.; 2014;49:104–8.

173. Ching YA, Fitzgibbons S, Valim C, Zhou J, Duggan C, Jaksic T, Kim HB. Long-term nutritional and clinical outcomes after serial transverse enteroplasty at a single institution. J Pediatr Surg. 2009;44:939–43.

174. Bishay M, Pichler J, Horn V, MacDonald S, Ellmer M, Eaton S, Hill S, Pierro A. Intestinal failure-associated liver disease in surgical infants requiring long-term parenteral nutrition. J Pediatr Surg. Elsevier Inc.; 2012;47:359–62.

175. Reinshagen K, Zahn K, Buch C von, Zoeller M, Hagl CI, Ali M, Waag K-L. The impact of longitudinal intestinal lengthening and tailoring on liver function in short bowel syndrome. Eur J Pediatr Surg Off J Austrian Assoc Pediatr Surg . [et al] = Zeitschrift fur Kinderchirurgie. United States; 2008;18:249–53.

176. Hosie S, Loff S, Wirth H, Rapp HJ, Von Buch C, Waag KL. Experience of 49 longitudinal intestinal lengthening procedures for short bowel syndrome. Eur J Pediatr Surg. 2006;16:171–5.

177. Waag KL, Hosie S, Wessel L. What do children look like after longitudinal intestinal lengthening. Eur J Pediatr Surg Off J Austrian Assoc Pediatr Surg . [et al] = Zeitschrift fur Kinderchirurgie. United States; 1999;9:260–2.

178. Hukkinen M, Merras-Salmio L, Sipponen T, Mutanen A, Rintala RJ, Mäkisalo H, Pakarinen MP. Surgical rehabilitation of short and dysmotile intestine in children and adults. Scand J Gastroenterol. 2015;50:153–61.

179. Dore M, Junco PT, Andres AM, Sanchez-Galan A, Amesty MV, Ramos E, Prieto G, Hernandez F, Lopez Santamaria M. Surgical Rehabilitation Techniques in Children with Poor Prognosis Short Bowel Syndrome. Eur J Pediatr Surg Off J Austrian Assoc Pediatr Surg . [et al] = Zeitschrift fur Kinderchirurgie. United States; 2016;26:112–6.

180. Pederiva F, Sgro A, Coletta R, Khalil B, Morabito A. Outcomes in patients with short bowel syndrome after autologous intestinal reconstruction: Does etiology matter? J Pediatr Surg. United States; 2018;53:1345–50.

181. Bianchi A. Longitudinal intestinal lengthening and tailoring: results in 20 children. J R Soc Med. England; 1997;90:429–32.

182. Barrett M, Demehri FR, Ives GC, Schaedig K, Arnold MA, Teitelbaum DH. Taking a STEP back: Assessing the outcomes of multiple STEP procedures. J Pediatr Surg. Elsevier B.V.;

2017;52:69–73.

183. Miyasaka EA, Brown PI, Teitelbaum DH. Redilation of bowel after intestinal lengthening procedures--an indicator for poor outcome. J Pediatr Surg. United States; 2011;46:145–9.

184. Weber TR. Isoperistaltic bowel lengthening for short bowel syndrome in children. Am J Surg. United States; 1999;178:600–4.

185. Jones BA, Hull MA, Potanos KM, Zurakowski D, Fitzgibbons SC, Ching YA, Duggan C, Jaksic T, Kim HB. Report of 111 consecutive patients enrolled in the International Serial Transverse Enteroplasty (STEP) Data Registry: a retrospective observational study. J Am Coll Surg. United States; 2013;216:438–46.

186. Wood SJ, Khalil B, Fusaro F, Folaranmi SE, Sparks SA, Morabito A. Early structured surgical management plan for neonates with short bowel syndrome may improve outcomes. World J Surg. United States; 2013;37:1714–7.

187. Modi BP, Javid PJ, Jaksic T, Piper H, Langer M, Duggan C, Kamin D, Kim HB. First report of the international serial transverse enteroplasty data registry: indications, efficacy, and complications. J Am Coll Surg. United States; 2007;204:365–71.

188. Mercer DF, Hobson BD, Gerhardt BK, Grant WJ, Vargas LM, Langnas AN, Quiros-Tejeira RE. Serial transverse enteroplasty allows children with short bowel to wean from parenteral nutrition. J Pediatr. United States; 2014;164:93–8.

189. Andres AM, Thompson J, Grant W, Botha J, Sunderman B, Antonson D, Langnas A, Sudan D. Repeat surgical bowel lengthening with the STEP procedure. Transplantation. 2008;85:1294–9.

190. Sudan D, Thompson J, Botha J, Grant W, Antonson D, Raynor S, Langnas A. Comparison of intestinal lengthening procedures for patients with short bowel syndrome. Ann Surg. United States; 2007;246:593–4.

191. Thompson JS, Pinch LW, Young R, Vanderhoof JA. Long-term outcome of intestinal lengthening. Transplant Proc. United States; 2000;32:1242–3.

192. Fujioka WK, Cowles RA. Infectious complications following serial transverse enteroplasty in infants and children with short bowel syndrome. J Pediatr Surg. Elsevier Inc.; 2015;50:428–30.

193. Oh PS, Fingeret AL, Shah MY, Ventura KA, Brodlie S, Ovchinsky N, Martinez M, Lobritto SJ, Cowles RA. Improved tolerance for enteral nutrition after serial transverse enteroplasty (STEP) in infants and children with short bowel syndrome--a seven-year single-center experience. J Pediatr Surg. United States; 2014;49:1589–92.

194. Walker SR, Nucci A, Yaworski JA, Barksdale EMJ. The Bianchi procedure: a 20-year single institution experience. J Pediatr Surg. United States; 2006;41:113–9.

195. Bueno J, Guiterrez J, Mazariegos G V., Abu-Elmagd K, Madariaga J, Ohwada S, Kocoshis S, Reyes J. Analysis of patients with longitudinal intestinal lengthening procedure referred for intestinal transplantation. J Pediatr Surg. 2001;36:178–83.

196. Javid PJ, Sanchez SE, Horslen SP, Healey PJ. Intestinal lengthening and nutritional outcomes in children with short bowel syndrome. Am J Surg. United States; 2013;205:576–80.

197. Wester T, Borg H, Naji H, Stenstrom P, Westbacke G, Lilja HE. Serial transverse enteroplasty to facilitate enteral autonomy in selected children with short bowel syndrome. Br J Surg. England; 2014;101:1329–33.

198. Fitzgerald K, Muto M, Belza C, De Silva N, Avitzur Y, Wales PW. The evolution of the serial transverse enteroplasty for pediatric short bowel syndrome at a single institution. J Pediatr Surg. United States; 2019;

199. Oliveira C, de Silva N, Wales PW. Five-year outcomes after serial transverse enteroplasty in children with short bowel syndrome. J Pediatr Surg. United States; 2012;47:931–7.

200. Wales PW, de Silva N, Langer JC, Fecteau A. Intermediate outcomes after serial transverse

enteroplasty in children with short bowel syndrome. J Pediatr Surg. United States; 2007;42:1804–10.

201. Sehgal S, Sandler AD, Chahine AA, Mohan P, Torres C. Ostomy in continuity : A novel approach for the management of children with complex short bowel syndrome ☆ , ☆☆. J Pediatr Surg. Elsevier Inc.; 2018;53:1989–95.

202. Shah AA, Petrosyan M, Franklin AL, Chahine AA, Torres C, Sandler AD. Autologous intestinal reconstruction: a single institution study of the serial transverse enteroplasty (STEP) and the longitudinal intestinal lengthening and tailoring (LILT). Pediatr Surg Int. Germany; 2019;

203. Stanger JD, Oliveira C, Blackmore C, Avitzur Y, Wales PW. The impact of multi-disciplinary intestinal rehabilitation programs on the outcome of pediatric patients with intestinal failure: a systematic review and meta-analysis. J Pediatr Surg. United States; 2013;48:983–92.

204. Frongia G, Kessler M, Weih S, Nickkholgh A, Mehrabi A, Holland-Cunz S. Comparison of LILT and STEP procedures in children with short bowel syndrome - A systematic review of the literature. J Pediatr Surg. Elsevier Inc.; 2013;48:1794–805.

205. Oliveira C, Nasr A, Brindle M, Wales PW. Ethanol locks to prevent catheter-related bloodstream infections in parenteral nutrition: a meta-analysis. Pediatrics. United States; 2012;129:318–29.

206. Lauriti G, Zani A, Aufieri R, Cananzi M, Chiesa PL, Eaton S, Pierro A. Incidence, prevention, and treatment of parenteral nutrition-associated cholestasis and intestinal failure-associated liver disease in infants and children: A systematic review. J Parenter Enter Nutr. 2014;38:70–85.

207. Nakamura H, Henderson D, Puri P. A meta-analysis of clinical outcome of intestinal transplantation in patients with total intestinal aganglionosis. Pediatr Surg Int. Germany; 2017;33:837–41.

208. Belza C, Wales PW. Impact of multidisciplinary teams for management of intestinal failure in children. Curr Opin Pediatr. 2017;29:334–9.

209. Fernandes MA, Usatin D, Allen IE, Rhee S, Vu L. Improved enteral tolerance following step procedure: systematic literature review and meta-analysis. Pediatr Surg Int. Springer Berlin Heidelberg; 2016;32:921–6.

210. Reddy VS, Patole SK, Rao S. Role of probiotics in short bowel syndrome in infants and children-A systematic review. Nutrients. 2013;5:679–99.

211. King B, Carlson G, Khalil BA, Morabito A. Intestinal bowel lengthening in children with short bowel syndrome: systematic review of the Bianchi and STEP procedures. World J Surg. United States; 2013;37:694–704.

212. Duran B. The effects of long-term total parenteral nutrition on gut mucosal immunity in children with short bowel syndrome: A systematic review. BMC Nurs. 2005;4.

213. Bergholz R, Boettcher M, Reinshagen K, Wenke K. Complex gastroschisis is a different entity to simple gastroschisis affecting morbidity and mortality-a systematic review and meta-analysis. J Pediatr Surg. United States; 2014;49:1527–32.

214. Barclay AR, Beattie LM, Weaver LT, Wilson DC. Systematic review: Medical and nutritional interventions for the management of intestinal failure and its resultant complications in children. Aliment Pharmacol Ther. 2011;33:175–84.

215. Rangel SJ, Calkins CM, Cowles RA, Barnhart DC, Huang EY, Abdullah F, Arca MJ, Teitelbaum DH. Parenteral nutrition-associated cholestasis: An American pediatric surgical association outcomes and clinical trials committee systematic review. J Pediatr Surg. Elsevier Inc.; 2012;47:225–40.

216. Hau EM, Meyer SC, Berger S, Goutaki M, Kordasz M, Kessler U. Gastrointestinal sequelae after surgery for necrotising enterocolitis: A systematic review and meta-analysis. Arch Dis Child Fetal Neonatal Ed. 2018;1–9.

217. Richards DM, Deeks JJ, Sheldon TA, Shaffer JL. Home parenteral nutrition: a systematic review. Health Technol Assess. 1997;1:i–iii, 1-59.

218. Dicken BJ, Sergi C, Rescorla FJ, Breckler F, Sigalet D. Medical management of motility disorders in patients with intestinal failure: A focus on necrotizing enterocolitis, gastroschisis, and intestinal atresia. J Pediatr Surg. Elsevier Inc.; 2011;46:1618–30.

219. Seida JC, Mager DR, Hartling L, Vandermeer B, Turner JM. Parenteral ω-3 Fatty Acid Lipid Emulsions for Children With Intestinal Failure and Other Conditions. J Parenter Enter Nutr. Wiley-Blackwell; 2012;37:44–55.

220. Rahhal R, Abu-El-Haija MA, Fei L, Ebach D, Orkin S, Kiscaden E, Cole CR. Systematic Review and Meta-Analysis of the Utilization of Ethanol Locks in Pediatric Patients With Intestinal Failure. JPEN J Parenter Enteral Nutr. United States; 2018;42:690–701.

Supplemental methods

Full search term used:

(("intestinal failure" OR "IF" OR "short bowel syndrome" OR "SBS" OR "short gut") AND ("child" OR "children" OR "paediatric" OR "pediatric" OR "infant" OR "neonate" OR "premature") AND ("mortality" OR "outcome" OR "natural history" OR "parenteral nutrition" OR "PN" OR "TPN" OR "HPN" OR "enteral feeding" OR "enteral nutrition" OR "sepsis" OR "catheter related bloodstream infections" OR "CRBSI" OR "catheter related sepsis" OR "intestinal transplant" OR "small bowel transplant" OR "combined liver and small bowel transplant" OR "intestinal failure associated liver disease" OR "IFALD" OR "multivisceral transplant" OR “bloodstream infection” OR "parenteral nutrition associated liver disease" OR "PNALD" OR "parenteral nutrition associated cholestasis" OR "PNAC" OR “PICC” OR “Broviac” OR “Hickman” OR “thrombosis” OR “break” OR “breakage” OR “replacement”)) NOT "review"[Publication Type]

Search completed on 17th March 2019.
